# Oncolytic adenovirus type 11-induced ferroptosis of esophageal squamous cell carcinoma cells involves in mitochondrial impairment and the mTOR pathway

**DOI:** 10.1186/s12885-026-15735-7

**Published:** 2026-02-24

**Authors:** Lingling Si, Chengbin Zhao, Louisa S Chard Dunmall, Pengju Wang, Zhenguo Cheng, Yaohe Wang

**Affiliations:** 1 Sino-British Research Centre for Molecular Oncology, National Centre for International Research in Cell and Gene Therapy, State Key Laboratory of Metabolic Dysregulation & Prevention and Treatment of Esophageal Cancer, Tianjian Laboratory of Advanced Biomedical Sciences, School of Convergence Medicine, Zhengzhou University, Zhengzhou, 450052 China; 2 Department of Neurosurgery, The First Affiliated Hospital of Zhengzhou University, No. 1, Jianshe East Road, Erqi District, Zhengzhou, Henan 450053 China; 3 Centre for Cancer Biomarkers & Biotherapeutics, Barts Cancer Institute, Queen Mary University of London, London, EC1M 6BQ UK

**Keywords:** Oncolytic adenovirus type 11, Esophageal squamous cell carcinoma, Ferroptosis, Mitochondria, mTOR

## Abstract

**Background:**

Human adenovirus type 11 (HAdV-11) has several advantages compared with human adenovirus type 5 (HAdV-5), including improved infectivity of tumor cells and the potential for intravenous delivery. Our previous study demonstrated that HAdV-11 efficiently infected various human cancer cell lines including esophageal squamous cell carcinoma (ESCC) cell lines, however the cytotoxicity was not effective in some cell lines. The underlying functional mechanisms for this discrepancy are not clear.

**Methods:**

Cell viability was assessed using an 3-(4,5-dimethylthiazol-2-yl)-5-(3-carboxymethoxyphenyl)-2-(4-sulfophenyl)-2 H-tetrazolium (MTS) assay, and viral replication was quantified by tissue culture infectious dose 50 (TCID_50_) and quantitative polymerase chain reaction (qPCR). Flow cytometry was employed to analyze apoptosis, mitochondrial reactive oxygen species, lipid oxidation, mitochondrial mass, mitochondrial membrane potential, and intracellular Fe²⁺ levels. Transmission electron microscopy was used for ultrastructural analysis. Protein expression and mitochondrial localization were evaluated by Western blotting and immunofluorescence staining, while reduced glutathione levels were measured using a commercial assay kit.

**Results:**

We report here that HAdV-11 exerts its anti-ESCC effects via two pathways. Firstly, via the mitochondrial pathway, HAdV-11 promotes mitochondrial fission, thereby enhancing mitophagy, suppressing glutathione peroxidase 4 expression, and increasing lipid peroxidation, which ultimately induces ferroptosis; interestingly, HAdV-11-induced mitophagy attenuates ferroptosis in this context. Secondly, HAdV-11 triggers both mitophagy and ferroptosis through the mammalian target of rapamycin (mTOR) pathway. Our findings indicate that mitochondrial fission and mTOR signaling are closely associated with HAdV-11-induced cytotoxicity in ESCC cells.

**Conclusions:**

HAdV-11-induced ferroptosis in ESCC cells involves mitochondrial impairment and the mTOR pathway. These findings provide insights into HAdV-11’s oncolytic mechanisms and suggest strategies for enhancing its therapeutic efficacy in ESCC.

**Graphical Abstract:**

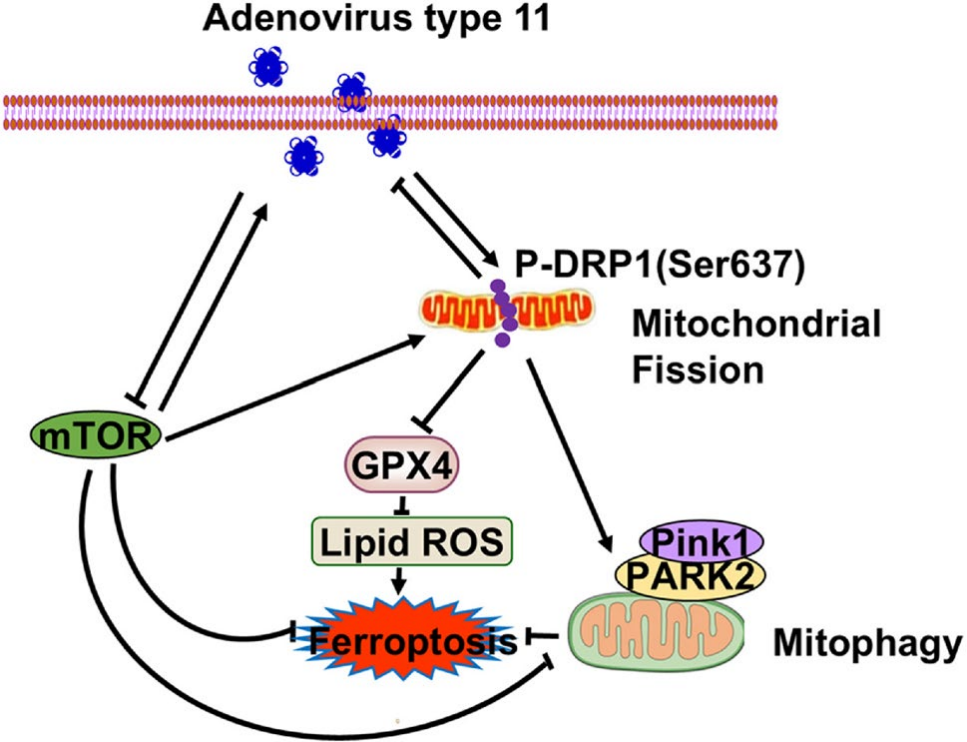

**Supplementary Information:**

The online version contains supplementary material available at 10.1186/s12885-026-15735-7.

## Background

Esophageal cancer is the sixth most common cause of cancer death worldwide, with approximately half of all early-stage esophageal cancer cases occurring in China. The five-year survival rate remains low, and novel treatment options are urgently required. Replication-selective oncolytic viruses (OVs) may be a promising new treatment avenue for many cancers. They act through multiple mechanisms, including direct tumor cell lysis and induction of robust anti-tumor immunity, to eliminate virus-uninfected tumor cells. Adenovirus type 5 (HAdV-5) is well characterized and easily produced, but its oncolytic use is limited by high neutralizing-antibody seroprevalence, receptor dependency and liver sequestration [1–3]. By contrast, we use.

A replication-competent Human adenovirus type 11p (HAdV-11p, subgroup B). No capsid modifications were introduced (no RGD insertion, fiber-knob swap, or hexon edit), thereby preserving the native subgroup-B tropism through CD46 and desmoglein-2 (DSG2) engagement rather than CAR. These receptors are broadly expressed in epithelial malignancies such as ESCC, supporting efficient tumor entry and reduced hepatic sequestration compared with Ad5-based vectors. Tumor selectivity was achieved by placing E1A under an hTERT-E1A chimeric promoter (5ETel), restricting replication to telomerase-positive cells while maintaining E1B integrity for optimal replication fitness. This dual selectivity—receptor-based and transcriptional—follows clinically validated Oncolytic Adenovirus (OAd) strategies balancing safety and potency [4–6]. Together, receptor-based and transcriptional selectivity provide a rational framework for developing HAdV-11–based oncolytic adenoviruses with improved safety and efficacy profiles.

We previously found that some proteins of Severe Acute Respiratory Syndrome Coronavirus 2 (SARS-CoV-2) and HAdV-11 are regulated by hijacking host mitochondria, and that mitochondrial-targeted drugs significantly repress virus protein production [7]. Mitochondria, essential for cellular energy production, undergo dynamic processes of fusion and fission to maintain functionality under cellular stress conditions [8]. Dysregulation of mitochondrial dynamics has been implicated in cancer progression, therapy resistance, and virus–host interactions. In particular, mitochondrial fission is initiated by the cytosolic GTPase dynamin-related protein 1 (DRP1), whose recruitment to the outer mitochondrial membrane is regulated by post-translational modifications, including phosphorylation at Ser637 [9, 10], Mitochondrial quality control is further maintained by mitophagy, a selective autophagic process that removes damaged or dysfunctional mitochondria and contributes to cellular homeostasis under stress conditions a selective autophagic process that removes damaged or dysfunctional mitochondria and contributes to cellular homeostasis under stress conditions [11]. Several oncogenic signaling pathways, including the phosphatidylinositol 3-kinase (PI3K)–AKT–mechanistic target of rapamycin (mTOR) axis, are known to regulate both mitochondrial dynamics and autophagy, and have also been reported to influence oncolytic virus replication and therapeutic responses [12]. However, how these mitochondrial processes intersect with subgroup B adenovirus infection in ESCC cells remains largely unexplored.

Ferroptosis, an iron-dependent form of regulated cell death, is characterized by the accumulation of lipid peroxides, shrunken mitochondria, and reduced mitochondrial cristae [13]. Glutathione peroxidase 4 (GPX4) and solute carrier family 7 member 11 are critical regulators of ferroptosis [14]. Studies have highlighted the role of ferroptosis in tumor cell death and growth inhibition [15], with oncolytic viruses inducing ferroptosis to exert anti-tumor effects [16]. Nevertheless, the relationship between mitochondrial quality control mechanisms and ferroptotic responses during oncolytic adenovirus infection has not been clearly defined.

Based on these considerations, this study was designed to investigate how HAdV-11 infection interacts with host mitochondrial dynamics, autophagy-related processes, and mTOR-associated signaling pathways in ESCC cells, and to explore how these interactions may influence virus-induced tumor cell death. A clearer understanding of these mechanisms may provide new insights into optimizing the therapeutic efficacy of HAdV-11 for esophageal cancer.

## Materials and methods

### Reagents

MitoTracker Green (MTG), MitoTracker Deep Red (MTR), BODIPY Lipid Probe, 5,5’,6,6’-Tetrachloro-1,1’,3,3’-Tetraethylbenzimi-dazolylcarbocyanine Iodide (JC-1) and MitoSOX™ were obtained from Invitrogen (Grand Island, NY, USA). FerroOrange was from Dojindo (Shanghai, China). Mitochondrial division inhibitor 1 (Mdivi-1), Ferrostatin-1, Erastin, MK2206, LY294002 and Temsirolimus (CCI-779) were purchased from MedChem Express (Shanghai, China). Monodansyl cadaverine (MDC) were purchased from Sigma Chemical (St Louis, MO, USA). Annexin V-FITC/PI Apoptosis Detection Kit was from Vazyme (Nanjing, China). Reduced glutathione (GSH) was obtained from Nanjing Jiancheng Bioengineering Institute (Nanjing, China). Primary antibodies against dynamin-related protein 1 (DRP1), voltage-dependent anion channel (VDAC), microtubule-associated protein 1 A/1B light chain 3 (LC3), PTEN-induced kinase 1 (PINK1), ubiquitin-protein ligase parkin (PARK2), and β-actin as well as horseradish peroxidase-conjugated secondary antibodies were obtained from Proteintech Group (Chicago, IL, USA). Primary antibody against mammalian target of rapamycin (mTOR) was from Cell Signaling Technology. Primary antibody against p-mTOR-S2448 was from ABclonal Technology. Primary antibodies against Phospho-DRP1 (S637) and GPX4 were purchased from Abcam (Cambridge, MA, USA). Formalin-fixed paraffin-embedded primary tumor and paracancer tissues were obtained from the first affiliated hospital of Zhengzhou University.

### Cell lines and adenoviruses

The human ESCC cell lines were obtained from the Japanese Collection of Research Bioresources Cell Bank (JCRB, Osaka, Japan). The HET-1 A cell line and human ESCC cells were grown in RPMI-1640 media supplemented with 10% fetal bovine serum (FBS), at 37 °C and 5% CO_2_. KYSE30 and KYSE410 cells were transfected with lentiviral vectors carrying the antisense sequence of DRP1 (LV-sh-DRP1), PARK2 (LV-sh-PARK2) or nonspecific scrambled RNA sequence (Scramble) using polybrene (Table 1). The small interfering RNAs targeting human mTOR (si mTOR), or negative control (control-siRNA) siRNAs were obtained from Sangon Biotech (Shanghai, China) (Table 2). KYSE30 and KYSE410 cells were transfected with siRNA using lipofectamine 2000 purchased from Thermo Fisher Scientific (Waltham, MA, USA). HAdV-11p was a kind gift from Daniel Stone and André Lieber (University of Washington, Seattle, WA). A type-B recombinant human adenoviral vector HAdV-11-5ETel-GFP and HAdV-11-5ETel with adenovirus Early region 1 A (E1A) enhancer and human telomerase reverse transcriptase (TERT) promoter was constructed according our previous study [17]. The plasmid encoding HAdV-11 fiber was constructed with the following primers, Fiber-F: TCAAGCTTACCGGTGCCACCATGACCAAGAGAGTCCGGCTCAGT, Fiber-R: ACGGATCCTCCGCCACCGCTACCTCCGCCACCGTCGTCTTCTCTGATGTAGTAAAAGGT.

**Table 1:** The sequences of ShRNA duplex

Gene	Sequence
Sense strand of DRP1	CCGGCTACTTTACTCCAACTTATTCTCGAGAATAAGTTGGAGTAAAGTAGCTTTTTG
Antisense strand of DRP1	AATTCAAAAACTACTTTACTCCAACTTATTCTCGAGAATAAGTTGGAGTAAAGTAGC
Sense strand of PARK2	CCGGGCGTGAACATAACTGAGGGCATCTCGAGATGCCCTCAGTTATGTTCACGTTTTTG
Antisense strand of PARK2	AATTCAAAAACGTGAACATAACTGAGGGCATCTCGAGATGCCCTCAGTTATGTTCACG

**Table 2:** The sequences of SiRNA duplex

Gene	Sequence
Sense strand of mTOR	5′-GAGCCUUGUUGAUCCUUAA/dT//dT/-3′
Antisense strand of mTOR	5′-UUAAGGAUCAACAAGGCUC/dT//dT/-3′
Sense strand of control	5′-UUCUCCGAACGUGUCACGU/dT//dT/-3′
Antisense strand of control	5′-ACGUGACACGUUCGGAGAA/dT//dT/-3′

### Cell viability assay and viral replication

Cell viability was measured using an MTS/PMS assay. Human ESCC cells (5 × 10^3^ cells/well) were seeded into 96-well plates overnight, infected with HAdV-11 at different multiplicities of infection (MOI), and incubated at 37 °C for 72 h. 20 µl MTS/PMS (Promega, Madison, WI, USA) were added to each well, which contained 200 µl of RPMI 1640. After incubation for 4 h, absorbance at 490 nm was measured using a microplate reader. The inhibition ratio was calculated as (absorbance value of untreated control cells-absorbance value of treated cells)/(absorbance value of untreated control cells)×100%.

Viral replication was evaluated in human ESCC cells. human ESCC cells were infected with HAdV-11 at an MOI of 2 plaque-forming units (PFU)/cell. Cells and supernatant were collected at 72 h post infection. The median tissue culture infective dose (TCID_50_) for each sample was calculated as previously described [18].

### Electron microscopy

For transmission electron microscopic analysis, cells were fixed with 3% glutaraldehyde for 2 days at room temperature, washed with PBS, post-fixed in 1% osmium tetroxide at room temperature for 1.5 h and washed with PBS. Thereafter, the samples were dehydrated in ethanol and acetone and then embedded. Ultrathin sections were isolated on nickel fitters, stained with uranyl acetate for 10 min and then lead citrate for 5 min, and finally examined with a transmission electron microscope (Hitachi-7650, Tokyo, Japan) at 60 kV. Mitochondrial length was measured in electron micrographs using ImageJ.

### Flow cytometry

Cell apoptosis was assessed using an annexin V-fluorescein isothiocyanate (FITC)/propidium iodide kit (Vazyme, Nanjing, China)) according to the supplier’s instructions. The levels of mitochondrial reactive oxygen species (ROS) were detected using the fluorescent probes MitoSOX™ Red. The cultures were stained with MitoSOX™ Red (0.005 mM) for 30 min at 37 °C. To measure mitochondrial mass, cells were incubated with MTG (20 nM) at 37 °C for 30 min. Mitochondrial membrane potential (ΔΨm) was measured by treatment with 10 µg/mL JC-1 at 37 °C for 20 min in the dark. In order to analyze autophagy, cells were incubated with MDC solution (0.05 mmol/L) at 37 °C for 30 min. C11 BODIPY 581/591 is a lipid-soluble ratiometric fluorescent indicator of lipid oxidation. Cells were stained with 2.5 µM C11-BODIPY581/591 for 30 min to detect lipid oxidation. Intracellular Fe^2+^ was measured using 1 µmol/L FerroOrang at 37 °C for 30 min. The evaluations above were analysed with a FACScan flow cytometer (Becton Dickinson, Franklin Lakes, NJ, USA), and the resulting files were analyzed using FlowJo software (version 10.9.0). Initial gating was performed to exclude cell debris using forward scatter (FSC-A) versus side scatter (SSC-A) plots. Single cells were identified by FSC-A versus FSC-H plots. Fluorescence-positive populations were gated based on negative and single-stained controls. For JC-1, the red (aggregated) and green (monomer) fluorescence signals were assessed in FL2 and FL1 channels respectively, and the red/green ratio was used to indicate ΔΨm. C11-BODIPY 581/591 oxidation was evaluated based on the shift from red to green fluorescence. Thresholds for each dye were set based on control populations to distinguish positive from negative cells.

### Measurements of reduced GSH

Reduced GSH was determined using the reduced GSH assay kit (Nanjing Jiancheng biotechnology, Nanjing, China) following the manufacturer’s protocol.

### Western blot analysis

Cells were lysed in immunoprecipitation assay (RIPA) lysis buffer with protease and phosphatase inhibitors (Roche). The BCA protein assay was used to quantify total protein. Cell lysates were resolved on SDS/PAGE gels and transferred to a nitrocellulose membrane. The membranes were incubated in 5% skim milk for 1 h at room temperature and then incubated with primary antibodies diluted in blocking buffer at 4 °C overnight. After three washes, membranes were incubated with appropriate secondary antibodies for 2 h at room temperature and subjected to chemiluminescence using Clarity Western ECL Substrate (Termo Pierce). To enhance detection specificity and efficiency, membranes were cut prior to hybridization with antibodies.

### Immunofluorescent staining

To label mitochondria and detect mitochondrial proteins, MTR was used. For staining with MTR, the cells were incubated with 100 nM of MTG for 10 min at 37 °C (Invitrogen). Cells were fixed in 4% paraformaldehyde for 20 min, permeabilized with 0.15% Triton X-100 for 10 min, blocked with 10% FBS at room temperature for 30 min, followed by overnight incubation with anti-PARK2 antibody (1:100) or anti-DRP1 antibody (1:100) at 4 °C. The coverslips were washed with PBS and incubated with fluorochrome-conjugated secondary antibodies (1:200 dilution) at room temperature for 2 h. The coverslips were washed with PBS three times to remove the excess antibody and stained with 4,6-diamino-2-phenyl indole (DAPI) for 10 min. In addition, the levels of mitochondrial ROS were detected using the fluorescent probes MitoSOX™ Red. The cultures were stained with MitoSOX™ Red (0.005 mM) for 30 min at 37 °C and then washed three with warm PBS. MitoSOX™ Red fluorescence was excited by green light at 510 nm and recorded at 580 nm. Images were captured using a confocal laser scanning microscopy (LSM880, Zeiss, Germany).

### Statistical analysis

All experiments were performed with a minimum of three independent biological replicates (*n* = 3), and the data are presented as means ± SD. One-way ANOVA was followed by Bonferroni’s post-hoc correction, and that for pairwise comparisons we used two-tailed Student’s t-tests with equal variance assumed. If the p-value was < 0.05, the difference was defined as statistically significant. All statistical analyses were performed using GraphPad Prism 10.

## Results

### HAdV-11 induced mitochondrial fission in ESCC cells

**Fig. 1:**
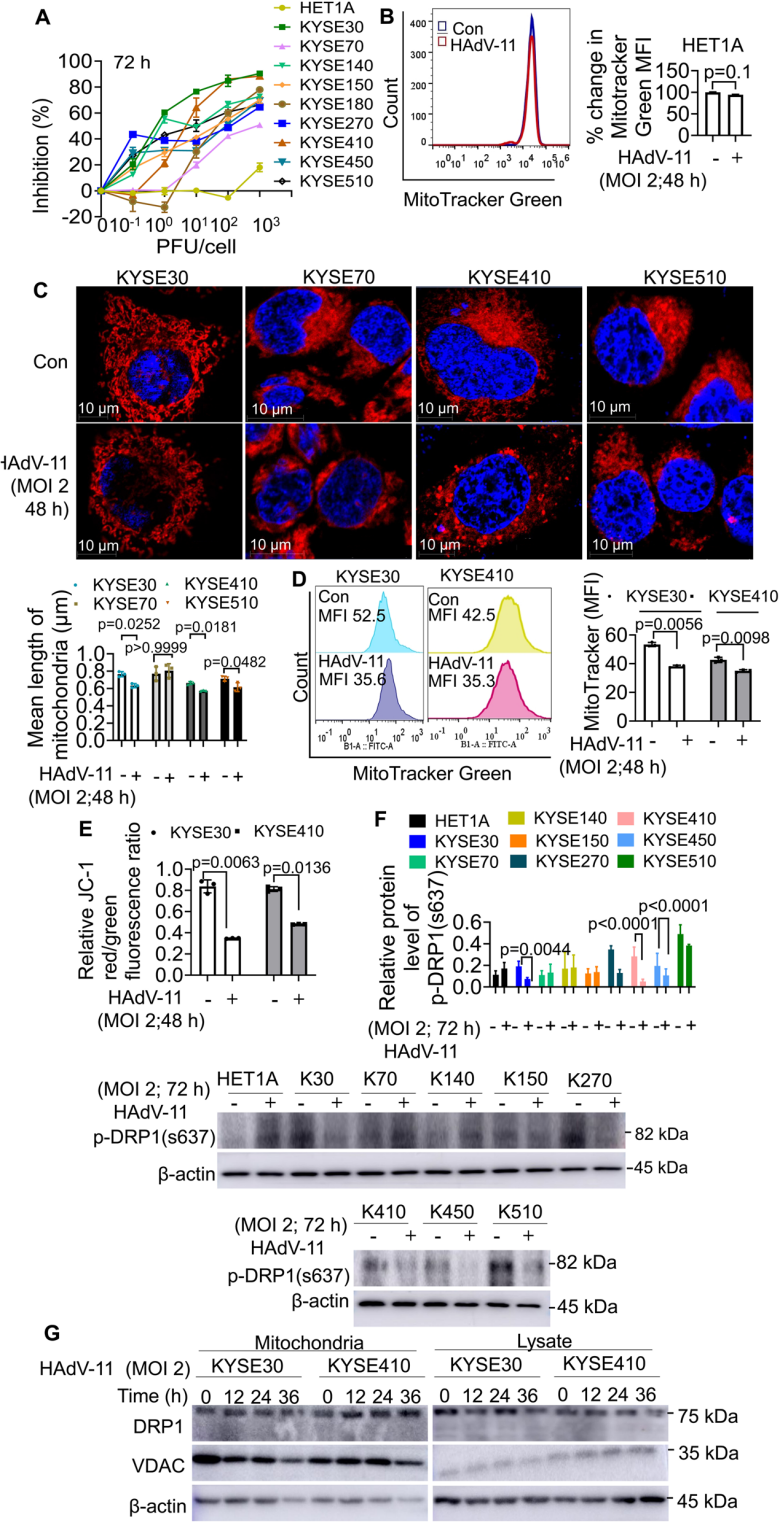
HAdV-11-induced mitochondrial fission in esophageal squamous cell carcinoma (ESCC) cells. (**A**) HET-1A cells and ESCC cells were treated with HAdV-11 for 72 h at different multiplicities of infection (MOI) of 0.1, 1, 10, 100 or 1000 plaque-forming units (PFU)/cell. Cell viability was evaluated using an MTS/PMS assay according to the manufacturers protocol. (**B** and **D**) HET-1A, KYSE30 and KYSE410 cells were infected with HAdV-11 for 48 h at an MOI of 2 PFU/cell, and treated with MitoTracker Green (MTG). The mitochondrial mass was determined using flow cytometry. (**C**) Cells were infected with HAdV-11 for 48 h at an MOI of 2 PFU/cell and stained with MitoTracker Deep Red (MTR) and then DAPI. The morphology of mitochondria was examined under the fluorescence microscopy. (**E**) Cells were infected with HAdV-11 for 48 h at an MOI of 2 PFU/cell, and treated with 5,5’,6,6’-Tetrachloro-1,1’,3,3’-Tetraethylbenzimi-dazolylcarbocyanine Iodide (JC-1). Mitochondrial membrane potential (ΔΨm) was determined using flow cytometry. (**F**) Cells were infected with HAdV-11 for 72 h at an MOI of 2 PFU/cell. Western blot analysis of dynamin-related protein 1 phosphorylated at serine 637 (p-DRP1(Ser637)). (**G**) Cells were infected with HAdV-11 for 72 h at an MOI of 2 PFU/cell. Western blot analysis of DRP1 expressions. β-actin and voltage-dependent anion channel (VDAC) were used as loading controls for the whole cell lysates and mitochondrial lysates respectively. Data are cumulative results from 3 experiments (**A**, **E**) or are representative of 3 independent experiments (**B**, **C**, **D**,** F**, **G**). Data in (**A**, **B**, **C**, **D**, **E** and **F**) are presented as means ± SD, two-tailed t-test (*n* = 3)

An MTS assay was performed to investigate the effect of HAdV-11 infection on ESCC cell proliferation. As shown in Fig. 1A and Supplementary Fig. 1A, ESCC cell proliferation was inhibited in a dose-dependent manner after infection, notably, a negative inhibition rate is an indicator of enhanced cell growth. We hypothesize that low MOI may moderately activate certain survival or growth-promoting pathways. An increase in p-mTOR (Ser2448) levels was observed in KYSE410 cells infected with HAdV-11 at a lower MOI, whereas a decrease was detected in KYSE30 cells (Supplementary Fig. 1B). Proliferation of a human normal squamous oesophageal cell line, HET-1A, was unaffected after infection. Four ESCC cell lines, KYSE30, KYSE410, KYSE510 and KYSE140, were more sensitive to HAdV-11 (1–10 pfu/cell). Mitochondrial impairment has been observed after oncolytic virus infectious [19]. In control cells, the MTG median fluorescence intensity (MFI) was approximately 100, whereas HAdV-11-treated cells exhibited an average MFI of ~ 94.23 (MD=-5.770 ± 2.862, 95% CI= -13.72 to 2.176), with no significant differences (Fig. 1B). HAdV-11 exhibited markedly higher replication efficiency in ESCC cells than in normal epithelial cells. Upon infection at MOI = 2, viral titers increased time-dependently in all lines but were substantially greater in KYSE30 and KYSE410 than in HET-1A (Supplementary Fig. 1C). At 72 h post infection, titers reached 58.22 ± 10.89 PFU/cell in KYSE30 (MD = 54.89, 95% CI 16.35–93.42, *p* = 0.025) and 193.27 ± 12.19 PFU/cell in KYSE410 (MD = 189.9, 95% CI 147.1–232.8, *p* = 0.003), compared with 3.34 ± 0.32 PFU/cell in HET-1A—representing ~ 17-fold and ~ 58-fold increases, respectively. Similar selectivity was evident at 24 h and 48 h timepoints. As HAdV-11 showed negligible effects on HET-1A cells, we focused on its impact in ESCC lines. We selected cell lines, KYSE30, KYSE70, KYSE410 and KYSE510, to determine whether HAdV-11 infection caused impairment of mitochondrial dynamics. MTR staining demonstrated that HAdV-11 decreased mitochondrial length in KYSE30, KYSE410 and KYSE510 cells with noticeable morphological changes from oval shape into the truncated and fragmented morphologies. HAdV-11 reduced mitochondrial length in KYSE30 cells (0.765 ± 0.017 to 0.632 ± 0.024 μm, MD = 0.1362, 95%CI = 0.04068 to 0.2316), KYSE410 (0.663 ± 0.015 to 0.574 ± 0.01 μm, MD = 0.09600, 95%CI = 0.03398 to 0.1580) and KYSE510 (0.713 ± 0.016 to 0.615 ± 0.029 μm, MD = 0.1037, 95%CI = 0.001188 to 0.2061), but not in KYSE70 (0.771 ± 0.047 to 0.807 ± 0.046 μm, MD =-0.03600, 95%CI = -0.7999 to 0.7279), indicating selective induction of mitochondrial fission (Fig. 1C). As shown in Fig. 1D, in KYSE30 cells, the MitoTracker MFI significantly decreased from 53.37 ± 0.83 to 38.20 ± 0.45 (MD = 15.17, 95%CI = 12.38 to 17.96) after HAdV-11 treatment. Similarly, in KYSE410 cells, the MFI decreased from 42.63 ± 1.04 to 34.97 ± 0.49 (MD = 8.200, 95%CI = 5.410 to 10.99) following HAdV-11 infection. Moreover, JC-1 staining showed that HAdV-11 disrupted ΔΨm in KYSE30 and KYSE410 cells. In KYSE30 cells, the ratio dramatically decreased from 0.8377 ± 0.0355 to 0.3472 ± 0.0017 (MD = 0.4906, 95%CI = 0.3196 to 0.6615) after HAdV-11 treatment. In KYSE410 cells, the JC-1 red/green fluorescence ratio declined from 0.8125 ± 0.0251 to 0.4805 ± 0.019 (MD = 0.3319, 95%CI = 0.1609 to 0.5029) following HAdV-11 infection. These findings quantitatively demonstrate that HAdV-11 markedly disrupts mitochondrial membrane integrity in ESCC cells (Fig. 1E). These results suggested that HAdV-11 selectively impaired mitochondria in ESCC cell lines without affecting normal esophageal epithelial cells. Western blotting results showed that HAdV-11 markedly decreased p‑DRP1(S637) protein expression in KYSE30 (0.1909 ± 0.0238 to 0.0736 ± 0.0068, MD = 0.1173, 95%CI = 0.001698 to 0.2330); KYSE270 (0.3466 ± 0.0171 to 0.1288 ± 0.0165, MD = 0.2178, 95%CI = 0.1021 to 0.3334) and KYSE410 (0.2824 ± 0.0432 to 0.0499 ± 0.0108, MD = 0.2325, 95%CI = 0.1168 to 0.3481), demonstrating inhibition of DRP1 S637 phosphorylation (Fig. 1F). HAdV-11 increased translocation of DRP1 to mitochondria at 12 to 36 h in KYSE30 and KYSE410 (Fig. 1G). These results revealed that HAdV-11 could increase mitochondrial fission through decreasing the expression of p-DRP1 (Ser637) protein in KYSE30 and KYSE410 cells. The differential expression of DRP1 in esophageal cancer cell lines could be attributed to a combination of genetic and environmental factors. HAdV-11 exerts a more significant impact on mitochondria in esophageal cancer cell lines KYSE30 and KYSE410; therefore, our subsequent studies will focus on these two cell lines.

### Inhibition of HAdV-11-induced mitochondrial fission attenuates anti-tumour efficacy of HAdV-11 in KYSE30 and KYSE410 cells

**Fig. 2:**
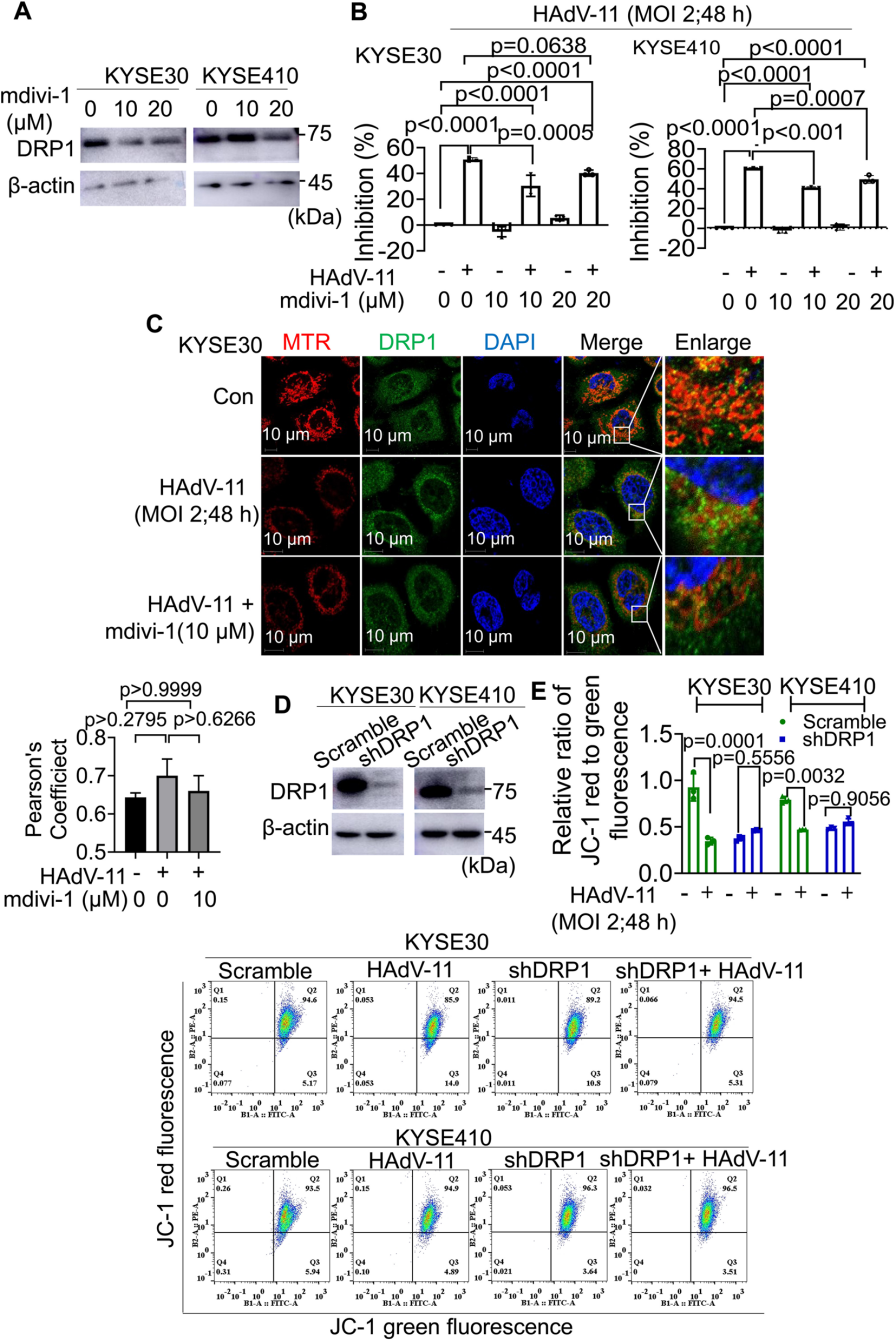
Inhibition of HAdV-11-induced mitochondrial fission attenuates anti-tumour efficacy of HAdV-11 in KYSE30 and KYSE410 cells. Cells were pretreated with Mdivi-1 for 2 h, and then treated with 2 PFU/cell of HAdV-11 for 48 h. (**A **and **D**) Western blots were used to detect the expression of DRP1. (**B**) Cell viability was measured using an MTS assay. (**C**) Confocal microscopy was used to detect the expression of DRP1. Cells were probed with MTR, primary anti-DRP1 antibody/secondary anti-mouse IgG Alexa Fluor R 488 conjugate and then DAPI. (**E**) ΔΨm was determined using JC‑1 dye by flow cytometry. Data are cumulative results from 3 experiments (**B**) or are representative of 3 independent experiments (**A**, **C**, **D**, **E**). Data are presented as mean ± SD (**B**, **E**). Differences analyzed using a one-way ANOVA with Bonferroni’s post hoc correction (**B**) or two-tailed t-test (E) (*n* = 3)

Mdivi-1, a selective DRP1 inhibitor, inhibits dynamin-1 GTPase activity that plays an essential role in mitochondria fission. Mdivi-1 significantly decreased the expression of DRP1 at 20 µM (Fig. 2A). Evaluation of the effect of Mdivi-1 in KYSE30 and KYSE410 cells using the MTS cytotoxicity assay showed that Mdivi-1 attenuated HAdV-11-induced cell growth inhibition (Fig. 2B). Pearson’s colocalization coefficients for DRP1 and mitochondrial MTR remained comparable across control (0.65 ± 0.02), HAdV-11 (0.69 ± 0.06, MD = -0.05667, 95% CI = -0.1501 to 0.03675), and HAdV-11 + Mdivi-1 (0.63 ± 0.02, MD = -0.01667, 95% CI = -0.1101 to 0.07675), with no significant differences (Fig. 2C). To investigate the role of DRP1 in regulating mitochondrial fission in ESCC cells, a DRP1-specific shRNA (shDRP1) was developed. Western blot analysis showed reduced DRP1 protein expression in KYSE30 and KYSE410 cells transduced with the DRP1-shRNA (Fig. 2D). In KYSE30 cells, the control group exhibited a ratio of 0.93 ± 0.085, which significantly decreased to 0.35 ± 0.022 (MD = 0.5791, 95% CI = 0.3974 to 0.7608) after HAdV-11 treatment. While, in KYSE30-shDRP1 cells the baseline ratio was 0.38 ± 0.014, increasing to 0.463 ± 0.0065 (MD = -0.08803, 95% CI = -0.2697 to 0.09368) in the HAdV-11-treated group, suggesting that the loss of ΔΨm upon HAdV-11 infection is mediated by DRP1. In KYSE410 cells, the control JC-1 ratio was 0.79 ± 0.020, which declined significantly to 0.47 ± 0.002 (MD = 0.3200, 95% CI = 0.1383 to 0.5017) upon HAdV-11 treatment. KYSE410-shDRP1 cells exhibited a baseline ratio of 0.48 ± 0.027; notably, when these cells were treated with HAdV-11, the ratio partially recovered to 0.55 ± 0.031 (MD = -0.06955, 95% CI = -0.2513 to 0.1122) (Fig. 2E). These data demonstrate that HAdV-11-induced mitochondrial depolarization is partly mediated by DRP1, and that its inhibition can partially reverse the loss of ΔΨm in ESCC cells. Taken together, inhibiting mitochondrial fission by targeting DRP1 could attenuated the anti-tumour efficacy of HAdV-11 in KYSE30 and KYSE410 cells.

### HAdV-11 induced mitophagy of KYSE30 and KYSE410 cells, and inhibiting mitochondrial fission attenuated mitophagy

**Fig. 3:**
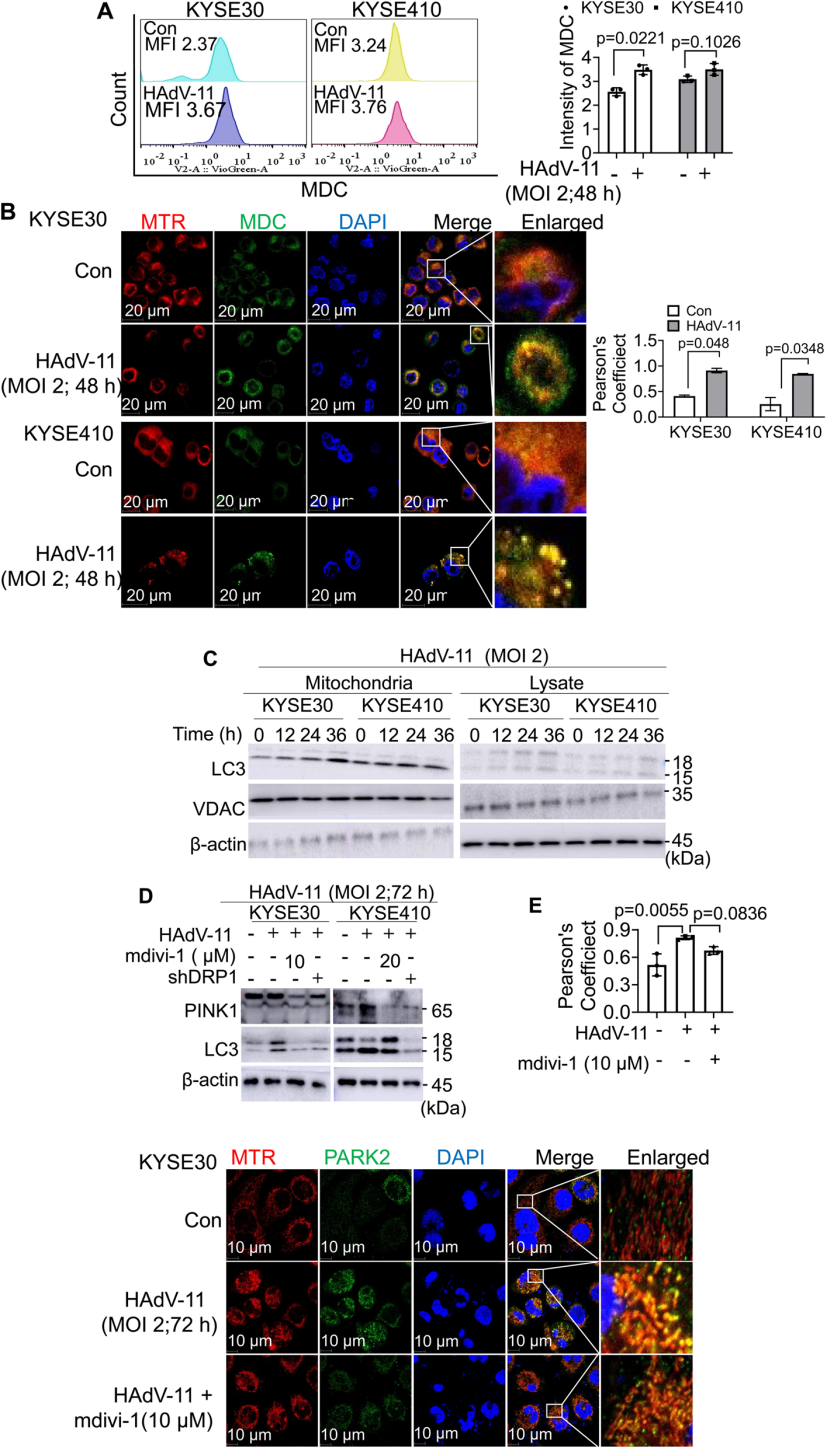
HAdV-11 induced mitophagy in KYSE30 and KYSE410 cells, while inhibition of mitochondrial fission attenuated mitophagy. (**A**) Indicated cells were infected with HAdV-11 for 48 h at an MOI of 2 PFU/cell. Relative quantification of positive MDC staining as detected by flow cytometric analysis. The positive ratios are presented in the histogram. (**B**) KYSE30/KYSE410 were infected (MOI = 2) and stained with monodansyl cadaverine (MDC) and MTR. Confocal microscopy was used to detect mitochondria colocalization. (**C**) Cells were infected with HAdV-11 for 72 h at an MOI of 2. Western blot analysis of microtubule-associated protein 1 A/1B light chain 3 (LC3) expression. β-actin and VDAC were used as loading controls for mitochondrial lysates. (**D**) KYSE30 cells and KYSE410 cells were treated with Mdivi-1 at concentrations of 10 µM and 20 µM, respectively, for 2 h, and then pretreated cells or pre-treated cells transduced with DRP1-shRNA were treated with 2 PFU/cell of HAdV-11 for 72 h, and Western blot analysis of LC3 and PTEN-induced kinase 1 (PINK1) expressions. (**E**) KYSE30 cells were infected with HAdV-11 for 72 h at an MOI of 2. Confocal microscopy was used to detect the expressions of E3 ubiquitin-protein ligase parkin (PARK2). Cells were probed with MTR, primary anti-PARK2 antibody/secondary anti-mouse IgG Alexa Fluor R 488 conjugate and then DAPI. Data are representative of 3 independent experiments (**A**, **B**, **C**, **D**, **E**). Data are presented as mean ± SD (**A**, **B**, **D**, **E**). Differences analyzed using a one-way ANOVA with Bonferroni’s post hoc correction (**D**, **E**) or two-tailed t-test (**A**, **B**) (*n* = 3)

Increased mitochondrial fission promotes mitophagy, an autophagic response that specifically targets damaged mitochondria to remove them [20]. Autophagy was also analyzed by flow cytometry by using monodansylcadaverine (MDC) staining. HAdV-11 infection of esophageal squamous cell carcinoma cell lines KYSE30 resulted in significantly enhanced MDC fluorescence (Fig. 3A). We investigated the temporal dynamics of MDC colocalization with mitochondria (labeled by MTR) to assess autophagic activity, with a focus on mitophagy. Following infection of KYSE30 and KYSE410 cells with HAdV-11 at a MOI of 2 for 48 h, a marked increase in MDC fluorescence intensity was observed, accompanied by significant colocalization with mitochondrial signals (Pearson’s coefficient > 0.8) (Fig. 3B), confirming the induction of mitophagy. We found that HAdV-11 treatment induced time-dependent accumulation of LC3-II in the mitochondrial fraction in a time dependent manner in KYSE30 and KYSE410 cells (Fig. 3C). Mdivi-1 or shRNA targeting DRP1 were then used to interfere with mitochondrial fission in order to determine whether mitochondrial fission played a critical role in HAdV-11-induced autophagy and mitophagy. We found that, in KYSE30 cells, HAdV-11 slightly decreased PINK1 expression and increased LC3 expression. Mdivi‑1 further decreased protein level of PINK1 and LC3, while shDRP1 had no significant effect. In KYSE410, HAdV-11 increased PINK1 and LC3 expressions; both Mdivi‑1 and shDRP1 partially reversed these changes (Fig. 3D and Supplementary Fig. 2). Immunofluorescence microscopy revealed increased colocalization of PARK2 with mitochondria in HAdV-11–treated cells (Pearson’s coefficient: control, 0.517 ± 0.120; HAdV-11, 0.813 ± 0.021, MD = -0.2967, 95% = -0.4770 to -0.1164), whereas this effect was attenuated by Mdivi-1 co-treatment (HAdV-11 + Mdivi-1, 0.673 ± 0.040, MD = -0.1567, 95% = -0.3370 to 0.02364) with no significant difference compared to control (Fig. 3E). Our results indicate that HAdV-11 induces mitophagy in both KYSE30 and KYSE410 cells, and that inhibition of mitochondrial fission significantly mitigates this effect.

### HAdV-11 induced ferroptosis of KYSE30 and KYSE410 cells

**Fig. 4:**
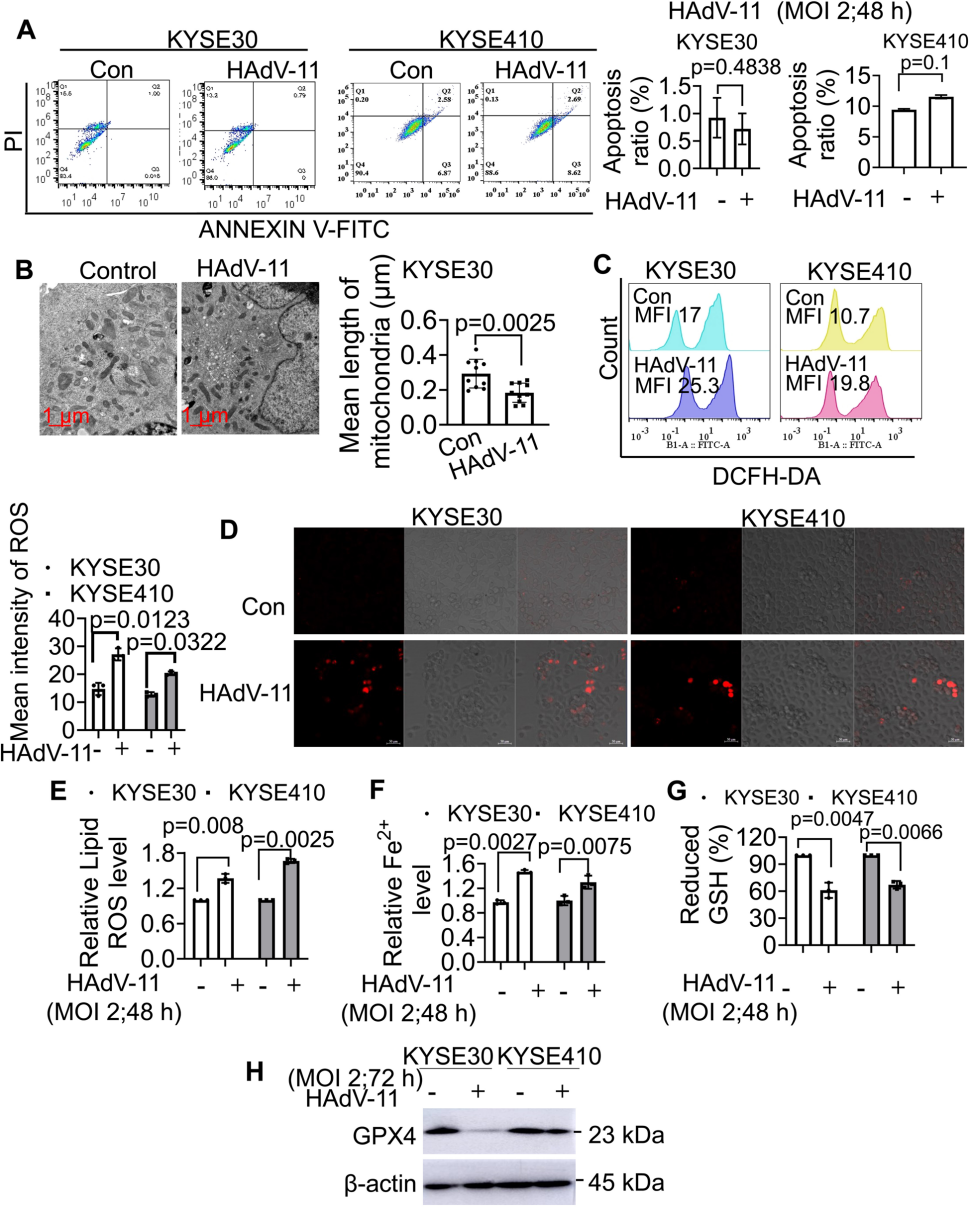
HAdV-11 induced ferroptosis of KYSE30 and KYSE410 cells. Indicated cells were treated with HAdV-11 for 48 h at an MOI of 2 PFU/cell. (**A**) Flow cytometric analysis of apoptotic cell ratios. Q2 + Q3 cells were defined as apoptosis. (**B**) The morphology of mitochondria was examined under the electron microscope (Scal bar = 2 μm). (**C**) Flow cytometric analysis of reactive oxygen species (ROS) levels after DCFH-DA staining. The positive ratios are presented in the histogram. (**D**) The cells were stained with MitoSOX™, and observed using fluorescence microscopy and phase contrast microscopy. (**E** and **F**) Cells were stained with C11-BODIPY or FerroOrange, and lipid peroxides or Fe^2+^ were determined by flow cytometry. (**G**) The reduced GSH level in KYSE30 cells was determined using reduced GSH assay kit. (**H**) Cells were treated with HAdV-11 for 72 h at an MOI of 2 PFU/cell, and Western blot analysis of glutathione peroxidase 4 (GPX4) expression. Data are cumulative results from 3 experiments (**E**, **F**, **G**) or are representative of 3 independent experiments (**A**, **B**, **C**, **D**, **H**). Data are presented as mean ± SD (**A**, **B**, **C**, **E**, **F**, **G**). Differences analyzed using two-tailed t-test (**A**, **B**, **C**, **E**, **F**, **G**) (*n* = 3)

Apoptosis and ferroptosis are forms of programmed cell death that activate genes and molecules inside the cell. We found that HAdV-11 infection did not significantly induce apoptosis in either KYSE30 or KYSE410 cells. The apoptotic rate in KYSE30 cells was 0.72 ± 0.29% for Ad11-treated cells compared to 0.92 ± 0.35% for controls (MD = -0.20, 95% CI = -0.9396 to 0.5313). Similarly, in KYSE410 cells, the rates were 11.53 ± 0.29% versus 9.41 ± 0.15% in controls (MD = 2.12, 95% CI = 1.770 to 2.610). Oncolytic Vaccinia virus combined with Erastin, a ferroptosis activator, promoted antitumoral efficacy after infection [21]. Several studies have shown that the mitochondria can act as initiators or amplifiers of ferroptosis [22], we were interested to determine how effectively HAdV-11 could induce ferroptosis in ESCC cell lines. Using electron microscopy, we observed that uninfected KYSE30 cells had large, tubular, densely packed mitochondria in the cytoplasm, but the HAdV-11-infected KYSE30 cells had more mitochondria with small, fragmented morphology. The control group exhibited an average mitochondrial length of 0.293 ± 0.026 μm, whereas HAdV-11-treated cells showed a significantly lower mean length of 0.183 ± 0.018 μm (MD = -0.1096, 95% CI = -0.1752 to -0.04410). This represents an approximate 37% decrease in mitochondrial length following HAdV-11 infection, representing one of main characteristics of ferroptosis (Fig. 4B). HAdV-11 infection led to increased cellular ROS levels (as measured by DCFH-DA) and mitochondrial ROS levels (as measured by MitoSOX™) in both KYSE30 and KYSE410 cells (Fig. 4C-D). HAdV-11 infection also resulted in the accumulation of lipid peroxides, as quantified by C11-BODIPY fluorescence intensity, in KYSE30 and KYSE410 cells (KYSE30: control, 1.000; HAdV-11, 1.369 ± 0.076, MD = -0.3687, 95% CI = -0.5142 to -0.2232; KYSE410: control, 1.000; HAdV-11, 1.665 ± 0.037, MD = -0.6651, 95% CI = -0.8105 to -0.5196) (Fig. 4E). We then examined the iron levels in KYSE30 cells and found that ferrous iron (Fe^2+^) accumulated in KYSE30 and KYSE410 cells in response to HAdV-11 infection (Fig. 4F). HAdV-11 treatment also reduced intracellular GSH levels in KYSE30 and KYSE410 cells (KYSE30: control, 100; HAdV-11, 61.0 ± 8.5, MD = 38.98, 95% CI = 27.26 to 50.70; KYSE410: control, 100; HAdV-11, 67.2 ± 4.0, MD = 32.81, 95% CI = 21.08 to 44.53), as shown in Fig. 4G. Moreover, lower levels of GPX4 protein were produced in HAdV-11-infected KYSE30 and KYSE410 cells (Fig. 4H). The results indicated that HAdV-11 triggered ferroptosis of esophageal cancer cells.

### Mitochondrial fragmentation exacerbates HAdV-11-induced ferroptosis in KYSE30 and KYSE410 cells, while mitophagy attenuates it

**Fig. 5:**
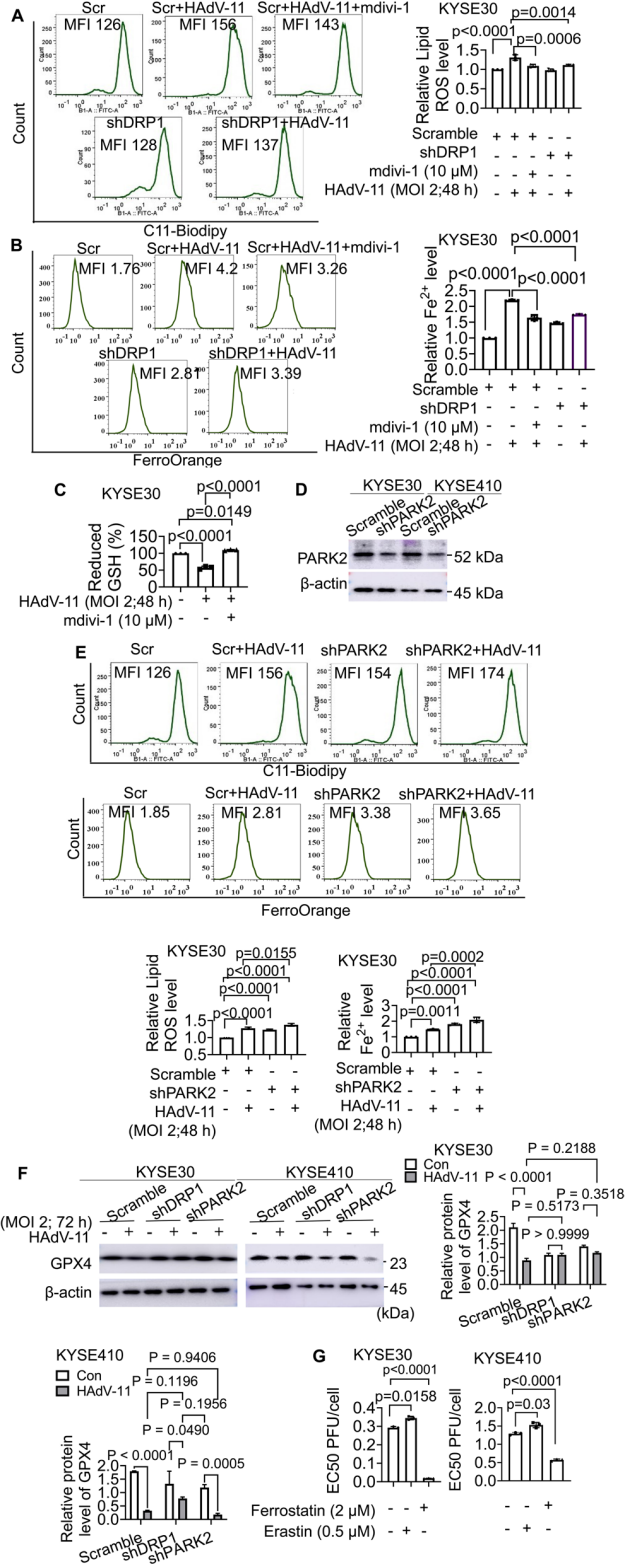
Mitochondrial fragmentation exacerbates ferroptosis caused by HAdV-11 in KYSE30 and KYSE410 cells, while mitophagy attenuates it. Cells were treated with HAdV-11 for 48 h at an MOI of 2 PFU/cell. (**A**, **B** and **E**) Cells were stained with C11-BODIPY or FerroOrange, and lipid peroxides or Fe^2+^ were determined by flow cytometry. (**C**) The reduced GSH level in KYSE30 cells was determined using reduced GSH assay kit according to the manufacturers protocol. (**D** and **F**) Protein expression of PARK2 and GPX4 was detected by Western blots in scrambled-shRNA transduced cells and PARK2-shRNA transduced cell lines. (**G**) Cells were pretreated with Ferrostatin-1 (Fer-1) or Erastin for 2 h, and infected with 2 PFU/cell of HAdV-11 for 72 h. The half maximal effective concentration (EC50) for each sample was calculated. Data are cumulative results from 3 experiments (**C**, **G**) or are representative of 3 independent experiments (**A**, **B**, **D**,** E**, **F**). Data are presented as mean ± SD (**A**,** B**, **C**, **E**, **F**, **G**). Differences analyzed using a one-way ANOVA with Bonferroni’s post hoc correction (**A**, **B**, **C**, **E**, **F**, **G**) (*n* = 3)

Ferroptosis is a type of autophagy-dependent cell death [23]. Our study investigates the roles of mitochondrial fission and mitophagy in modulating ferroptosis induced by HAdV-11 in ESCC cells. We employed specific interventions—Mdivi-1, shDRP1, and shPARK2—to assess their impact on ferroptosis. HAdV-11 infection significantly elevated lipid ROS and intracellular Fe²⁺ levels in KYSE30 cells compared with the Scramble control, indicating ferroptotic activation. Quantitatively, lipid ROS increased from 1.000 ± 0.000 to 1.308 ± 0.072 (MD = − 0.31, 95% CI = − 0.43 to − 0.19), while Fe²⁺ rose from 1.000 ± 0.000 to 2.193 ± 0.019 (MD = − 1.19, 95% CI = − 1.32 to − 1.07). Both Mdivi-1 treatment and DRP1 silencing effectively reversed these changes, reducing lipid ROS to 1.091 ± 0.040 and 1.111 ± 0.021, and Fe²⁺ to 1.642 ± 0.086 and 1.741 ± 0.013, respectively. These findings indicate that DRP1-mediated mitochondrial fission is required for HAdV-11–induced oxidative and iron accumulation, supporting a mechanistic link between mitochondrial dynamics and ferroptotic cell death (Fig. 5A-B). Moreover, co-treatment with Mdivi-1 elevated GSH levels (Fig. 5C), suggesting a restoration of redox balance. These findings indicate DRP1-mediated mitochondrial fission exacerbates HAdV-11-induced ferroptosis in ESCC cells.

To assess the role of mitophagy, we knocked down PARK2 using shRNA, which led to decreased PARK2 protein levels in KYSE30 and KYSE410 cells (Fig. 5D). In HAdV-11-infected cells, PARK2 knockdown further increased lipid ROS generation and Fe²⁺ accumulation compared to HAdV-11 infection alone (Fig. 5E), indicating that mitophagy serves a protective function against ferroptosis. In KYSE30 cells, HAdV-11 infection decreased GPX4 protein levels from 2.106 ± 0.258 to 0.885 ± 0.135 (MD = 1.221, 95% CI = 0.4817 to 1.960), whereas DRP1 knockdown maintained GPX4 protein level at 1.085 ± 0.116 (MD = 1.020, 95% CI = 0.2813 to 1.759) and 1.088 ± 0.102 (MD = 1.018, 95% CI = 0.2786 to 1.757) in control and HAdV-11-treated cells, indicating a protective effect. Conversely, PARK2 knockdown increased baseline GPX4 protein to 1.405 ± 0.076 but further decreased it to 1.164 ± 0.068 (MD = 0.9412, 95% CI = 0.2022 to 1.680) with HAdV-11 infection. In KYSE410 cells, similar trends were observed: HAdV-11 decreased the expression level of GPX4 from 1.793 ± 0.013 to 0.324 ± 0.015; DRP1 knockdown partially restored GPX4 levels to 0.781 ± 0.052, while PARK2 knockdown exacerbated its loss to 0.179 ± 0.048 (Fig. 5F). These results suggest that DRP-mediated fission facilitates GPX4 downregulation, promoting ferroptosis, whereas PARK2-dependent mitophagy helps maintain GPX4 expression, mitigating ferroptosis. Finally, we assessed how ferroptosis influences HAdV-11 replication. Treatment with Ferrostatin-1, a selective ferroptosis inhibitor, enhanced HAdV-11 replication, while the ferroptosis inducer Erastin inhibited HAdV-11 replication in KYSE30 and KYSE410 cells (Fig. 5G). Our data demonstrate that DRP1-mediated mitochondrial fission exacerbates, while PARK2-dependent mitophagy mitigates, HAdV-11-induced ferroptosis in ESCC cells.

To define the temporal window of mitophagy’s protective effect, we supplemented our experiments by examining MDC co-localization with mitochondria using confocal microscopy imaging in HAdV-11-infected cells at MOI = 2 on Day 3 and Day 5. In KYSE30 cells, MDC co-localization with mitochondria showed a slight increase on Day 3 post-infection, followed by a significant decrease on Day 5; KYSE410 cells exhibited a marked increase in MDC-mitochondrial co-localization on Day 3 that subsequently declined significantly by Day 5 (Supplementary Fig. 3A). On Day 5 at MOI = 2, we further investigated the role of mitophagy in ferroptosis regulation. Our results demonstrated that HAdV-11 infection significantly reduced GPX4 protein levels in both KYSE30 and KYSE410 cells. However, PARK2 knockdown had no significant impact on GPX4 expression levels (Supplementary Fig. 3B). These findings suggest that mitophagy initially provides protective effects against ferroptosis in esophageal squamous carcinoma cells during early-stage HAdV-11 infection, but this protective mechanism appears to be lost in later stage. The potential involvement of alternative autophagy pathways in later stages warrants further investigation.

### PI3K/Akt/mTOR pathway is essential in HAdV-11-treated ESCC cells

**Fig. 6:**
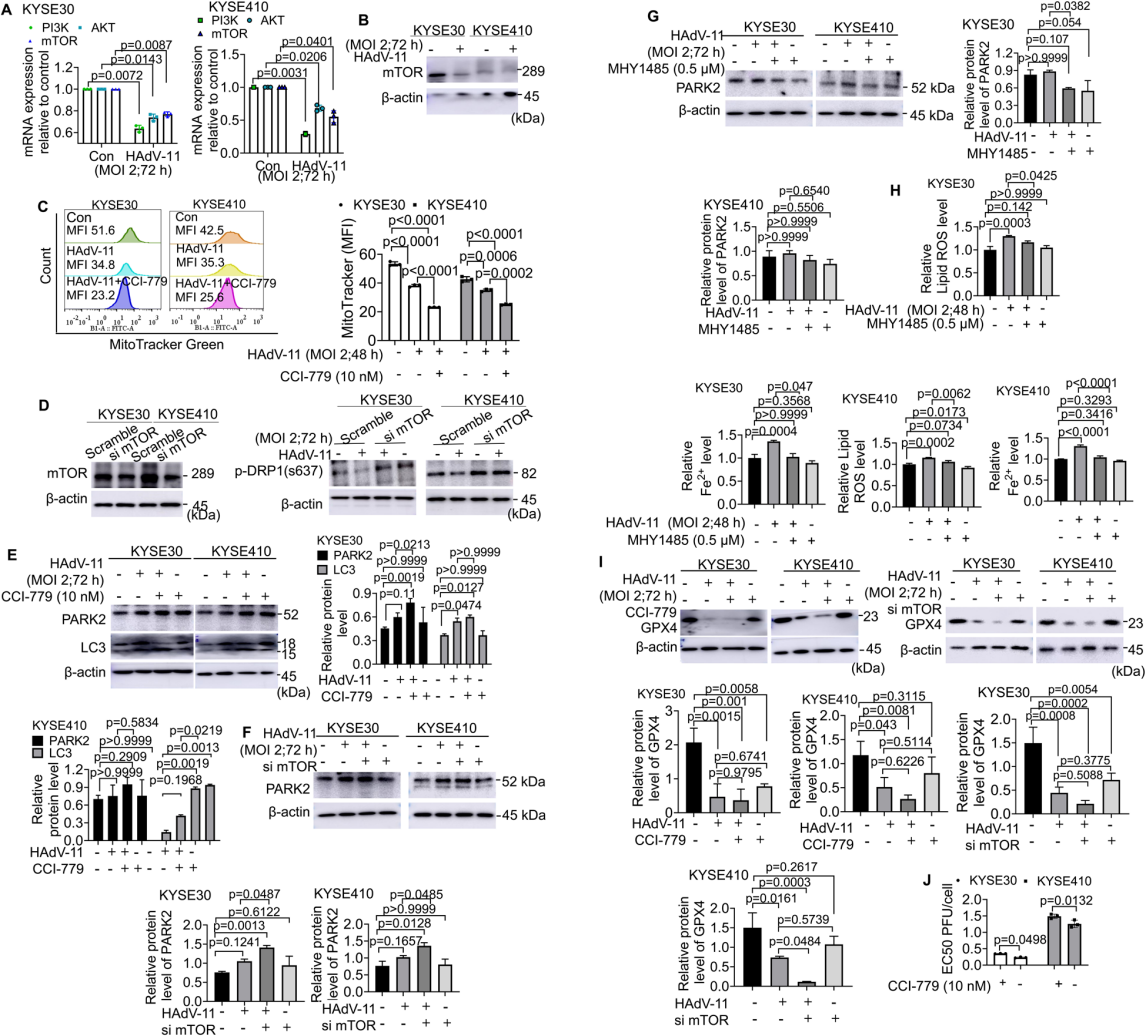
The PI3K/Akt/mTOR pathway is essential for HAdV-11 replication in ESCC cell lines. (**A**) Cells were treated with HAdV-11 for 72 h at an MOI of 2 PFU/cell, the mRNA expression levels were determined using quantitative polymerase chain reaction (qPCR). (**B**, **D**, **E**, **F**, **H** and **I**) Cells were treated with HAdV-11 for 72 h at an MOI of 2 PFU/cell, and Western blot analysis of mTOR, p-DRP1, PARK2, LC3 and GPX4 expression. (**C**) Cells were pretreated with Temsirolimus (CCI-779) (10 nM) for 2 h, and then infected with HAdV-11 at an MOI of 2 for 48 h. The mitochondrial mass was estimated by MTG staining. (**H**) After pretreatment with 0.5 µM mTOR agonist (MHY1485) for 2 h, cells were infected with HAdV-11 at a MOI of 2 for 48 h. Subsequently, the cells were stained with either C11-BODIPY or Fe^2+^ and quantified using flow cytometry. (**J**) Cells were pretreated with CCI-779 for 2 h, and then infected with HAdV-11 at an MOI of 2 for 72 h. Infectious virus production was assessed by titration on JH293 cells and the titer as PFU/cell calculated. The half maximal effective concentration (EC50) for each sample was calculated. Data are cumulative results from 3 experiments (**A**,** J**) or are representative of 3 independent experiments (**B**, **C**, **D**, **E**, **F**, **G**, **H**,** I**). Data are presented as mean ± SD (**A**, **C**, **E**, **F**, **G**, **H**, **I**, **J**). Differences analyzed using a one-way ANOVA with Bonferroni’s post hoc correction (**C**, **E**, **F**, **G**, **H**,** I**) or two-tailed t-test (**A**, **J**) (*n* = 3)

To determine the upstream intracellular pathways involved in translation regulation after HAdV-11 infection, the PI3K/Akt/mTOR pathway was first examined [24]. There was a significant decrease in the expression of PI3K, AKT, and mTOR mRNA in HAdV-11–treated KYSE30 and KYSE410 cells (Fig. 6A). Specifically, in KYSE30 cells, relative mRNA levels were reduced from 1.000 ± 0.000 to 0.636 ± 0.031 (MD = 0.3641, 95% CI = 0.2273 to 0.5009) for PI3K, from 1.000 ± 0.000 to 0.739 ± 0.031 (MD = 0.2614, 95% CI = 0.1227 to 0.4000) for AKT, and from 1.000 ± 0.000 to 0.769 ± 0.022 (MD = 0.2311, 95% CI = 0.1358 to 0.3265) for mTOR. Similarly, in KYSE410 cells, PI3K decreased from 1.000 ± 0.000 to 0.290 ± 0.040 (MD = 0.7096, 95% CI = 0.5339 to 0.8853), AKT from 1.000 ± 0.000 to 0.683 ± 0.046 (MD = 0.3172, 95% CI = 0.1150 to 0.5193), and mTOR from 1.000 ± 0.000 to 0.553 ± 0.091 (MD = 0.4475, 95% CI = 0.04754 to 0.8474). Treatment with HAdV-11 resulted in reduced mTOR and p-mTOR (Ser 2448) protein expression in KYSE30 and KYSE410 cells, suggesting the mTOR signaling pathway is inhibited during HAdV-11 infection (Fig. 6B and Supplementary Fig. 3C). As HAdV-11 infection decreased the expression of mTOR both in KYSE30 cells and KYSE410 cells, we used CCI-779, an inhibitor of mTOR, to test whether HAdV-11 inhibited ESCC cell proliferation through the mTOR signaling pathway. Compared with HAdV-11-infected cells, CCI-779 treatment together with HAdV-11 infection significantly decreased mitochondrial mass in both cell lines (Fig. 6C). We used both genetic knockdown of mTOR via siRNA and pharmacological modulation with an mTOR agonist (MHY1485) to further validate the role of the mTOR pathway in HAdV-11-induced cell death in ESCC cells. Our Western blot analysis revealed that HAdV-11 reduced the expression of p-DRP1 (Ser637); importantly, this effect was reversed by mTOR siRNA (Fig. 6D), thereby reinforcing the regulatory role of mTOR signaling in mitochondrial dynamics. Furthermore, we investigated the impact of mTOR on HAdV-11-induced mitophagy and ferroptosis. HAdV-11 increased the expression of mitophagy-related proteins LC3, and both CCI-779 and mTOR siRNA further augmented HAdV-11-induced PARK2 and LC3 expression in KYSE30 and KYSE410 cells (Fig. 6E and F), whereas MHY1485 antagonized the HAdV-11-induced upregulation of PARK2 in KYSE30 (Fig. 6G). Moreover, HAdV-11 elevated lipid ROS and Fe²⁺ levels in KYSE30 and KYSE410 cells; however, treatment with MHY1485 reversed these effects (Fig. 6H). The Western blot showed that in KYSE30 cells, GPX4 protein levels are significantly reduced in the HAdV-11, HAdV-11 + CCI-779, and HAdV-11 + mTOR siRNA groups compared with controls, indicating that HAdV-11 treatment robustly downregulates GPX4. The combination treatments (HAdV-11 + CCI-779 or HAdV-11 + mTOR siRNA) exhibited a further numerical decline in GPX4 relative to HAdV-11 treatment alone, but these additional reductions did not reach statistical significance. A similar pattern was observed for KYSE410 cells, with a clearer trend of further GPX4 reduction in the HAdV-11 + mTOR siRNA group compared with HAdV-11 alone (Fig. 6I). In addition, CCI-779 treatment decreased the replication efficiency of HAdV-11 in KYSE30 and KYSE410 cells (Fig. 6J). The EC₅₀ values increased from 0.232 ± 0.016 to 0.351 ± 0.006 (MD = 0.1183, 95% CI = 0.0002686 to 0.2364) in KYSE30 cells and from 1.257 ± 0.108 to 1.490 ± 0.065 (MD = 0.2333, 95% CI = 0.1153 to 0.3514) in KYSE410 cells, indicating reduced viral propagation upon mTOR inhibition. Collectively, these findings confirm that mTOR signaling plays a critical role in modulating mitochondrial fission, mitophagy, and ferroptosis in HAdV-11-treated ESCC cells.

Taken together, this study showed that HAdV-11 exerts its anti-ESCC effects through two pathways. Firstly, via the mitochondrial pathway, HAdV-11 promotes mitochondrial fission, leading to mitophagy, suppressing GPX4 expression, and promoting lipid peroxidation, ultimately inducing ferroptosis. Mitophagy inhibited ferroptosis. Secondly, HAdV-11 triggers mitophagy and ferroptosis through the mTOR pathway. Both excessive mitochondrial fission and low mTOR expression in KYSE30 and KYSE410 cells suppressed HAdV-11 replication.

## Discussion

There is increasing evidence that the host responds rapidly to viral infection by orchestrating the mitochondrial compartment to regulate the viral infection [25, 26]. Conversely, viruses are able to hijack the mitochondria to promote their replication and survival. Newcastle disease virus (NDV) has been shown to shift the balance of mitochondrial dynamics and activate mitophagy [19] and also can induce ferritinophagy by the promotion of ferroptosis [16]. Our findings demonstrate that oncolytic adenovirus HAdV-11 induces mitochondrial fission, leading to mitophagy in ESCC cells. Excessive mitochondrial fission induced ferroptosis in ESCC cells, while mitophagy alleviated this effect. ESCC cell mitochondria respond to HAdV-11 stimulation by increasing fission, resulting in more damaged mitochondria and promoting mitophagy to clear these damaged organelles. In this process, we can enhance the anti-tumor effect of HAdV-11 by promoting mitochondrial fission or inhibiting mitophagy.

Mitochondrial morphology is maintained by a balance between mitochondrial fission and fusion. Viruses frequently hijack mitochondrial dynamics to optimize replication. Hepatitis C virus activates CDK1 to phosphorylate DRP1 at Ser616, promoting fission and mitophagy [27]. Conversely, DRP1 Ser637 is dephosphorylated by calcineurin to permit mitochondrial recruitment and fission [28]. Though we have not yet identified the precise kinase/phosphatase altered by HAdV-11, the virus may modulate PKA or calcineurin activity through its early proteins, as has been described for other adenoviral serotypes [29, 30]. In our study, HAdV-11 induces mitochondrial fission, resulting in mitophagy in KYSE30 and KYSE410 ESCC cells. We therefore propose the following working model: HAdV-11 infection decreases Ser637 phosphorylation by either suppressing PKA activity or activating calcineurin, facilitating DRP1 recruitment and mitochondrial fission. To validate this model in future studies, we will quantify PKA and calcineurin activity in HAdV-11-infected ESCC cells, evaluate their regulatory roles on DRP1 Ser637 phosphorylation, mitochondrial fission, and ferroptosis using pharmacological modulators, and investigate whether HAdV-11 early proteins directly modulate these signaling pathways.Apoptosis and ferroptosis are classical forms of cell death. Kan et al. reported that NDV induces ferroptosis by suppressing the cystine/glutamate antiporter-GSH-GPX4 axis [16]. Ferroptosis inducer Erastin promotes antitumoral immunity and efficacy of oncolytic Vaccinia virus [21]. Here, our data indicates that HAdV-11 triggers ferroptosis of ESCC cells. Ferroptosis induced by HAdV-11 was exacerbated by mitochondrial fission, and inhibition of DRP1 attenuated it. This underscores that enhanced mitochondrial fission directly contributes to ferroptosis. Although we did not directly assess immunogenic cell death markers in this study, prior reports have shown that oncolytic adenoviruses can trigger these DAMPs and elicit immunogenic forms of tumor cell death [31, 32]. Future work will directly measure these parameters to confirm whether HAdV-11-mediated ferroptosis also contributes to antitumor immune priming. Mitophagy’s impact on ferroptosis is inherently context-dependent. In situations of mild stress or early iron overload, mitophagy can sequester excess iron within mitophagosomes, thereby decreasing ROS generation and reducing lipid peroxidation [33]. Conversely, studies in A549 and PANC-1 cells have demonstrated that extensive mitophagy exacerbated ferroptosis [34]. In our current study, ESCC cells infected with HAdV-11 at an MOI of 2 PFU/cell for 72 h exhibited a protective phenotype. We hypothesize that at this infection stage, ESCC cells benefit from moderate mitophagic activity that sequesters iron, whereas prolonged or higher-level viral replication may overwhelm these protective mechanisms, leading to a shift in the balance toward ferroptosis.

The PI3K/Akt/mTOR signaling axis critically regulates oncolytic virus-mediated antitumor immunity potentiation [35]. Sindbis virus replication suppresses Akt/mTOR pathway late during infection in HEK293T cells [36]. Oncolytic NDV/FMW triggers autophagy in A549/PTX cells via the inhibition of PI3K/Akt/mTOR/p70S6K pathway [37]. In this study, the mTOR signaling pathway exerts a multifaceted regulatory role in this process. Firstly, inhibition of mTOR directly suppresses the replication of HAdV-11. Secondly, mTOR inhibitors attenuate excessive mitochondrial fission, which on one hand directly mitigates ferroptosis, and on the other hand suppresses mitophagy, thereby reducing the protective effects on ESCC cells and enhancing ferroptosis in these cells. Thirdly, mTOR inhibitors directly induce ferroptosis in ESCC cells. Together, these findings illustrate that mTOR functions as a critical rheostat that balances mitochondrial fission and mitophagy.

We found that CCI-779 treatment alone resulted in a reduction of GPX4 protein levels. when CCI-779 was administered in combination with HAdV-11 infection, the reduction in GPX4 did not reach statistical significance. Mechanistically, mTORC1 sustains GPX4 levels by promoting cap-dependent translation via phosphorylation of p70S6K and 4E-BP1 [38]. Inhibition of mTOR by CCI‑779 therefore reduces GPX4 synthesis, sensitizing cells to ferroptosis. However, mTOR activity also supports HAdV-11 replication by maintaining host translational capacity. Thus, mTOR blockade exerts a dual effect: it enhances cell-intrinsic ferroptotic death yet limits viral protein production and progeny spread. Additionally, HAdV-11-induced antioxidant responses—such as NRF2 activation reported for other viruses—may partially buffer GPX4 loss under combined treatment [39, 40], explaining why the further decrease in GPX4 did not always reach statistical significance. This duality underscores mTOR’s role as a rheostat that balances antioxidant defense and oncolytic virus propagation.

From a translational perspective, HAdV-11 represents a promising group B adenoviral platform characterized by CD46 and DSG2 receptor usage, distinctive biodistribution, and lower cross-reactivity with pre-existing HAdV-5 immunity. These features suggest improved potential for systemic delivery and repeated administration. Clinically validated oncolytic adenoviruses—such as H101, DNX-2401, enadenotucirev, and CG0070—demonstrate that adenoviral platforms can be delivered either systemically or intratumorally and can remodel the tumor microenvironment, but they also illustrate important translational constraints—dose-limiting inflammation, variable intratumoral penetration, and heterogeneous receptor expression among patient tumors [41–44]. Notably, group B and armed OAds (enadenotucirev and ONCOS-102) have demonstrated systemic delivery feasibility and the capacity to remodel the tumor microenvironment, particularly by promoting intratumoral CD8⁺ T-cell infiltration and enhancing responses to immunotherapy [4, 45].

Our findings provide mechanistic insight into how HAdV-11 triggers mitochondrial fission, mitophagy, and mTOR-regulated ferroptosis in ESCC cells—biological processes that may promote immunogenic tumor cell death and sensitize malignant cells to metabolic stress. This mechanistic dimension complements existing translational OAd platforms and could inform rational payload engineering or combination strategies.

We acknowledge that our study was limited to in vitro models. Although these experiments establish a strong mechanistic foundation, in vivo validation is required to determine whether HAdV-11–induced mitochondrial dysfunction and ferroptosis translate into tumor regression and therapeutic benefit. Known translational challenges—pre-existing anti-Ad immunity, hepatic sequestration, and heterogeneous receptor expression—will also need to be addressed. Furthermore, combination regimens with mTOR inhibitors or ferroptosis inducers may carry overlapping toxicities, such as inflammatory cytokine release or off-target lipid peroxidation, underscoring the importance of dose optimization and biomarker-guided patient selection.

To address these limitations, we have developed a prespecified in vivo validation protocol using both syngeneic Syrian hamster and ESCC xenograft models. These studies, conducted under randomized and blinded conditions with power justification, will evaluate HAdV-11 biodistribution (by qPCR and infectious titer), systemic safety (clinical chemistry, histopathology), and therapeutic efficacy (tumor growth inhibition, survival). Additional endpoints include intratumoral viral load, ultrastructural assessment of mitochondrial dynamics, and spatial immunoprofiling for ferroptosis and immune infiltration markers. These experiments are in progress and will be reported in subsequent work.

## Conclusion

In summary, our findings demonstrate that HAdV-11 triggers ferroptosis in ESCC cells via a dual mechanism involving both mitochondrial dysfunction and mTOR pathway modulation. The data indicates that therapeutic strategies promoting mitochondrial fission, suppressing protective mitophagy, or combining HAdV-11 with mTOR inhibitors could enhance its oncolytic potential. However, as these conclusions are based on in vitro experiments with limited replicates, they should be interpreted as preliminary. Further investigation through rigorous in vivo studies and clinical validation is required to establish whether mitochondrial fission and mTOR signaling represent viable predictive biomarkers and therapeutic targets for HAdV-11-based anticancer therapies.

## Supplementary Information

Supplementary Material 1.

Supplementary Material 2.

Supplementary Material 3.

Supplementary Material 4.

Supplementary Material 5.

## Data Availability

Data is provided within the manuscript or supplementary information files.

## Abbreviations

HAdV-11: Adenovirus Type 11 (Oncolytic Adenovirus Type 11)
OAd: Oncolytic Adenovirus
HAdV-5: Adenovirus Type 5
NDV: Newcastle Disease Virus
ESCC: Esophageal Squamous Cell Carcinoma
CD46: Cluster of Differentiation 46
DRP1: Dynamin-Related Protein 1
GPX4: Glutathione Peroxidase 4
TCID_50_: Tissue culture infectious dose 50%
PFU: Plaque-forming units
qPCR: Quantitative polymerase chain reaction
LC3: Microtubule-Associated Protein 1A/1B-Light Chain 3
PARK2: Parkin RBR E3 Ubiquitin Protein Ligase
PINK1: PTEN-Induced Kinase 1
VDAC: Voltage-Dependent Anion Channel
mTOR: Mechanistic Target of Rapamycin
PI3K: Phosphoinositide 3-Kinase
p-DRP1 (Ser637): Phosphorylated DRP1 at Serine 637
CCI-779: Temsirolimus (mTOR Inhibitor)
Fer-1: Ferrostatin-1
Fe²⁺: Ferrous Iron
GSH: Glutathione
Mdivi-1: Mitochondrial Division Inhibitor 1
FBS: Fetal Bovine Serum
JC-1: 5,5’,6,6’-Tetrachloro-1,1’,3,3’-Tetraethylbenzimi-dazolylcarbocyanine Iodide
MDC: Monodansylcadaverine
MitoSOX™: Mitochondrial Superoxide Indicator
MTG: MitoTracker Green
MTR: MitoTracker Red
MTS/PMS: 3-(4,5-dimethylthiazol-2-yl)-5-(3-carboxymethoxyphenyl)-2-(4-sulfophenyl)-2H-tetrazolium/Phenazine methosulfate
PI: Propidium Iodide
ROS: Reactive Oxygen Species
ΔΨm: Mitochondrial membrane potential
SARS-CoV-2: Severe Acute Respiratory Syndrome Coronavirus 2
E1A: Early region 1A
TERT: Telomerase reverse transcriptase
