# Supplementary material for: Oncolytic adenovirus type 11-induced ferroptosis of esophageal squamous cell carcinoma cells involves in mitochondrial impairment and the mTOR pathway
Source: BMC Cancer. 2026 Feb 24;26:423. doi: 10.1186/s12885-026-15735-7 (PMC13040906; doi:10.1186/s12885-026-15735-7)
Supplement: Supplementary file 1 — Supplementary Material 1. [file 12885_2026_15735_MOESM1_ESM.pdf]

Fig. 1B

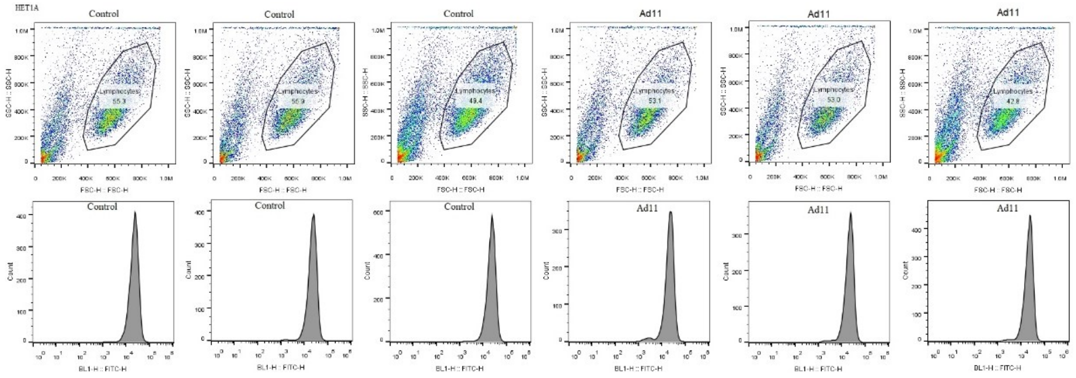
HET1A
Control
Control
Control
Ad11
Ad11
Ad11
Lymphocytes
SSC-H :: SSC-H
FSC-H :: FSC-H
Count
BL1-H :: FITC-H

Fig. 1D

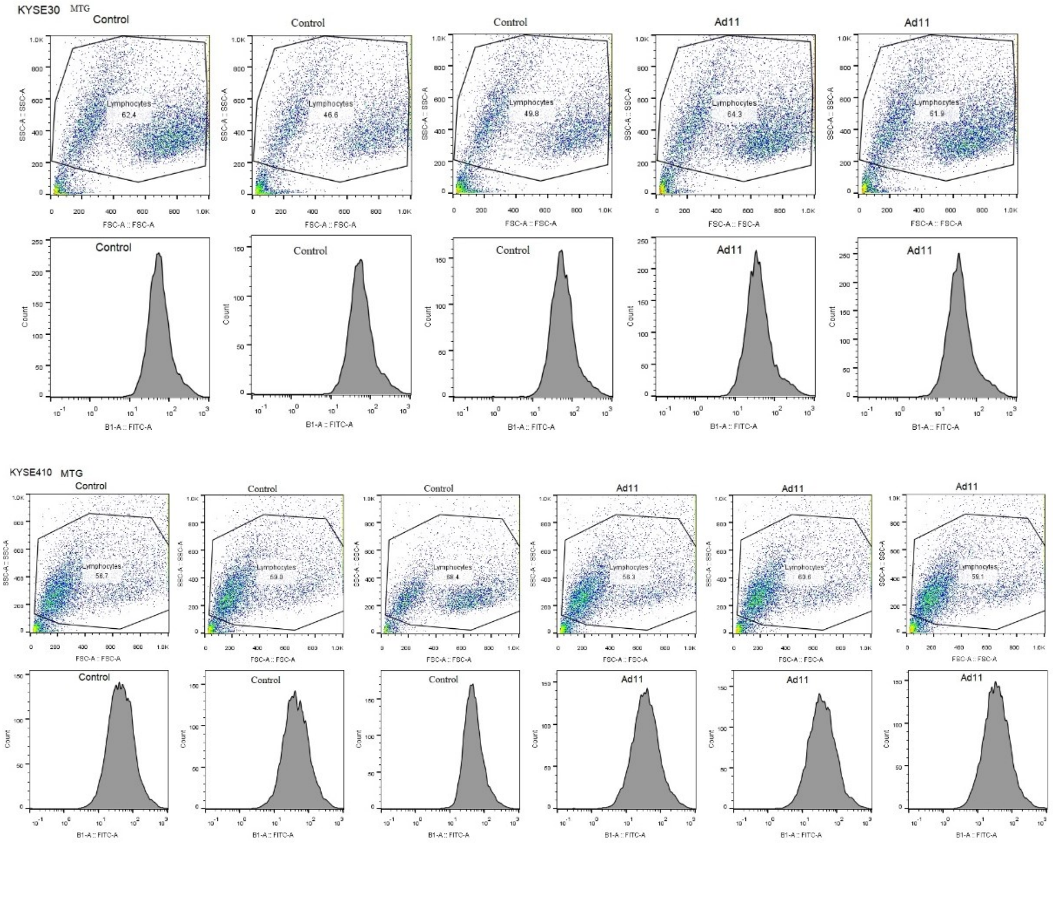
KYSE30 MTG
Control
Control
Control
Ad11
Ad11
Lymphocytes
SSC-A :: SSC-A
FSC-A :: FSC-A
Count
B1-A :: FITC-A
KYSE410 MTG
Control
Control
Control
Ad11
Ad11
Ad11

Fig. 1E

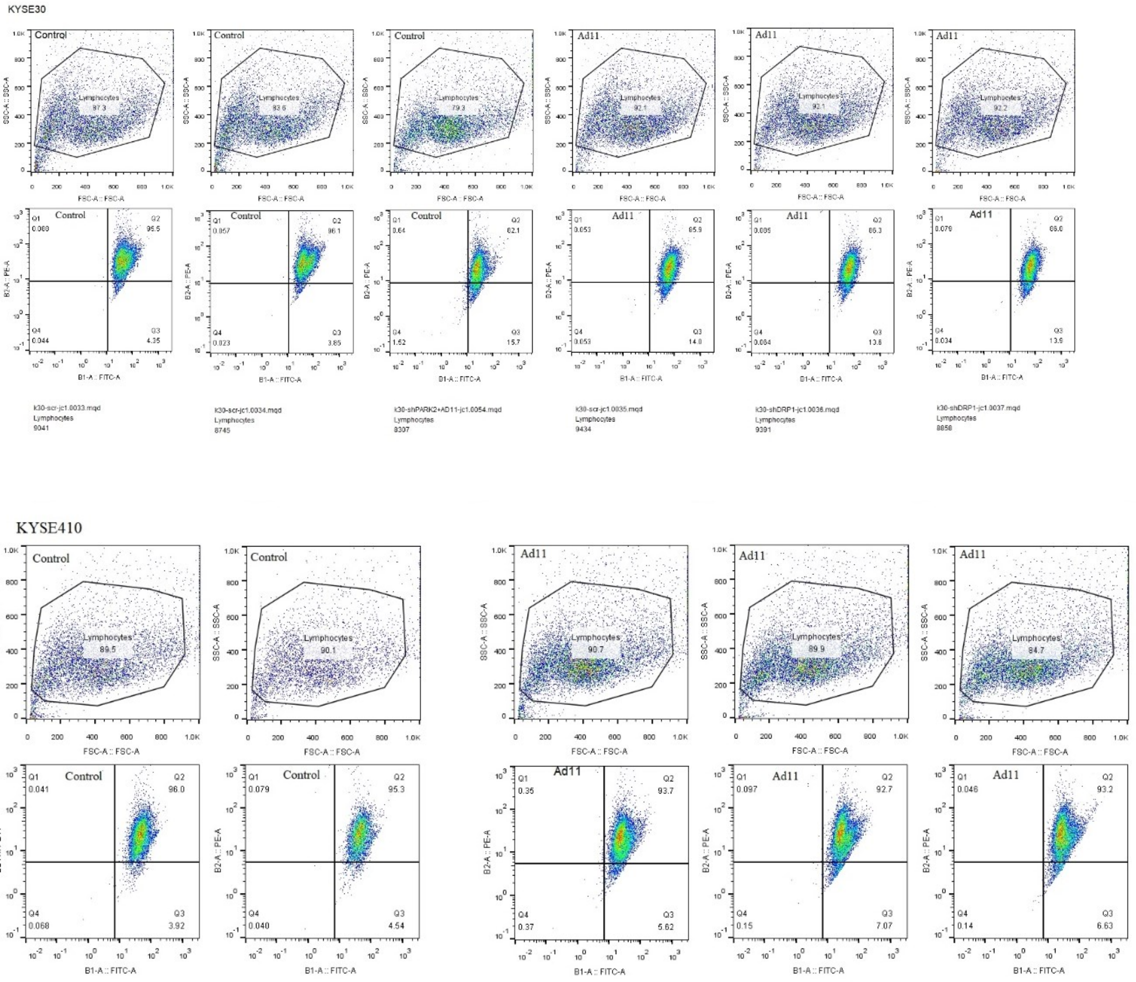
KYSE30
Control
Ad11
Lymphocytes
SSC-A :: SSC-A
FSC-A :: FSC-A
B2-A :: PE-A
B1-A :: FITC-A
k30-scr-jc1.0033.mqd
Lymphocytes
9041
k30-scr-jc1.0034.mqd
Lymphocytes
8745
k30-shPARK2+AD11-jc1.0054.mqd
Lymphocytes
8307
k30-scr-jc1.0035.mqd
Lymphocytes
9434
k30-shDRP1-jc1.0036.mqd
Lymphocytes
9391
k30-shDRP1-jc1.0037.mqd
Lymphocytes
8858
KYSE410
Control
Ad11
Lymphocytes
89.5
90.1
90.7
89.9
84.7
Q1
Q2
Q3
Q4

Fig. 2E

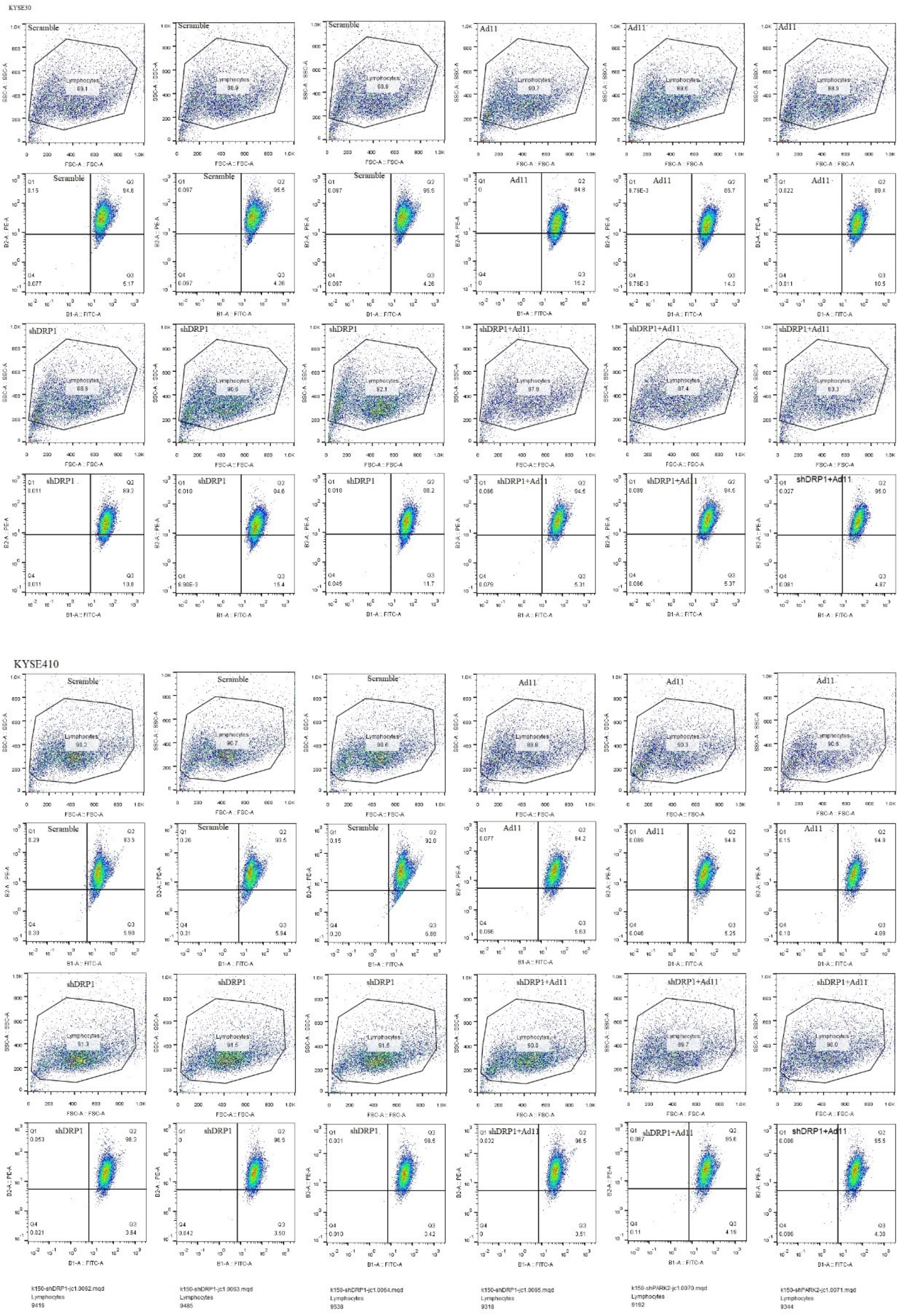
KYSE30
Scramble
Ad11
shDRP1
shDRP1+Ad11
KYSE410
k150-shDRP1-jc1.0062.mqd
Lymphocytes
9416
k150-shDRP1-jc1.0063.mqd
Lymphocytes
9485
k150-shDRP1-jc1.0064.mqd
Lymphocytes
9538
k150-shDRP1-jc1.0065.mqd
Lymphocytes
9318
k150-shPARK2-jc1.0070.mqd
Lymphocytes
9192
k150-shPARK2-jc1.0071.mqd
Lymphocytes
9344

Fig. 3A

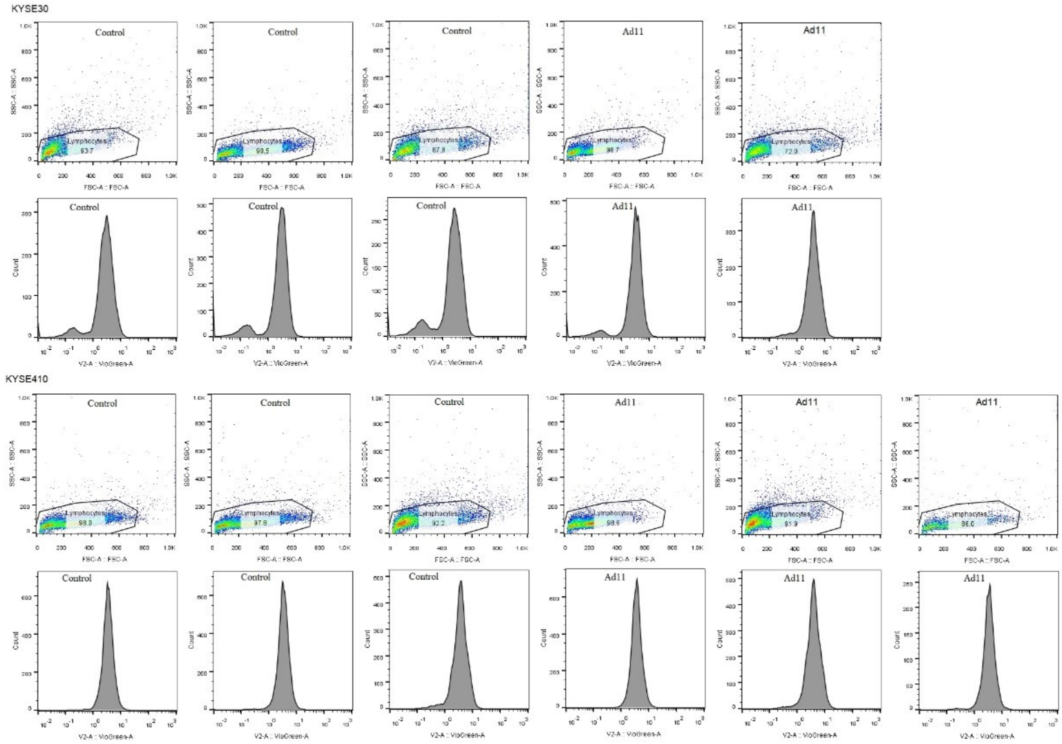
KYSE30
Control
Control
Control
Ad11
Ad11
Lymphocytes
SSC-A :: SSC-A
FSC-A :: FSC-A
Count
V2-A :: VioGreen-A
KYSE410
Control
Control
Control
Ad11
Ad11
Ad11

Fig. 4A

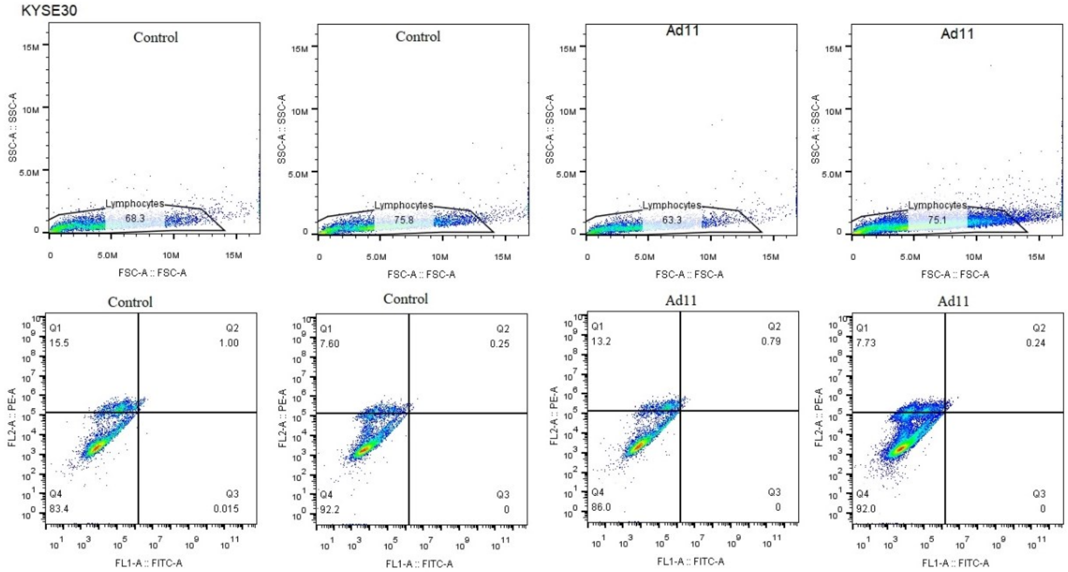
KYSE30
Control
Control
Ad11
Ad11
SSC-A :: SSC-A
FSC-A :: FSC-A
Lymphocytes
68.3
75.8
63.3
75.1
FL2-A :: PE-A
FL1-A :: FITC-A
Q1 15.5
Q2 1.00
Q4 83.4
Q3 0.015
Q1 7.60
Q2 0.25
Q4 92.2
Q3 0
Q1 13.2
Q2 0.79
Q4 86.0
Q3 0
Q1 7.73
Q2 0.24
Q4 92.0
Q3 0

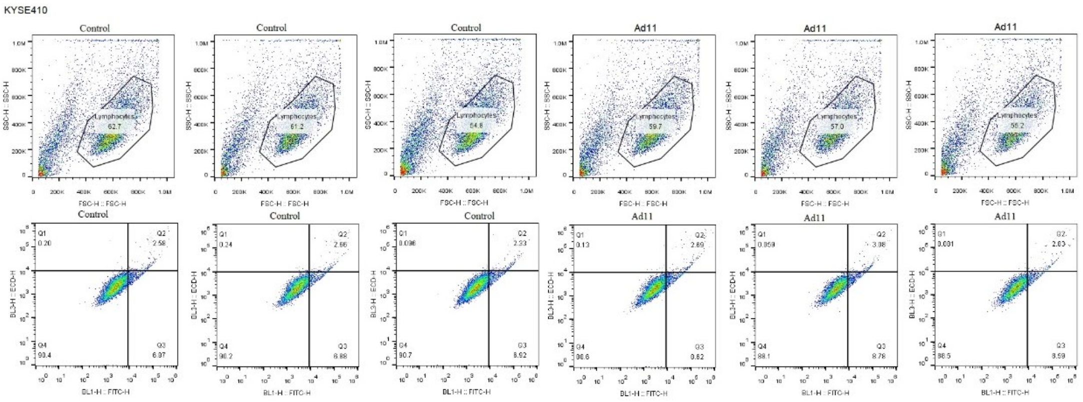
KYSE410
Control
Control
Control
Ad11
Ad11
Ad11

Fig. 4C

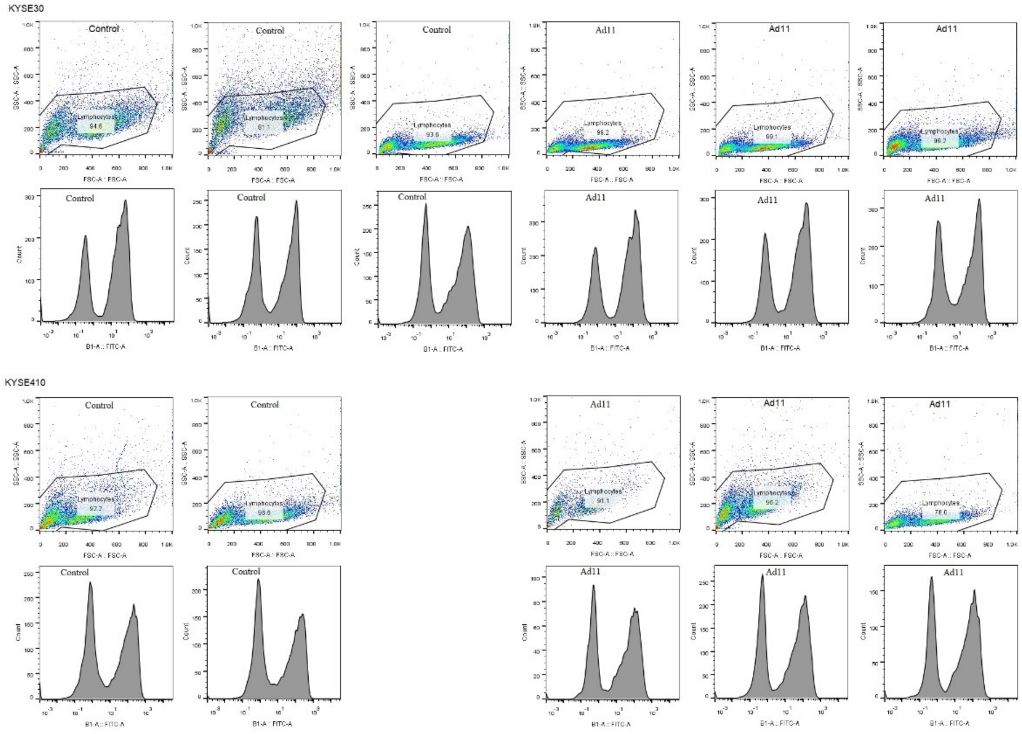
KYSE30
Control
Control
Control
Ad11
Ad11
Ad11
KYSE410
Control
Control
Ad11
Ad11
Ad11

Fig. 4E

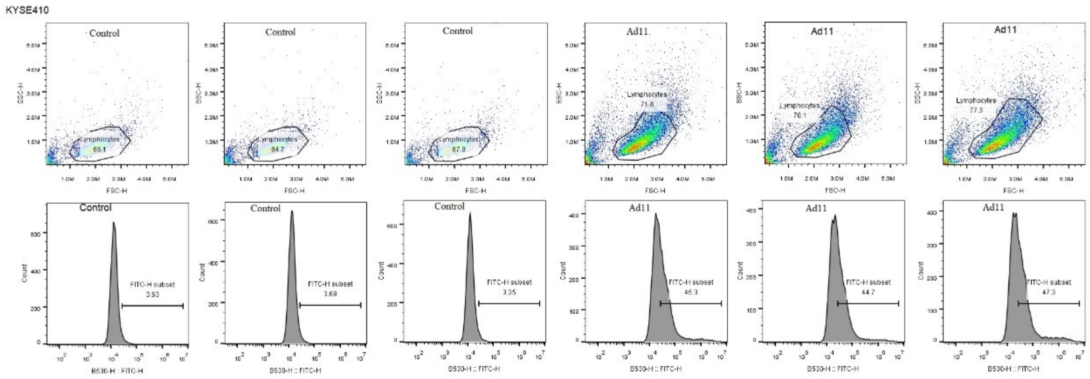
KYSE410
Control
Control
Control
Ad11
Ad11
Ad11

Fig. 4F

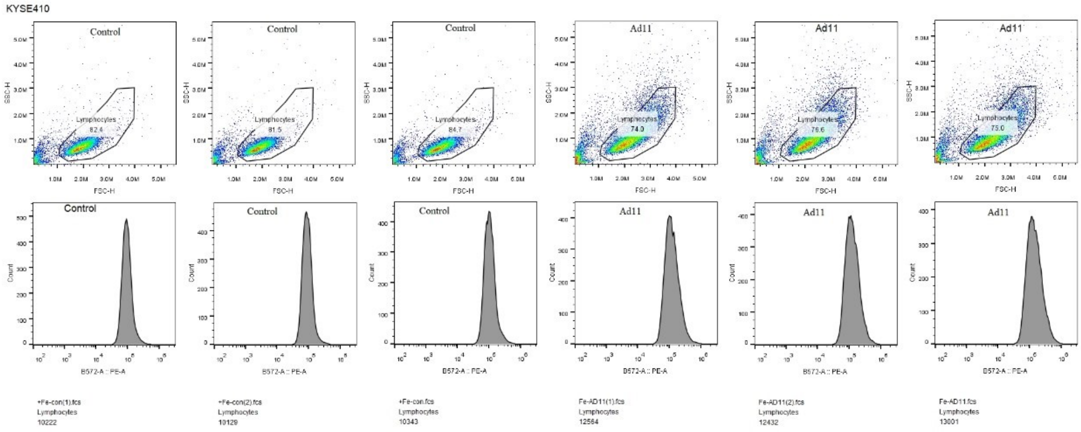
KYSE410
Control
Ad11
Lymphocytes
SSC-H
FSC-H
Count
B572-A :: PE-A

Fig. 4G

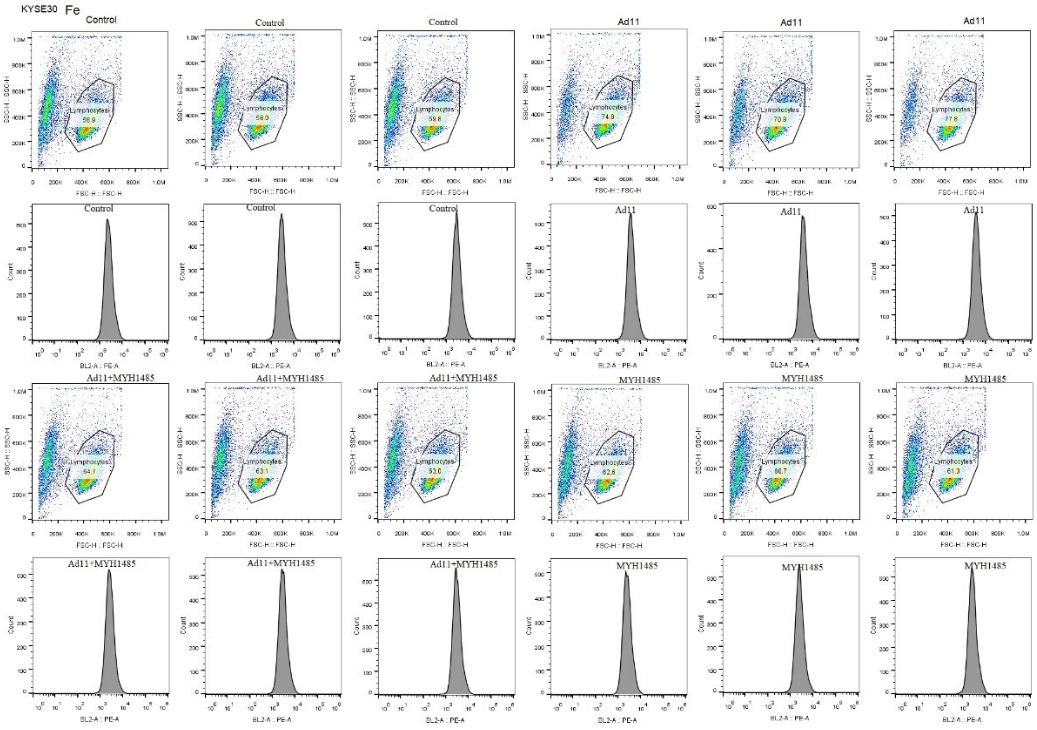
KYSE30 Fe
Control
Ad11
Ad11+MYH1485
MYH1485
Lymphocytes
SSC-H :: SSC-H
FSC-H :: FSC-H
Count
BL2-A :: PE-A

Fig. 5A

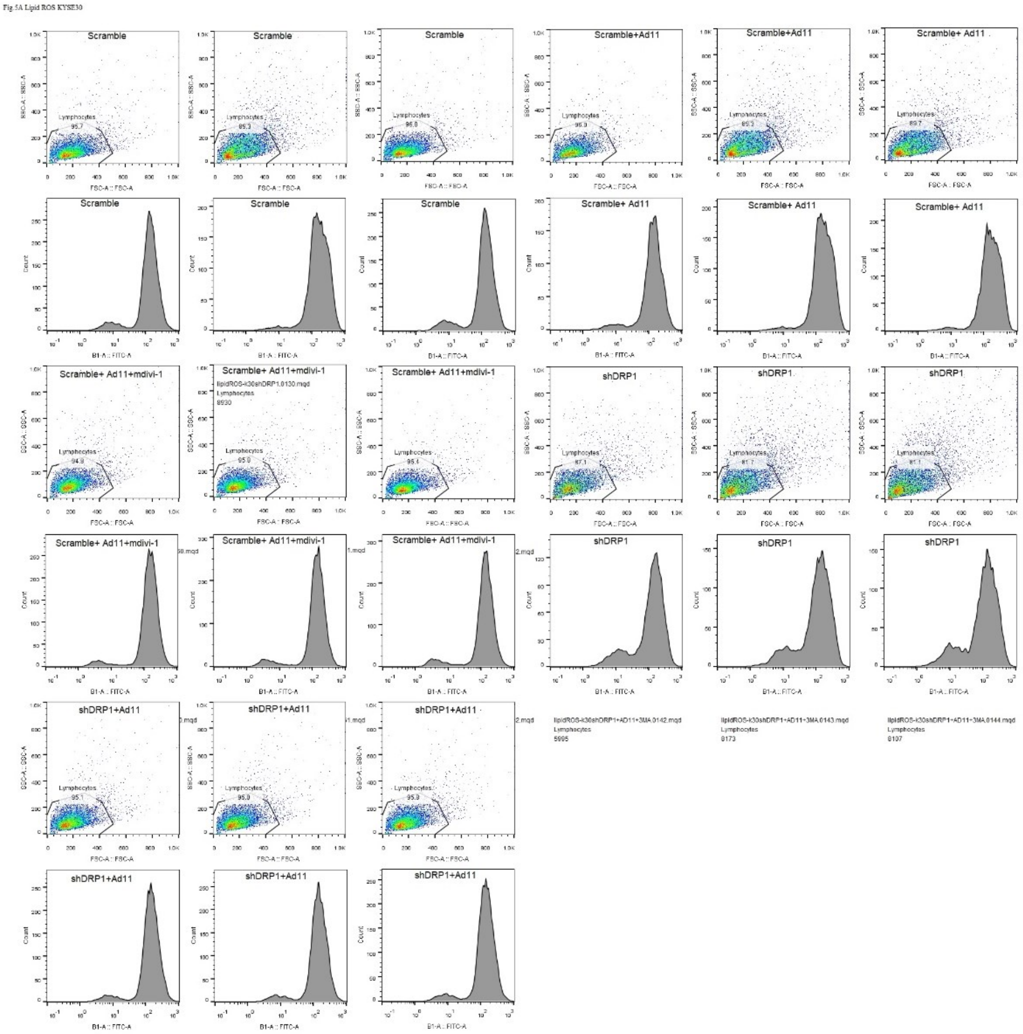
Fig.5A Lipid ROS KYSE30
Scramble
Scramble
Scramble
Scramble+Ad11
Scramble+Ad11
Scramble+ Ad11
Scramble
Scramble
Scramble
Scramble+ Ad11
Scramble+ Ad11
Scramble+ Ad11
Scramble+ Ad11+mdivi-1
Scramble+ Ad11+mdivi-1
Scramble+ Ad11+mdivi-1
shDRP1
shDRP1
shDRP1
Scramble+ Ad11+mdivi-1
Scramble+ Ad11+mdivi-1
Scramble+ Ad11+mdivi-1
shDRP1
shDRP1
shDRP1
shDRP1+Ad11
shDRP1+Ad11
shDRP1+Ad11
shDRP1+Ad11
shDRP1+Ad11
shDRP1+Ad11

Fig. 5B

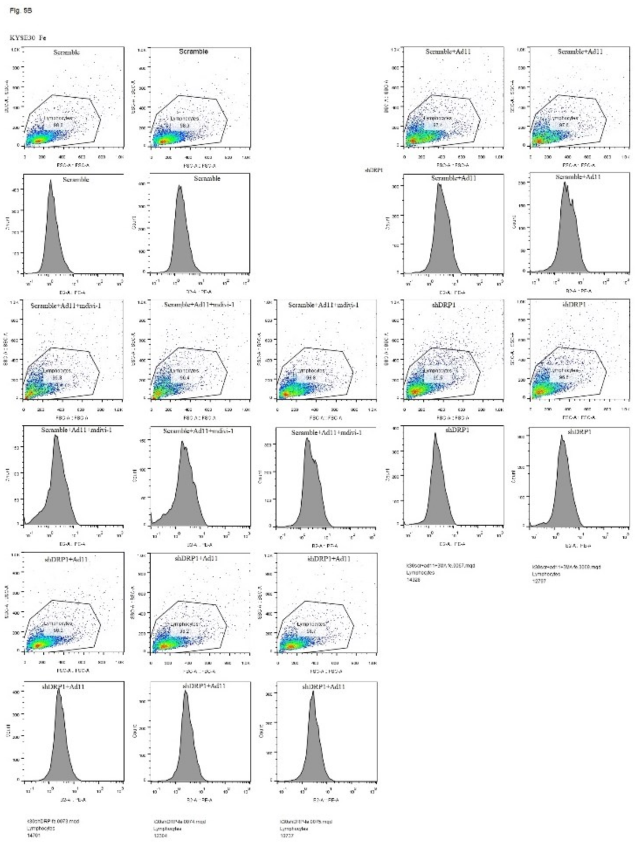
Fig. 5B
KYSE30 Fe
Scramble
Scramble+Ad11
Scramble+Ad11+mdivi-1
shDRP1
shDRP1+Ad11

Fig. 5E

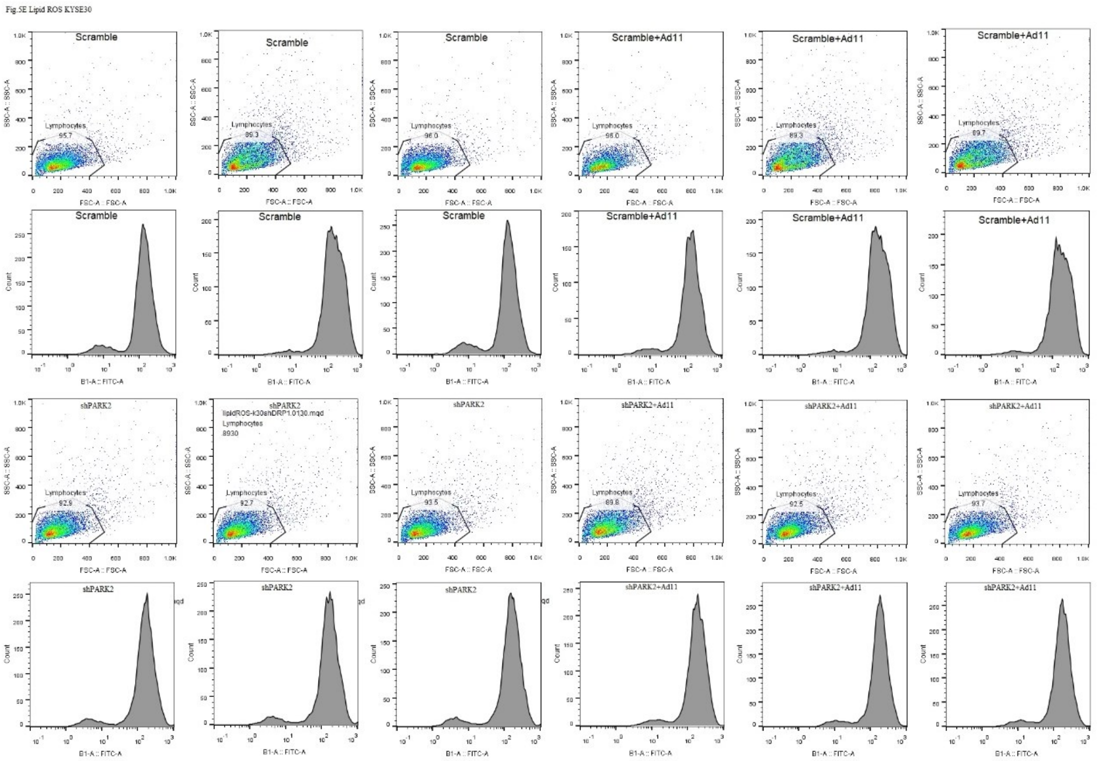
Fig.5E Lipid ROS KYSE30
Scramble
Scramble+Ad11
shPARK2
shPARK2+Ad11

Fig. 5E

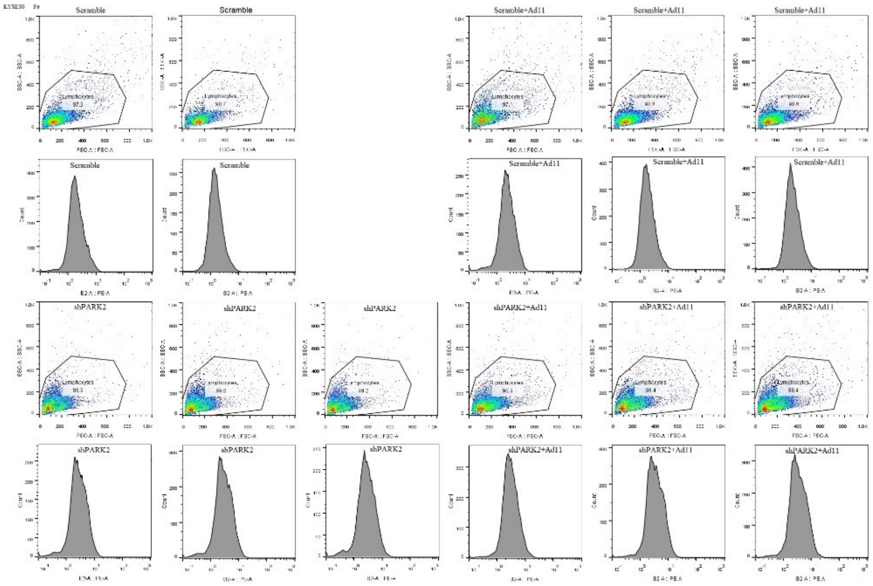
KYSE30
Scramble
Scramble+Ad11
shPARK2
shPARK2+Ad11
Lymphocytes
FSC-A :: FSC-A
SSC-A :: SSC-A
Count
B2-A :: PE-A

Fig. 6C

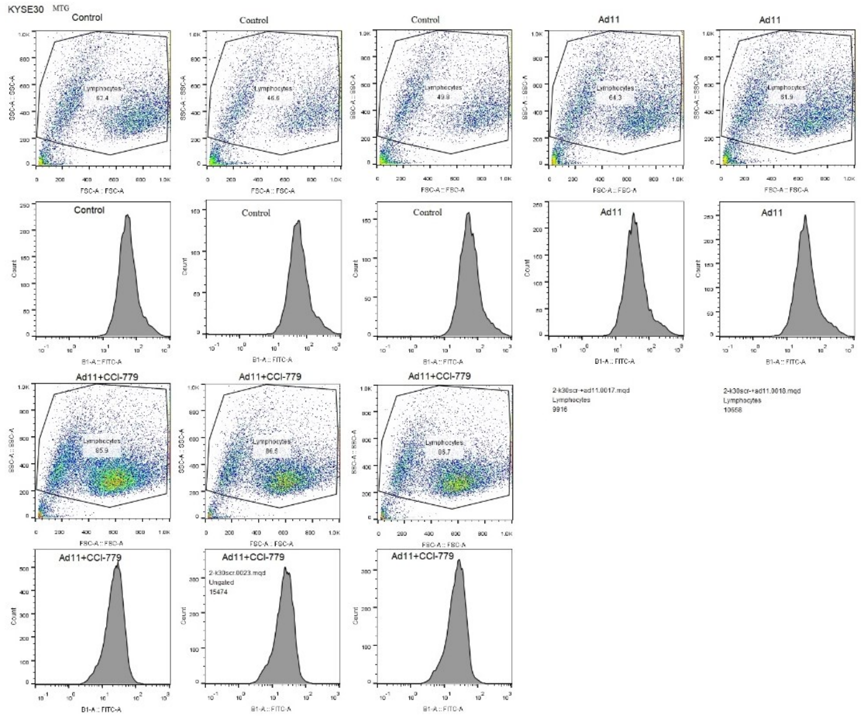
KYSE30 MTG
Control
Ad11
Ad11+CCI-779
Lymphocytes
FSC-A :: FSC-A
SSC-A :: SSC-A
Count
B1-A :: FITC-A

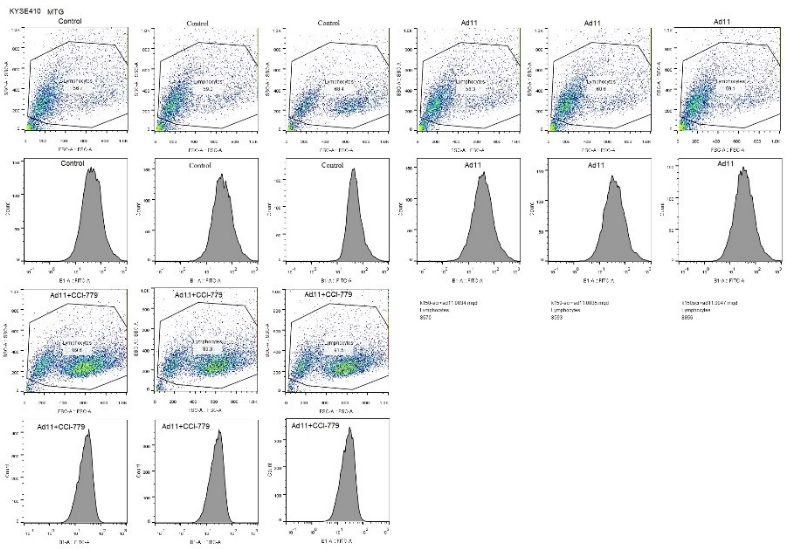
KYSE410 MTG
Control
Ad11
Ad11+CCI-779
Lymphocytes

Fig. 6H

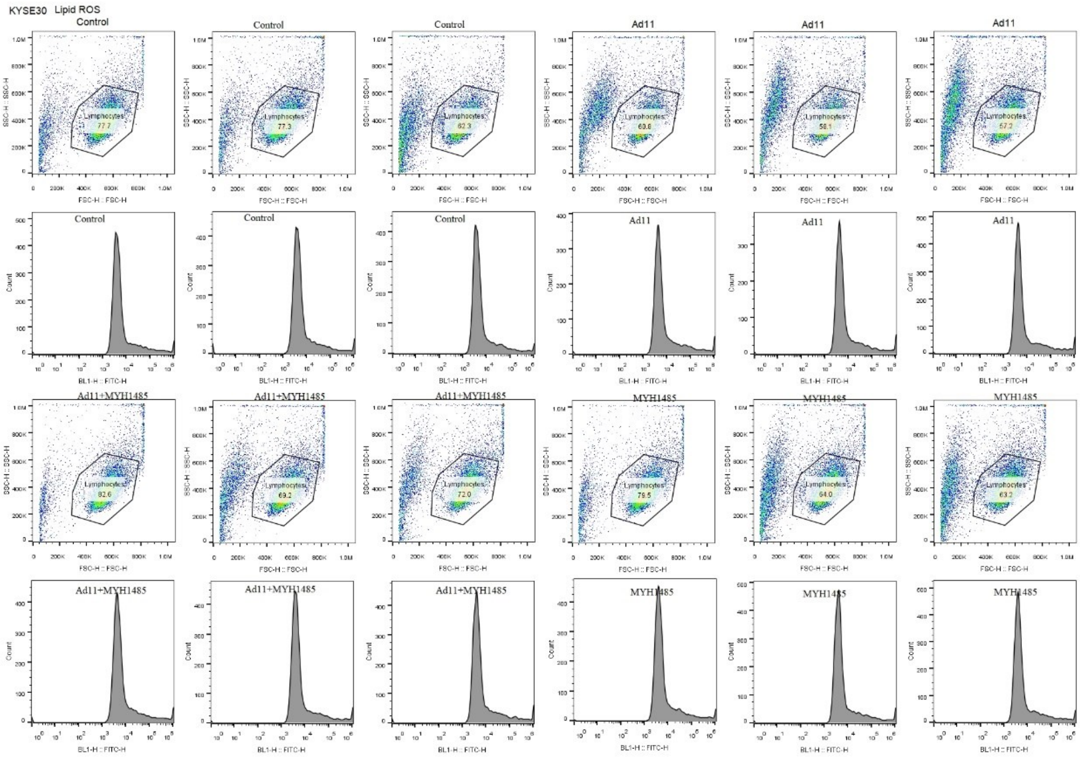
KYSE30 Lipid ROS
Control
Ad11
Ad11+MYH1485
MYH1485
Lymphocytes
SSC-H :: SSC-H
FSC-H :: FSC-H
Count
BL1-H :: FITC-H

KYSE30 Fe

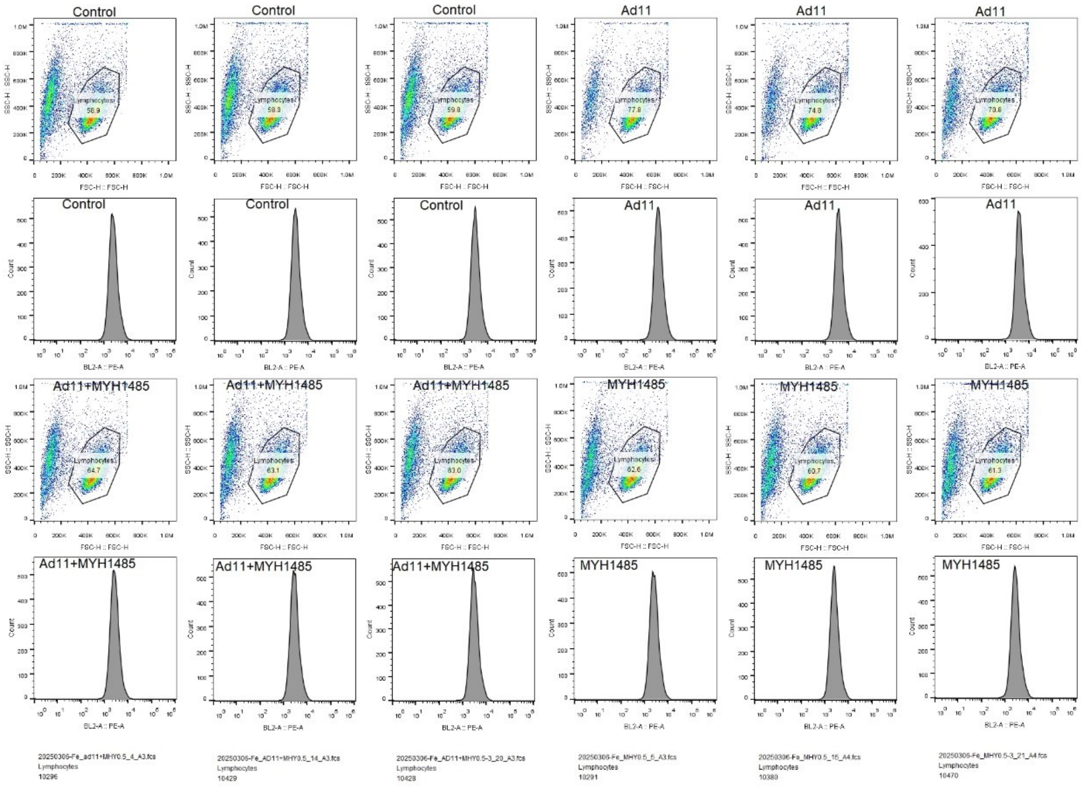

KYSE410 Lipid ROS

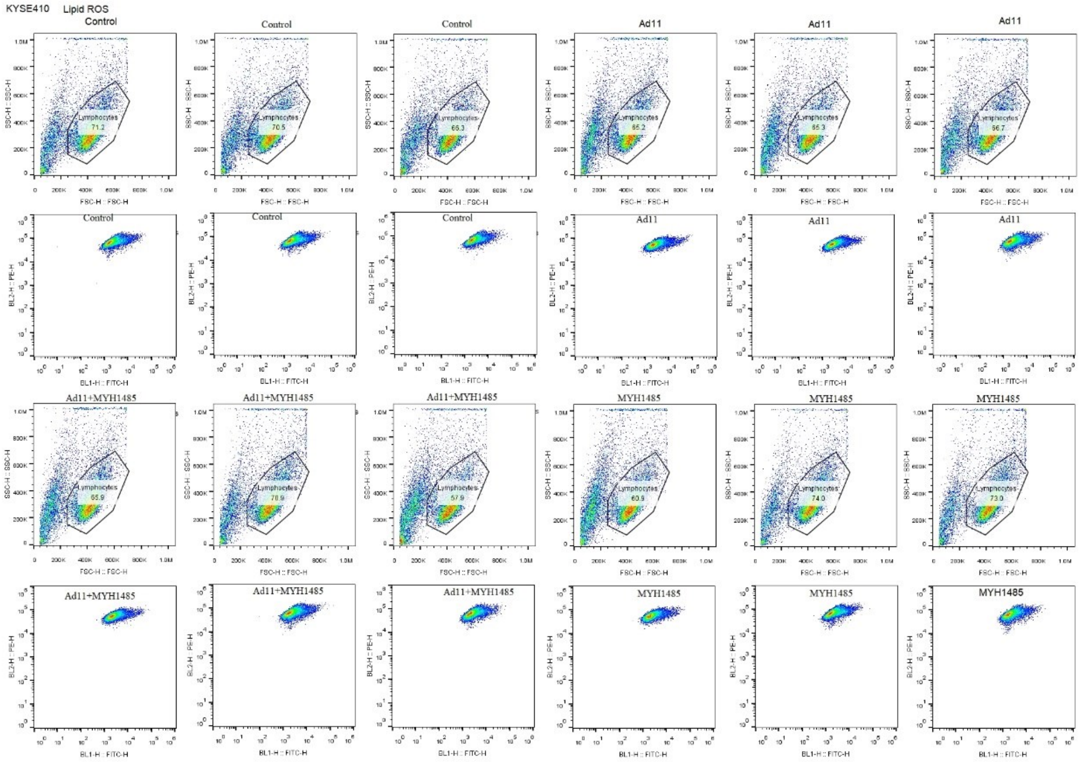

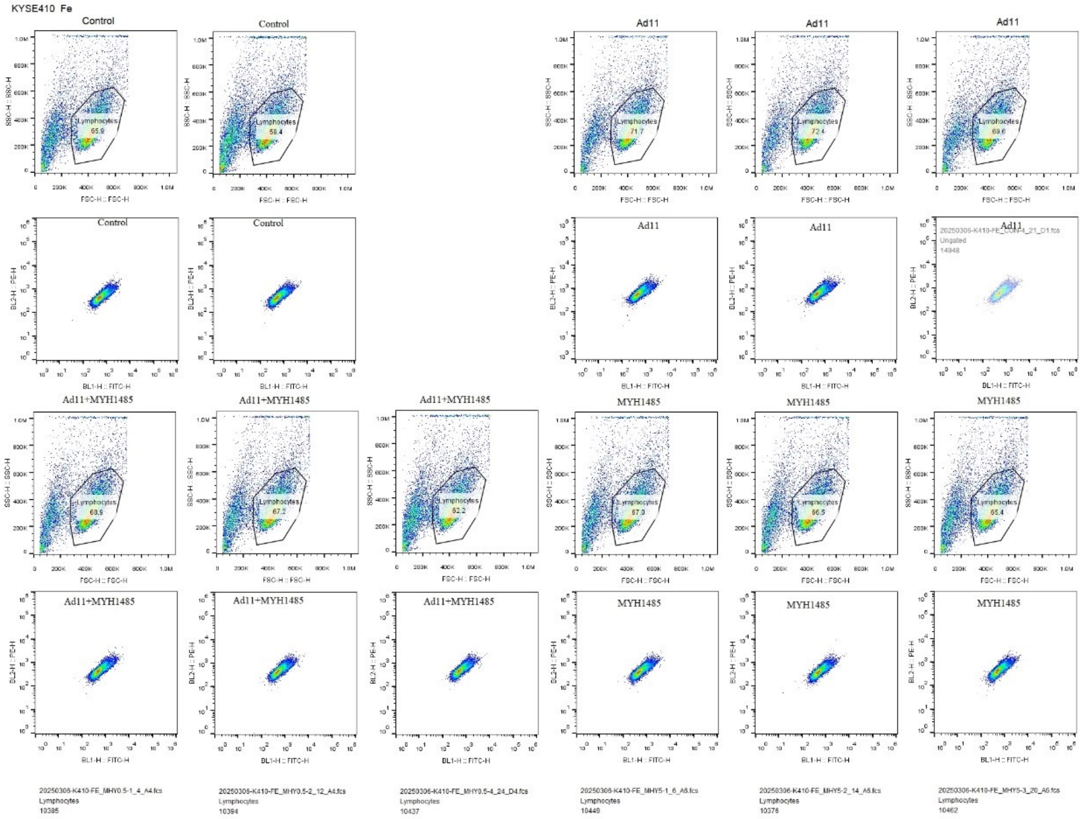
KYSE410 Fe
Control
Control
Ad11
Ad11
Ad11
Lymphocytes
65.9
58.4
71.7
72.4
69.6
SSC-H :: SSC-H
FSC-H :: FSC-H
BL2-H :: PE-H
BL1-H :: FITC-H
Ad11+MYH1485
Ad11+MYH1485
Ad11+MYH1485
MYH1485
MYH1485
MYH1485
68.9
67.2
62.2
67.0
66.5
65.4
20250306-K410-FE_MHY0.5-1_4_A4.fcs
Lymphocytes
10385
20250306-K410-FE_MHY0.5-2_12_A4.fcs
Lymphocytes
10394
20250306-K410-FE_MHY0.5-4_24_D4.fcs
Lymphocytes
10437
20250306-K410-FE_MHY5-1_6_A6.fcs
Lymphocytes
10449
20250306-K410-FE_MHY5-2_14_A6.fcs
Lymphocytes
10376
20250306-K410-FE_MHY5-3_20_A6.fcs
Lymphocytes
10462
