# Supplementary material for: Oncolytic adenovirus type 11-induced ferroptosis of esophageal squamous cell carcinoma cells involves in mitochondrial impairment and the mTOR pathway
Source: BMC Cancer. 2026 Feb 24;26:423. doi: 10.1186/s12885-026-15735-7 (PMC13040906; doi:10.1186/s12885-026-15735-7)
Supplement: Supplementary file 4 — Supplementary Material 4. [file 12885_2026_15735_MOESM4_ESM.pdf]

Supplementary Fig. 1

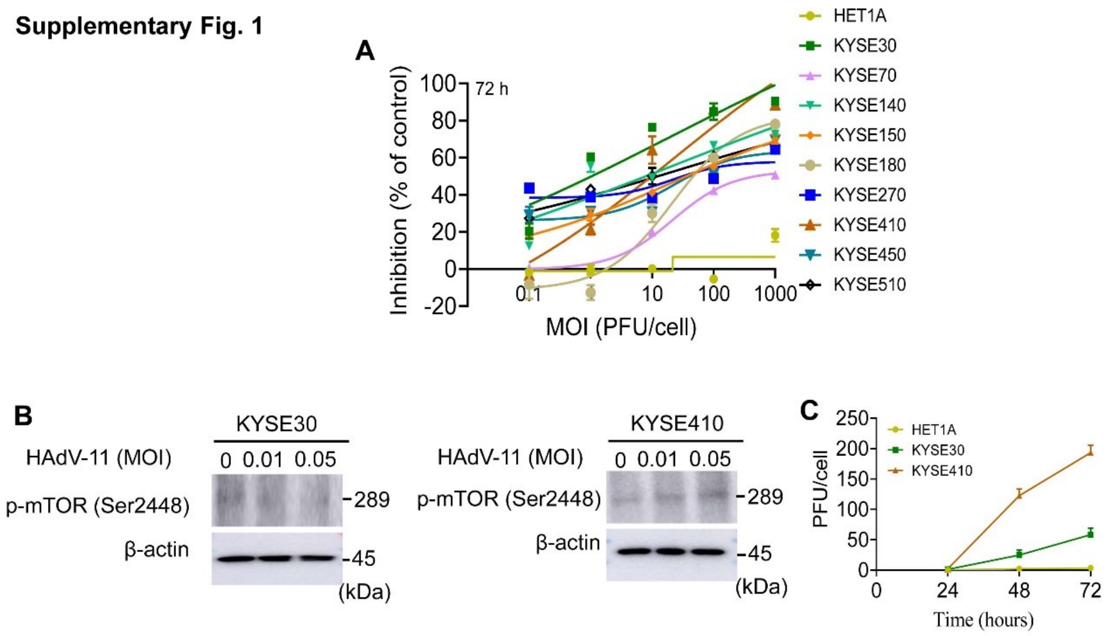

Supplementary Fig. 1. Dose–response cytotoxicity, mTOR signaling, and replication capacity of HAdV-11 in ESCC cells. (A) HET-1A cells and ESCC cells were treated with HAdV-11 for 72 h at different multiplicities of infection (MOI) of 0.1, 1, 10, 100 or 1000 plaque-forming units (PFU)/cell. Cell viability was evaluated using an MTS/PMS assay according to the manufacturers protocol. nonlinear dose–response fits was analysed. Experiments were performed in triplicate. (B) KYSE30 and KYSE410 cells were infected with Ad11 for 72 h at an MOI of 0.01 or 0.05, and Western blot analysis of p-mTOR (S2448) expressions. (C) HET-1A, KYSE30 or KYSE10 Cells were infected with HAdV-11 at an MOI of 2 for 24, 48, 72 h. Infectious virus production was assessed by titration on JH293 cells and the titer as PFU/cell calculated. Data are cumulative results from 3 experiments (A and C) or are representative of 3 independent experiments (B). Data in C are presented as means ± SD, two-tailed t-test (n=3).

Supplementary Fig. 2

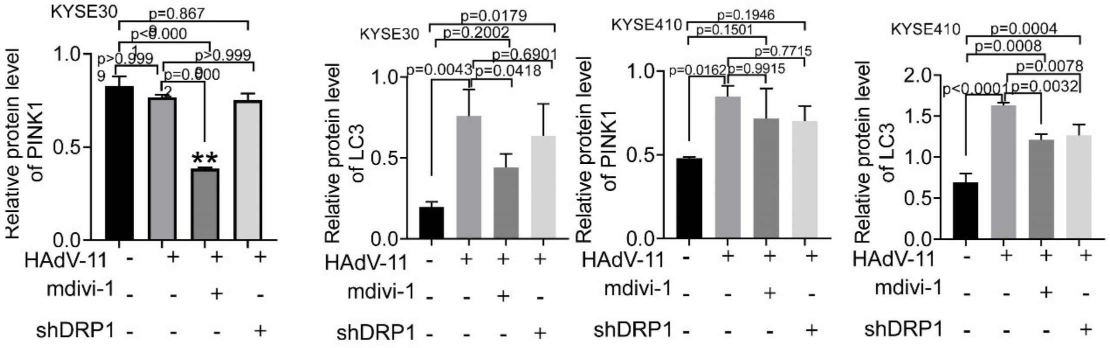

Supplementary Fig. 2. Quantification of Pink1 and LC3 proteins in KYSE30 cells and KYSE410 cells. Cells were treated with mdivi-1 at concentrations of 10 μM and 20 μM, respectively, for 2 hours, and then pretreated cells or pre-treated cells transduced with DRP1-shRNA were treated with 2 PFU/cell of Ad11 for 72 h, and Western blot analysis of LC3 and PTEN-induced kinase 1 (PINK1) expressions. Data are representative of 3 independent experiments. Data are presented as mean ± SD. Differences analyzed using a one-way ANOVA with Bonferroni's post hoc correction.

Supplementary Fig. 3

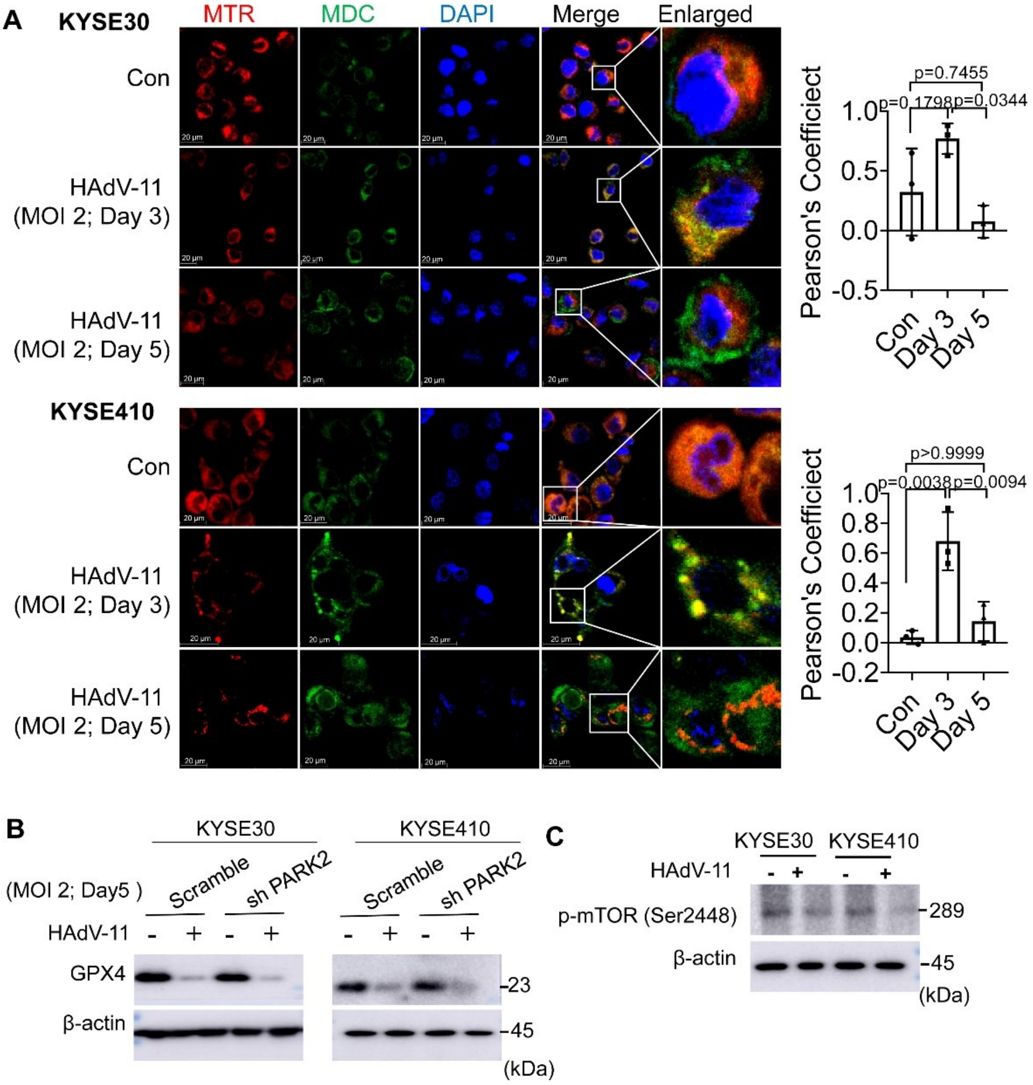

Supplementary Fig. 3. Temporal dynamics of mitophagy and ferroptosis regulation by Ad11. (A) Temporal dynamics of mitophagy. Confocal images of monodansylcadaverine (MDC; green) and MitoTracker Deep Red (MTR, red) co-localization in KYSE30 and KYSE410 cells infected with Ad11 (MOI = 2) on Days 3 and 5. The Pearson correlation coefficient was used to quantify the degree of colocalization. Data are presented as means ± SD (n = 3). Differences analyzed using a one-way ANOVA with Bonferroni's post hoc correction. (B) KYSE30 and KYSE410 cells were infected with Ad11 for Day5 at an MOI of 2. Western blots of GPX4 in Ad11-infected scrambled-shRNA transduced cells and PARK2-shRNA transduced cell lines. (C) KYSE30 and KYSE410 cells were infected with Ad11 for 72 h at an MOI of 2, and Western blot analysis of p-mTOR (S2448) expressions. Data are representative of 3 independent experiments (A B, C).
