# Supplementary material for: Oncolytic adenovirus type 11-induced ferroptosis of esophageal squamous cell carcinoma cells involves in mitochondrial impairment and the mTOR pathway
Source: BMC Cancer. 2026 Feb 24;26:423. doi: 10.1186/s12885-026-15735-7 (PMC13040906; doi:10.1186/s12885-026-15735-7)
Supplement: Supplementary file 5 — Supplementary Material 5. [file 12885_2026_15735_MOESM5_ESM.pdf]

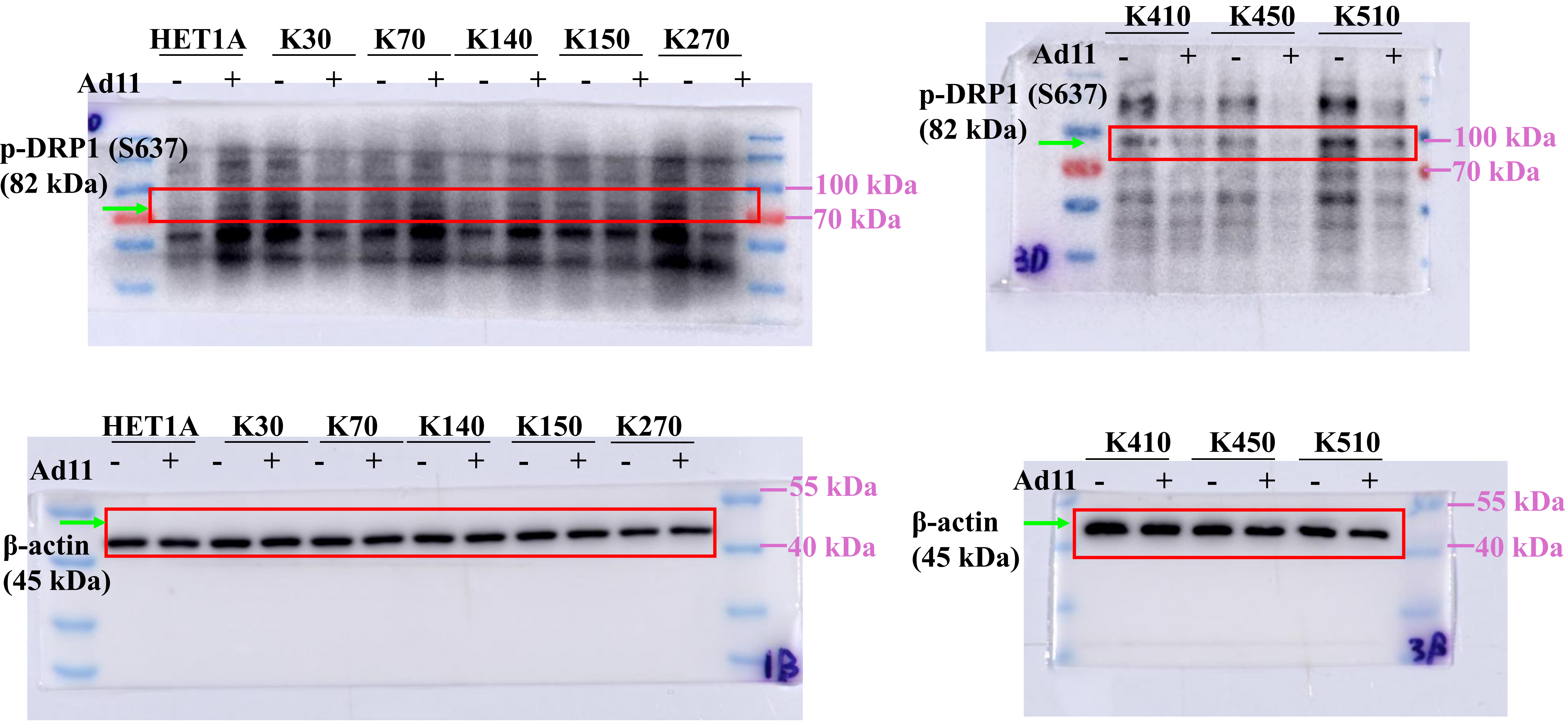

Supplementary Fig. 1: Complete uncropped western blot data of Fig. 1F

Used in Fig. 1F

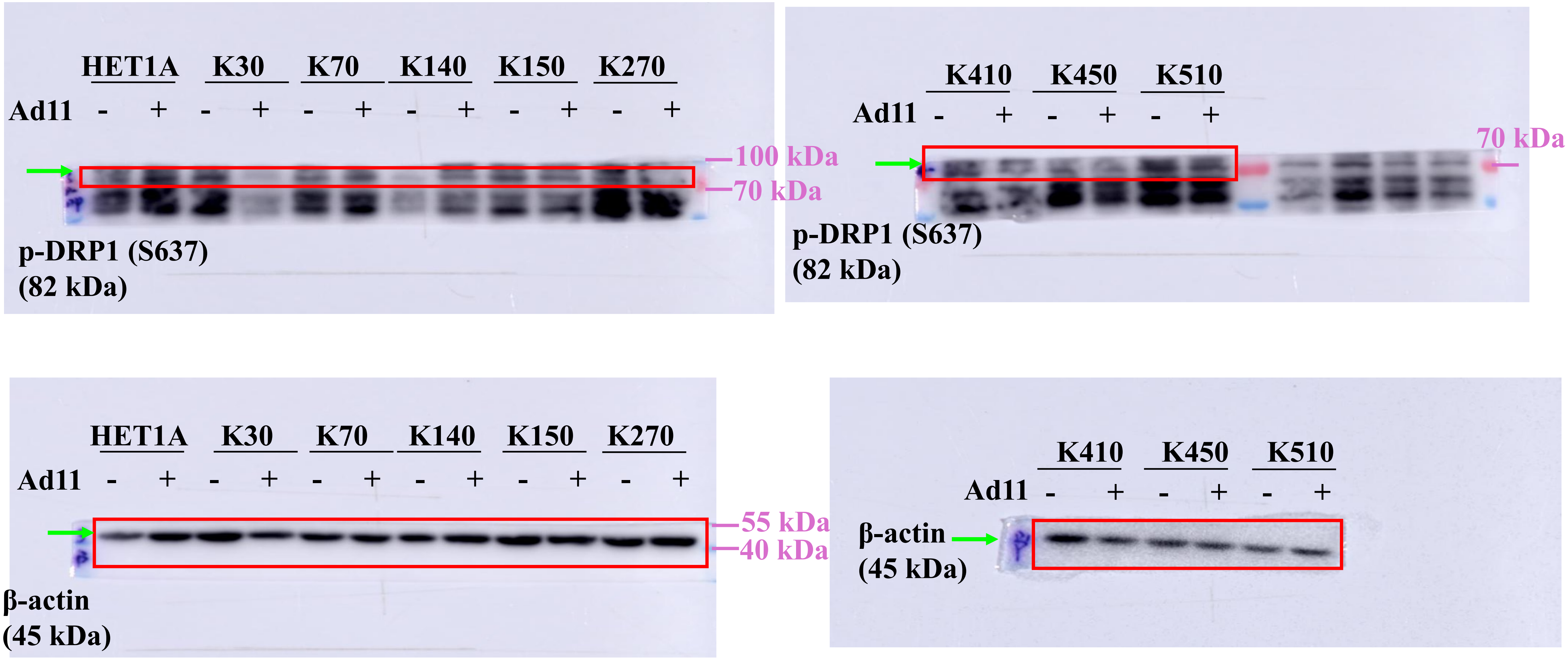

Supplementary Fig. 2: Complete uncropped western blot data of Fig. 1F

Replicate 2

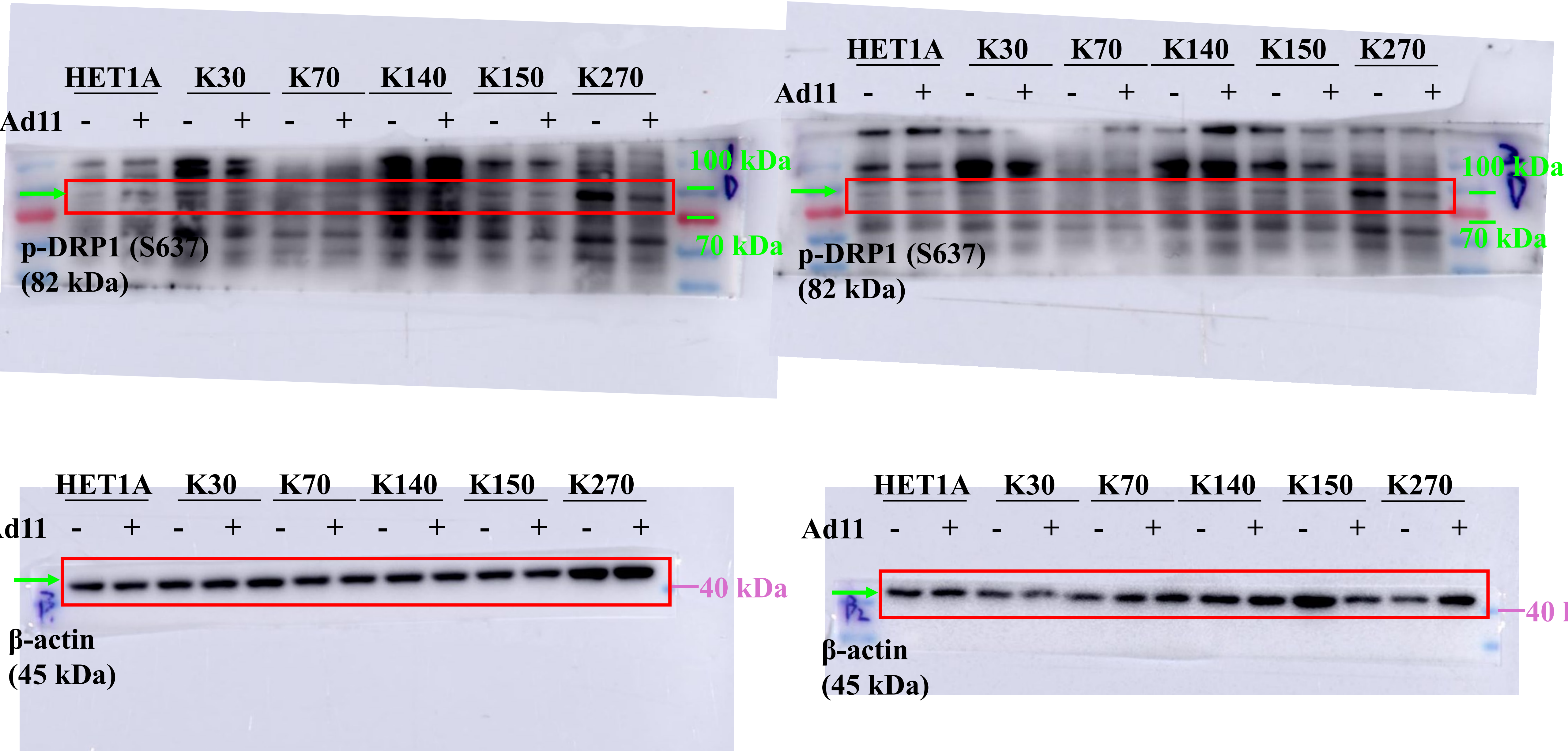

Supplementary Fig. 3: Complete uncropped western blot data of Fig. 1F

Replicate 3

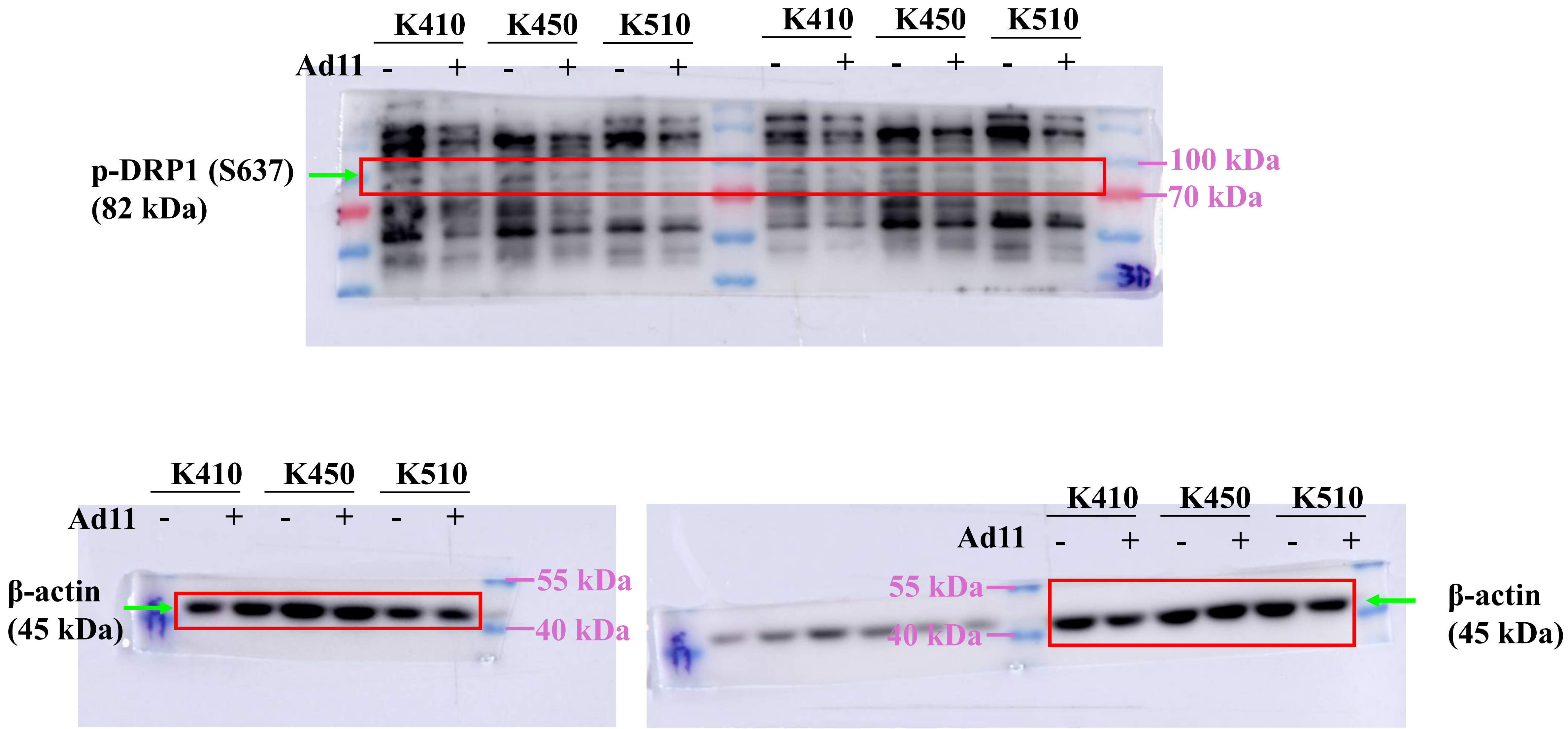

Supplementary Fig. 4: Complete uncropped western blot data of Fig. 1F

Replicate 2/3

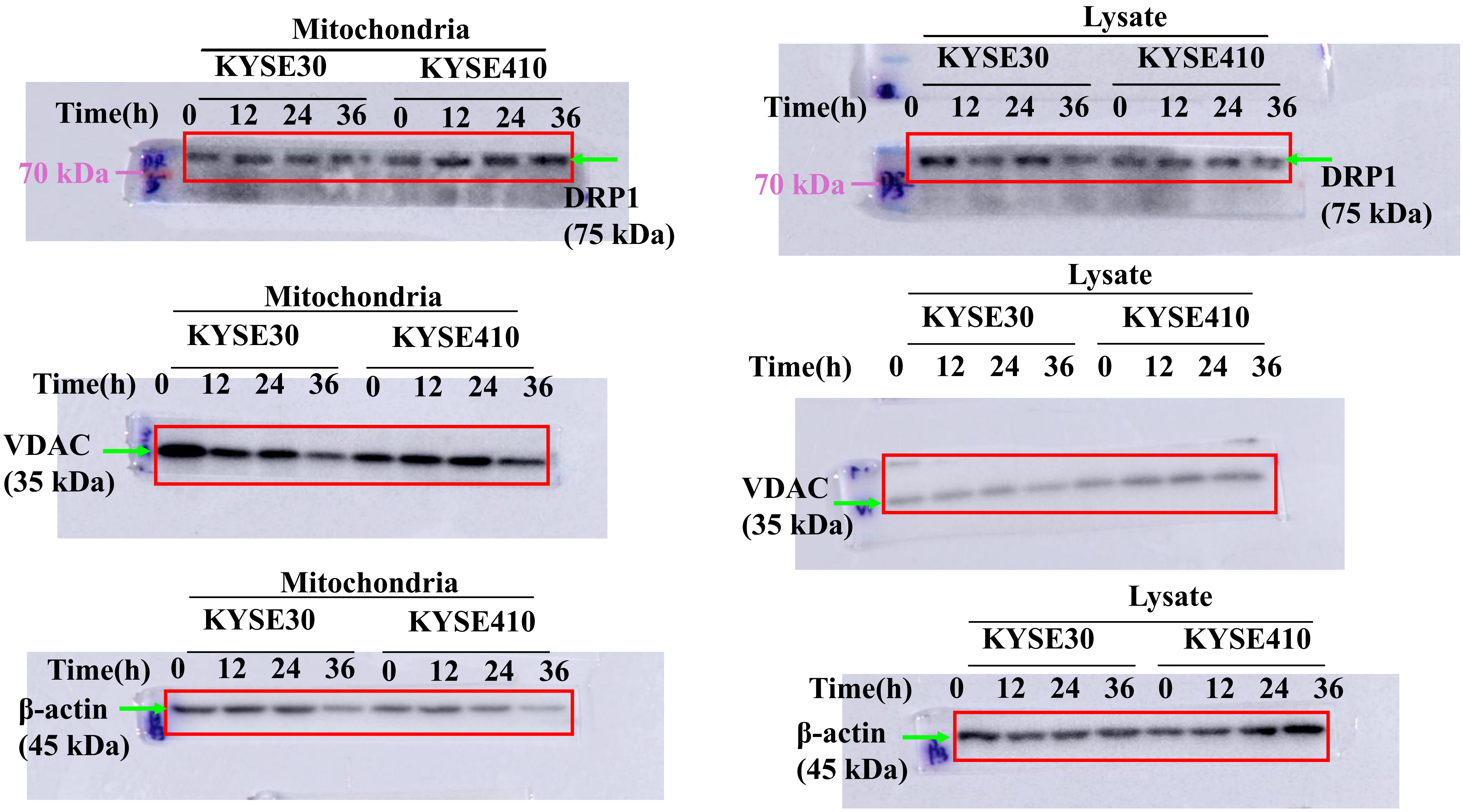

Supplementary Fig. 5: Complete uncropped western blot data of Fig. 1G

Used in Fig. 1G

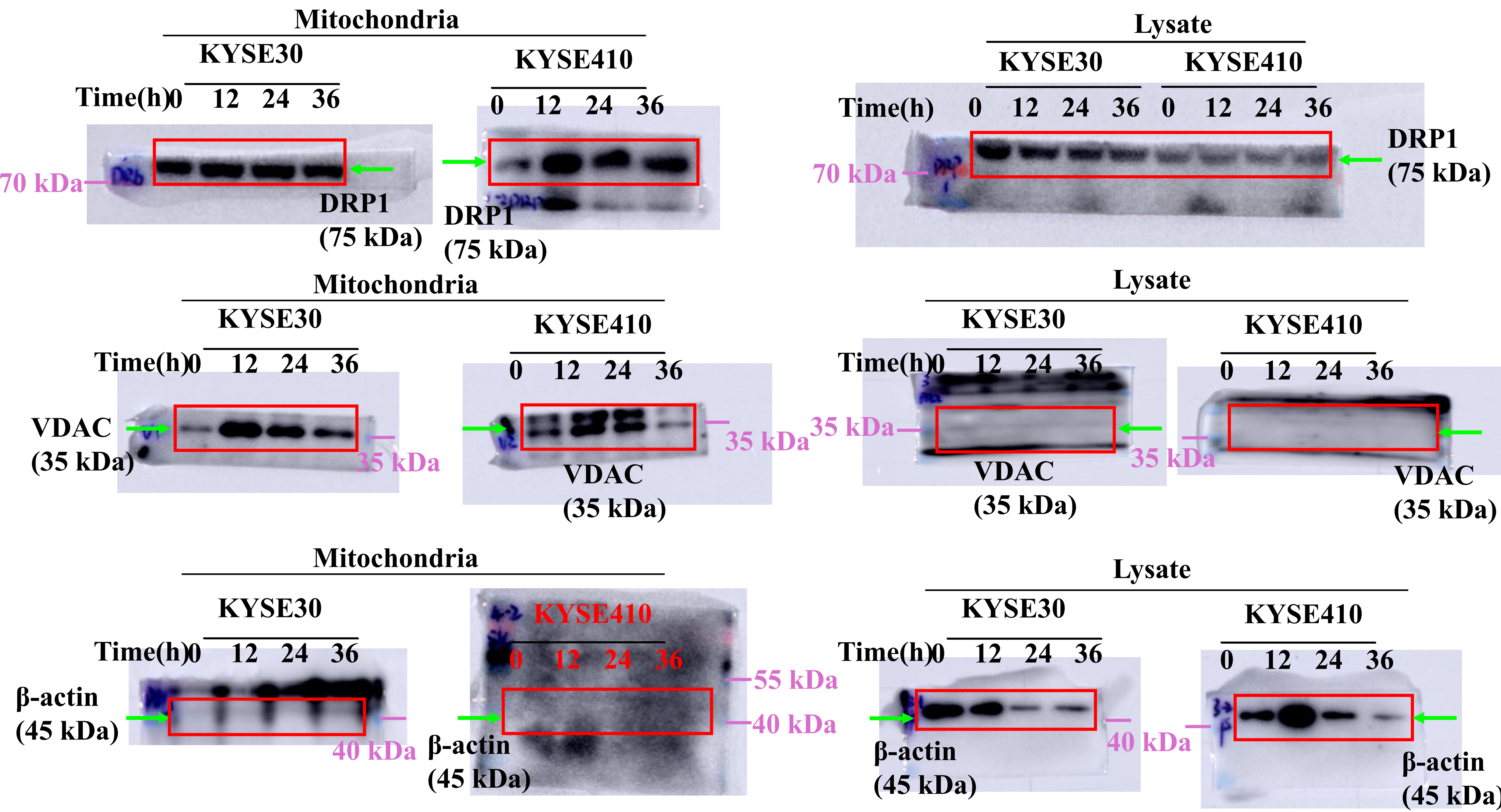

Supplementary Fig. 6: Complete uncropped western blot data of Fig. 1G Replicate 2

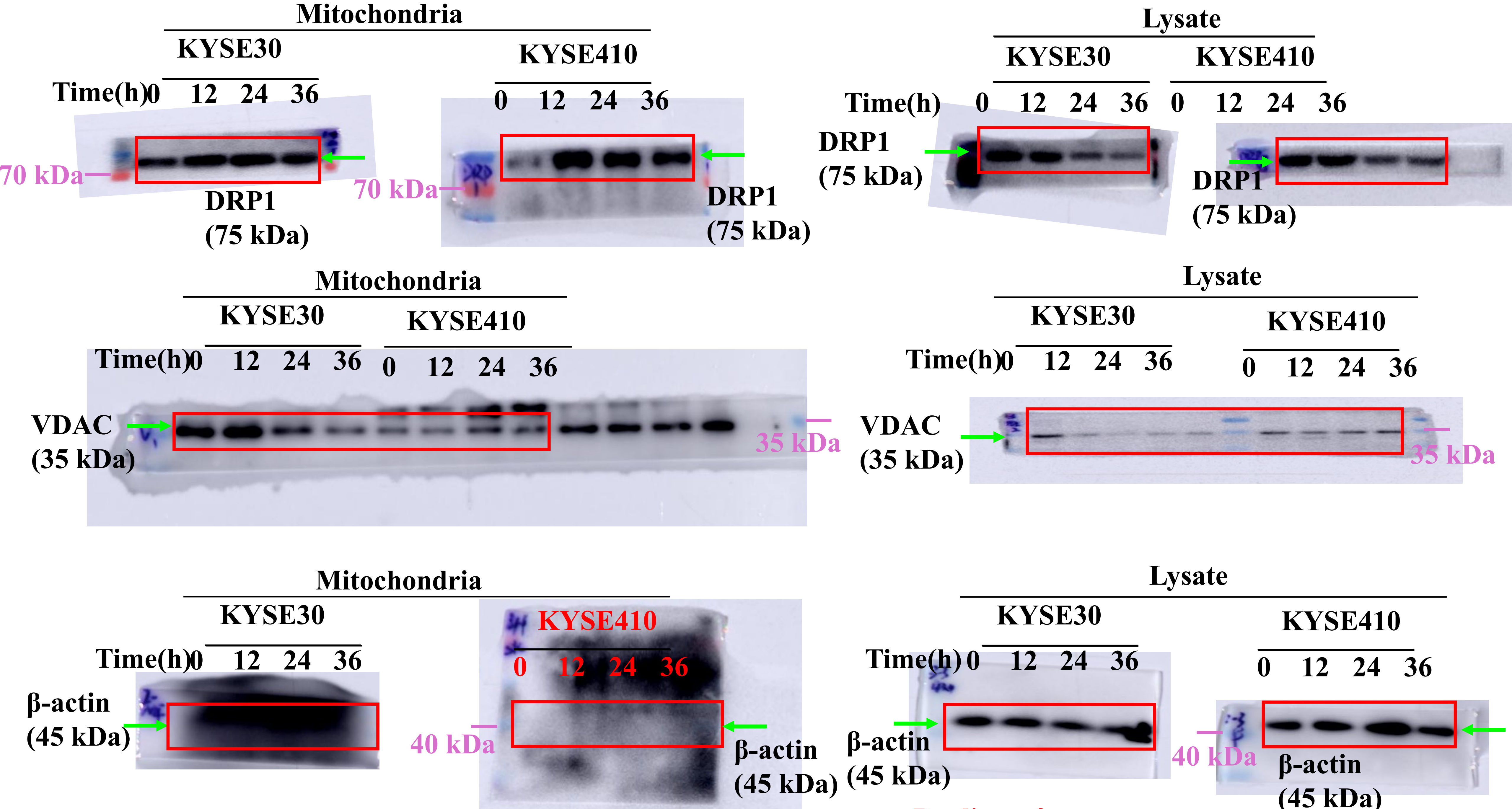

Supplementary Fig. 7: Complete uncropped western blot data of Fig. 1G Replicate 3

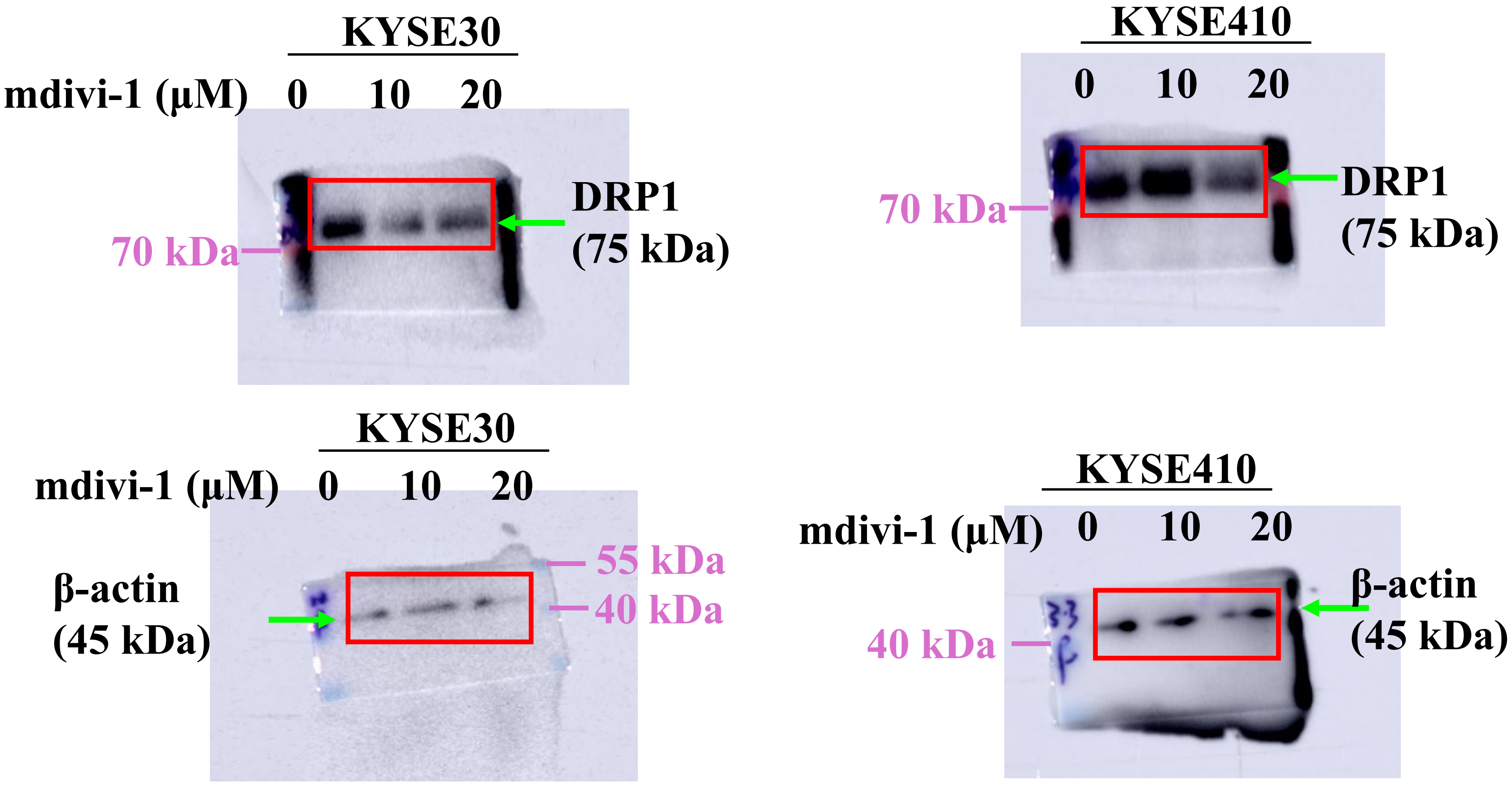

Supplementary Fig. 8: Complete uncropped western blot data of Fig. 2A

Used in Fig. 2A

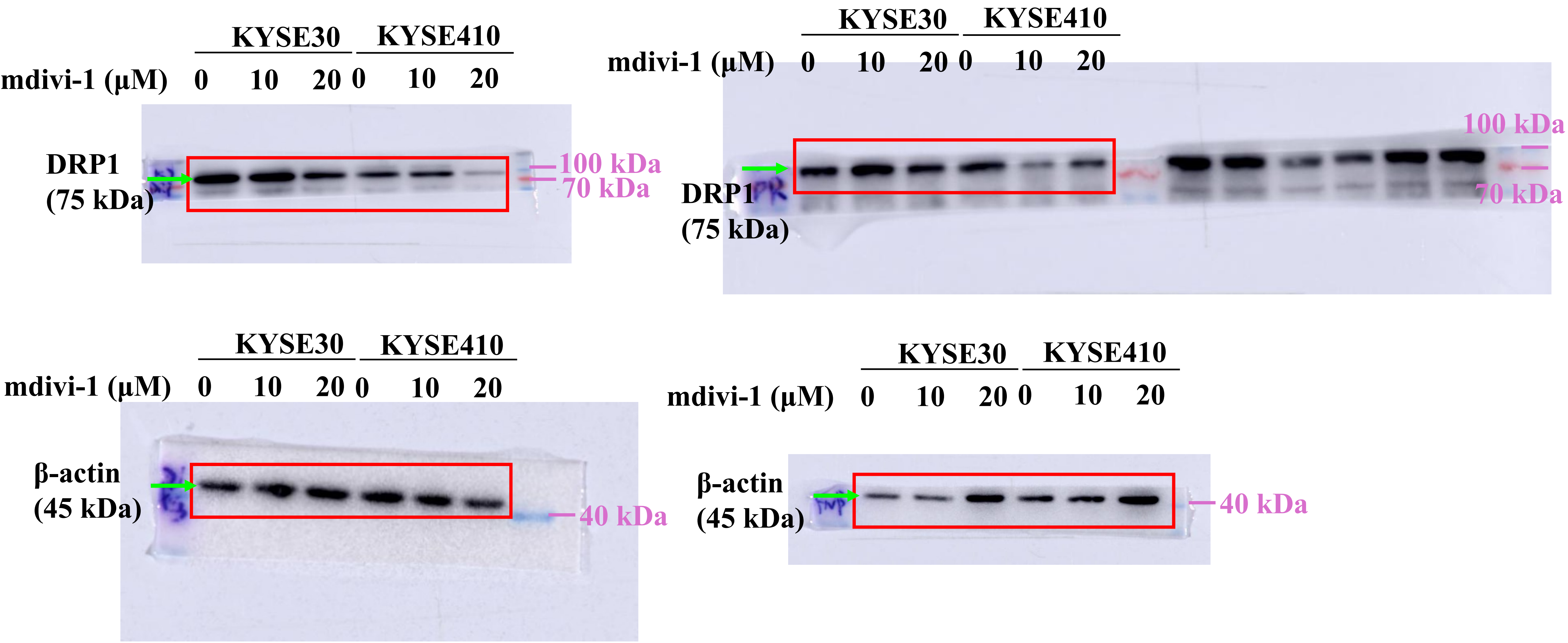

Supplementary Fig. 9: Complete uncropped western blot data of Fig. 2A

Replicate 2/3

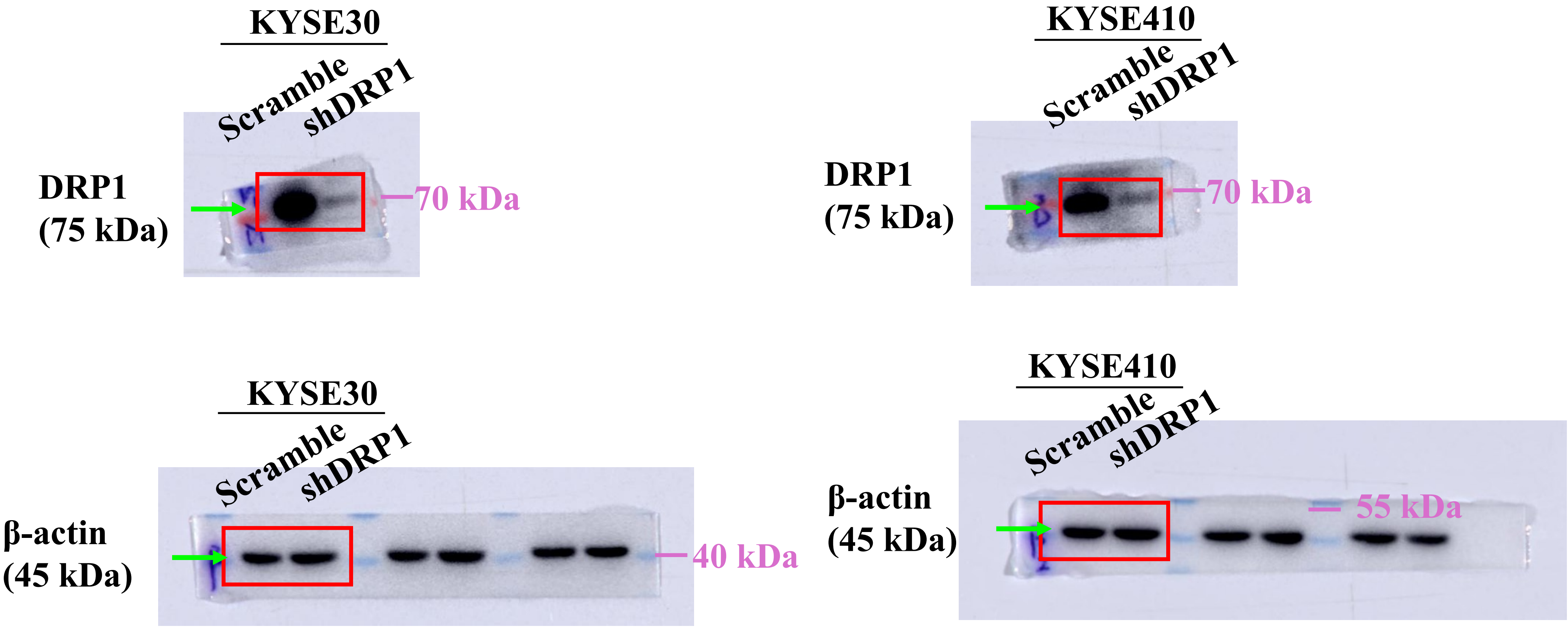

Supplementary Fig. 10: Complete uncropped western blot data of Fig. 2D

Used in Fig. 2D

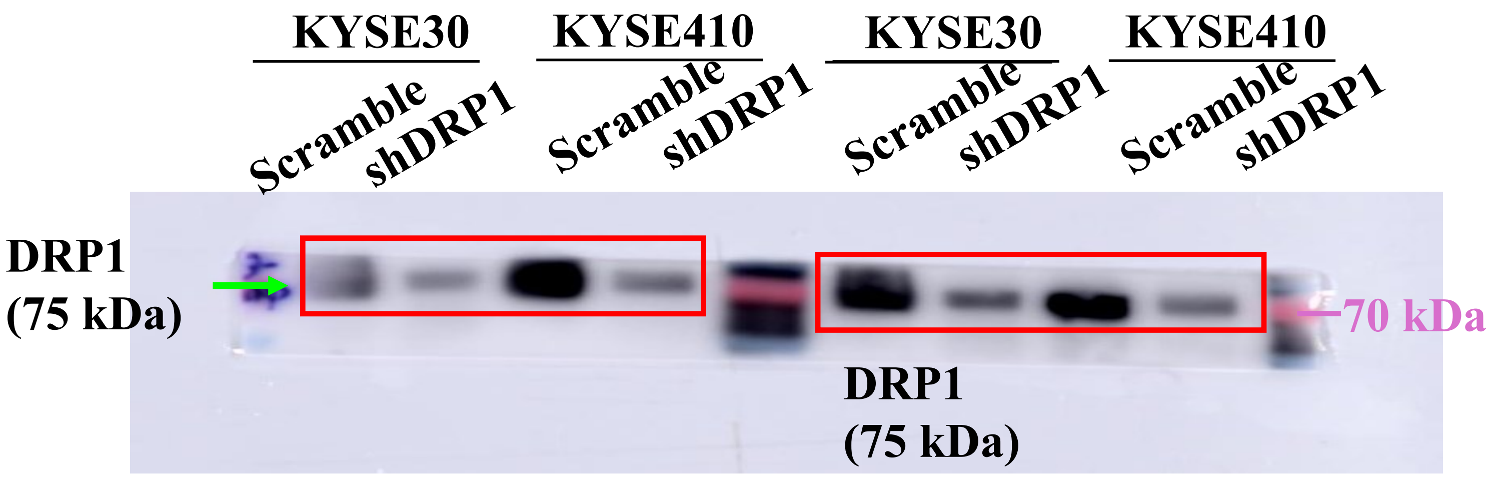

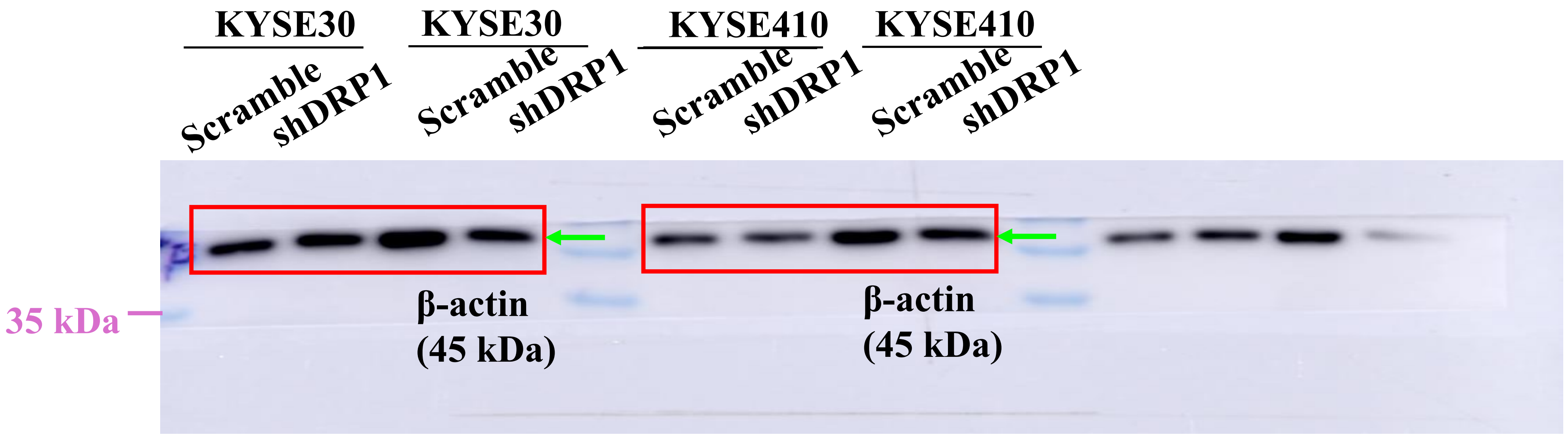

Supplementary Fig. 11: Complete uncropped western blot data of Fig. 2D

Replicate 2/3

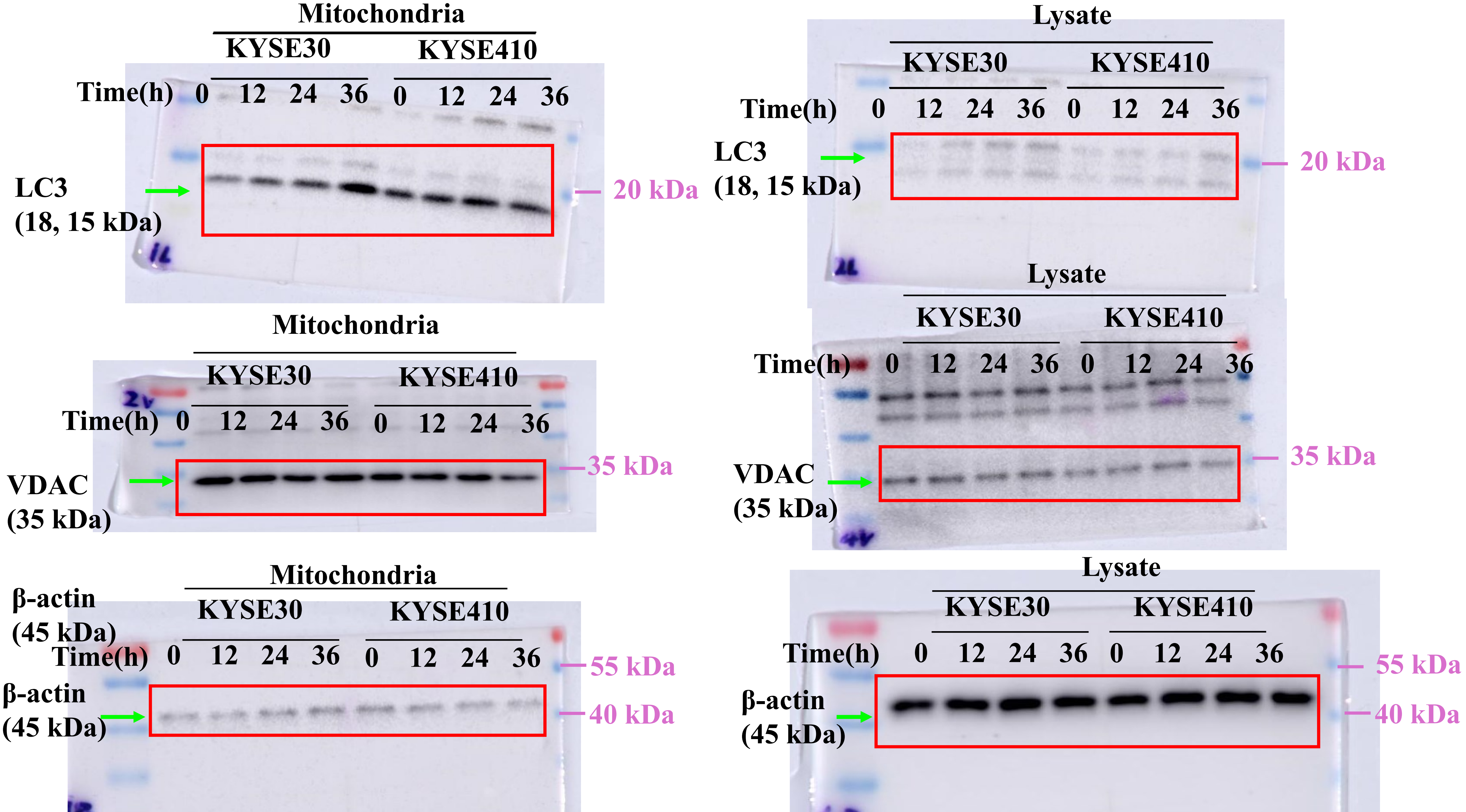

Supplementary Fig. 12: Complete uncropped western blot data of Fig. 3C

Used in Fig. 3C

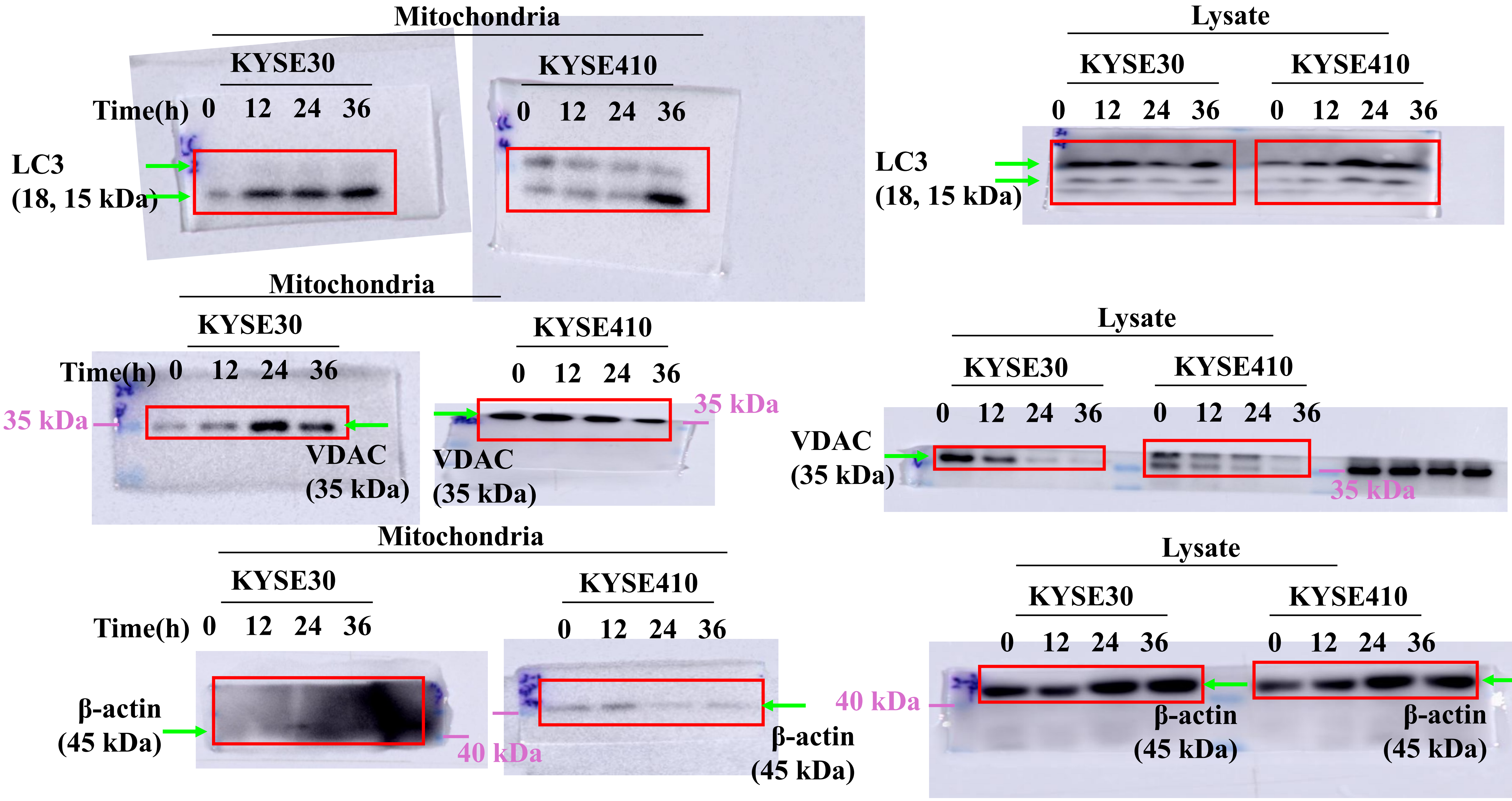

Supplementary Fig. 13: Complete uncropped western blot data of Fig. 3C **Replicate 2**

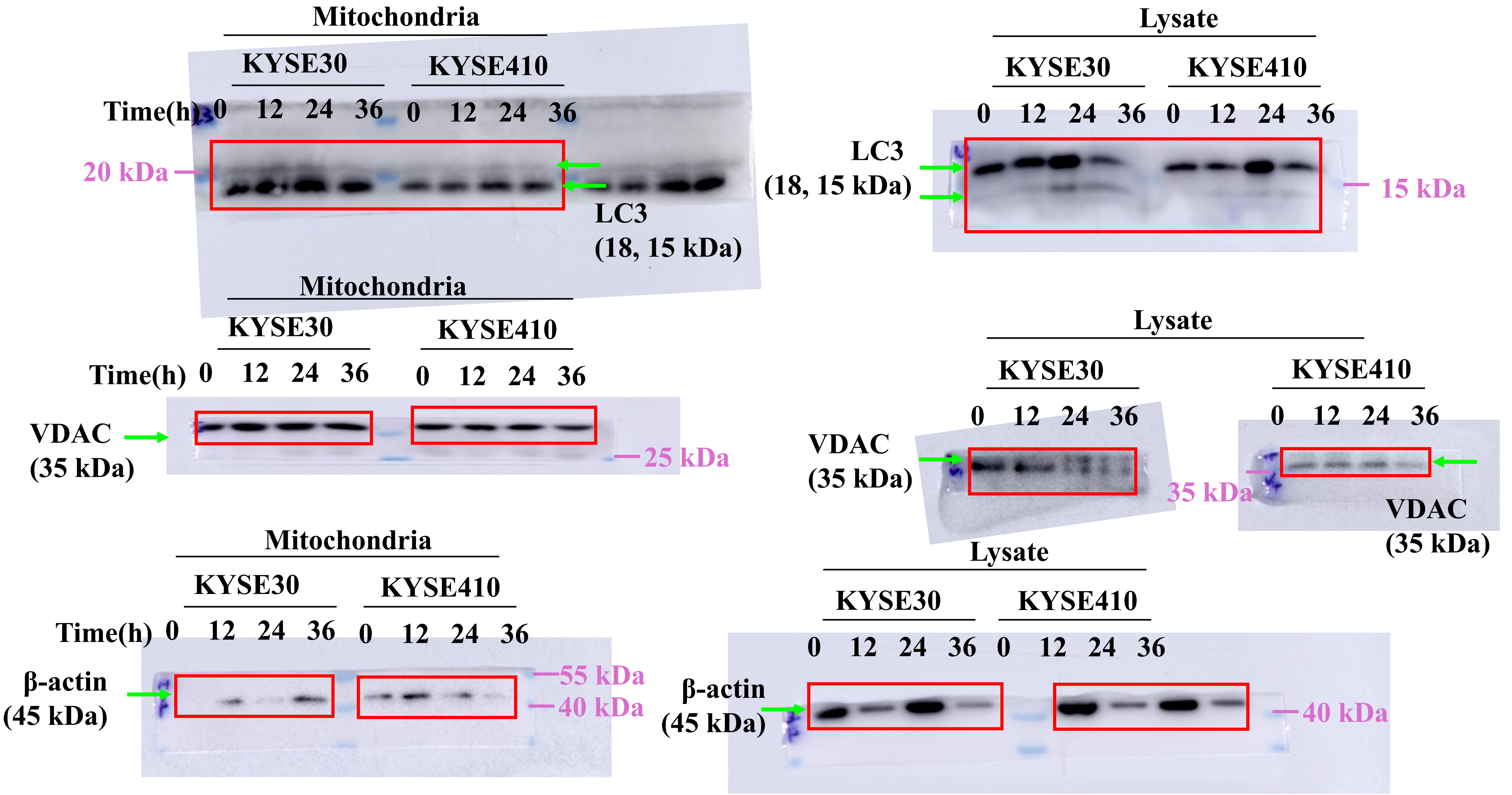

Supplementary Fig. 14: Complete uncropped western blot data of Fig. 3C

Replicate 3

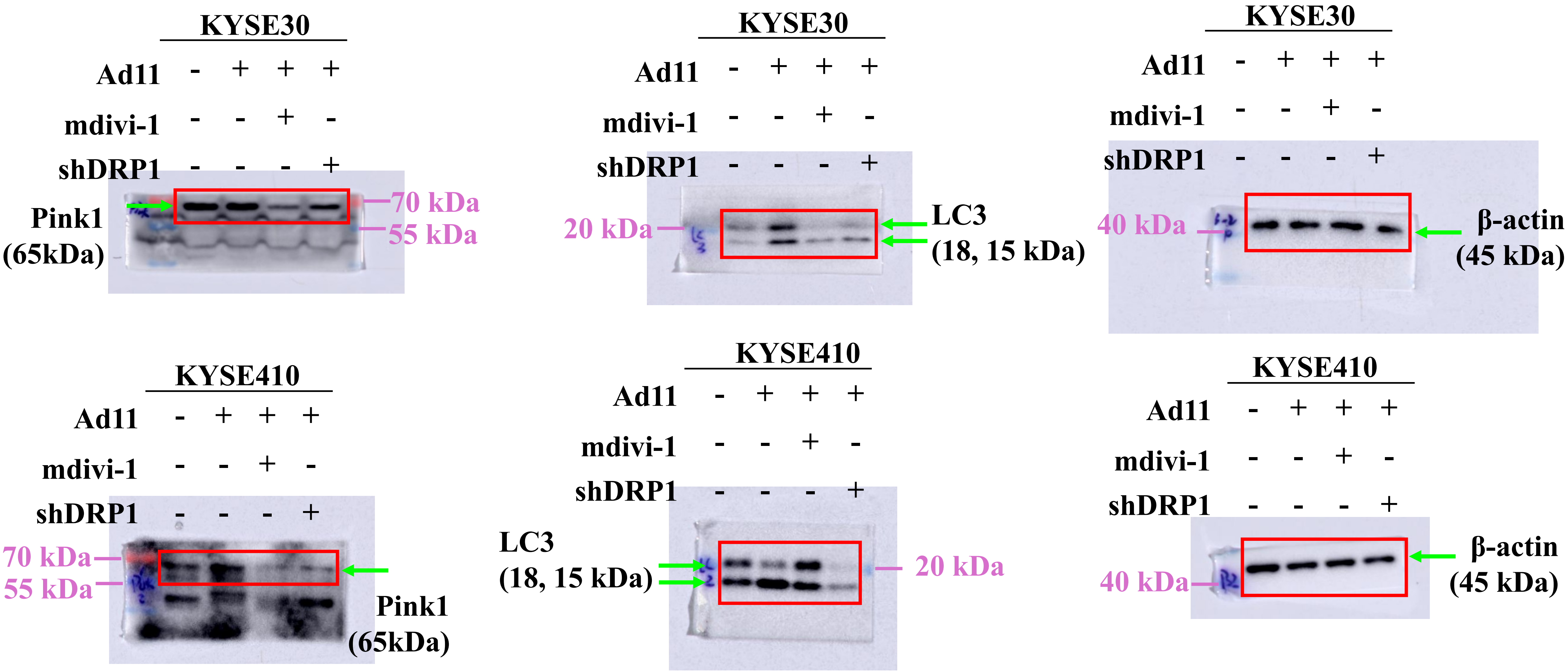

Supplementary Fig. 15: Complete uncropped western blot data of Fig. 3D **Used in Fig. 3D**

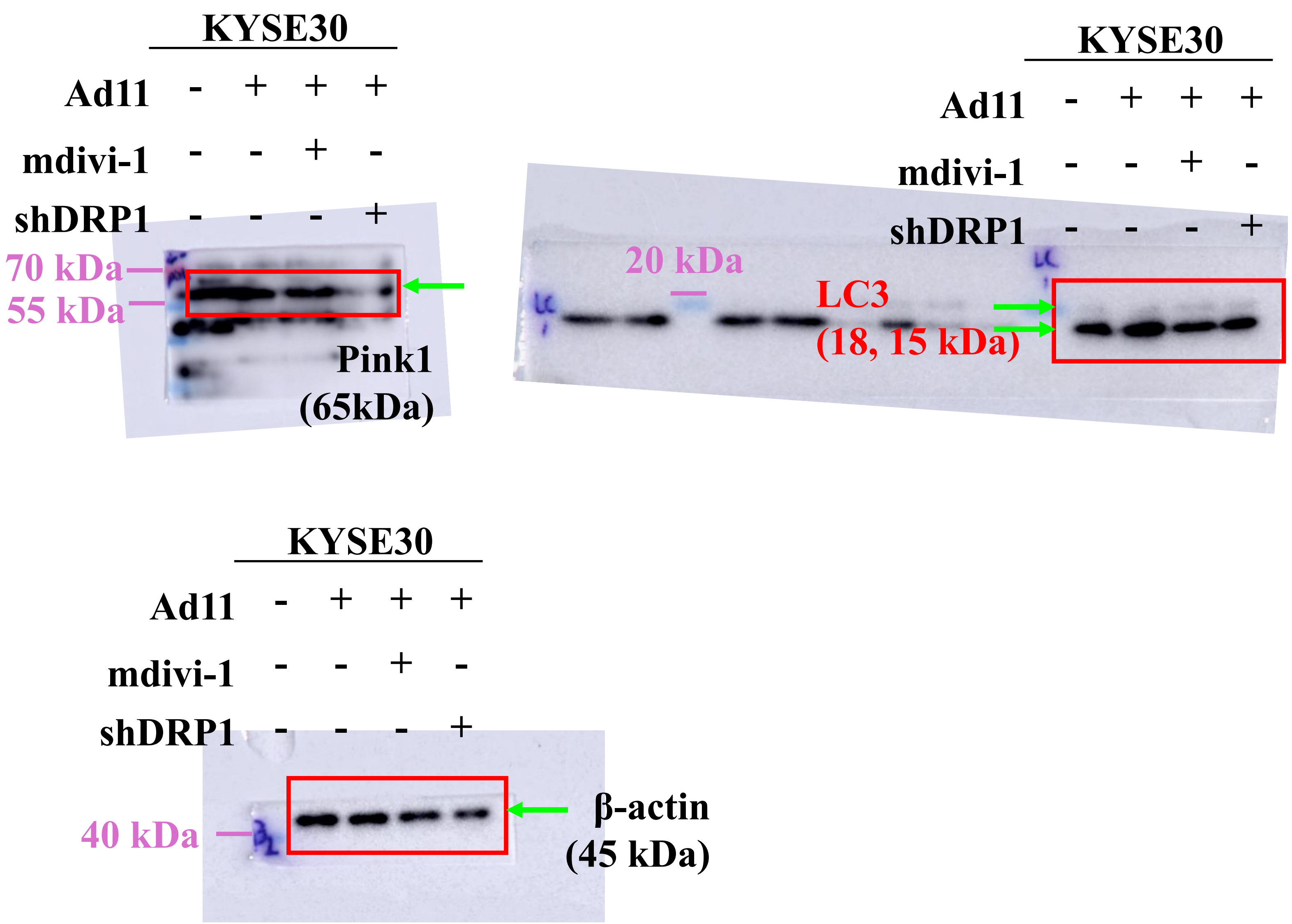

Supplementary Fig. 16: Complete uncropped western blot data of Fig. 3D

Replicate 2

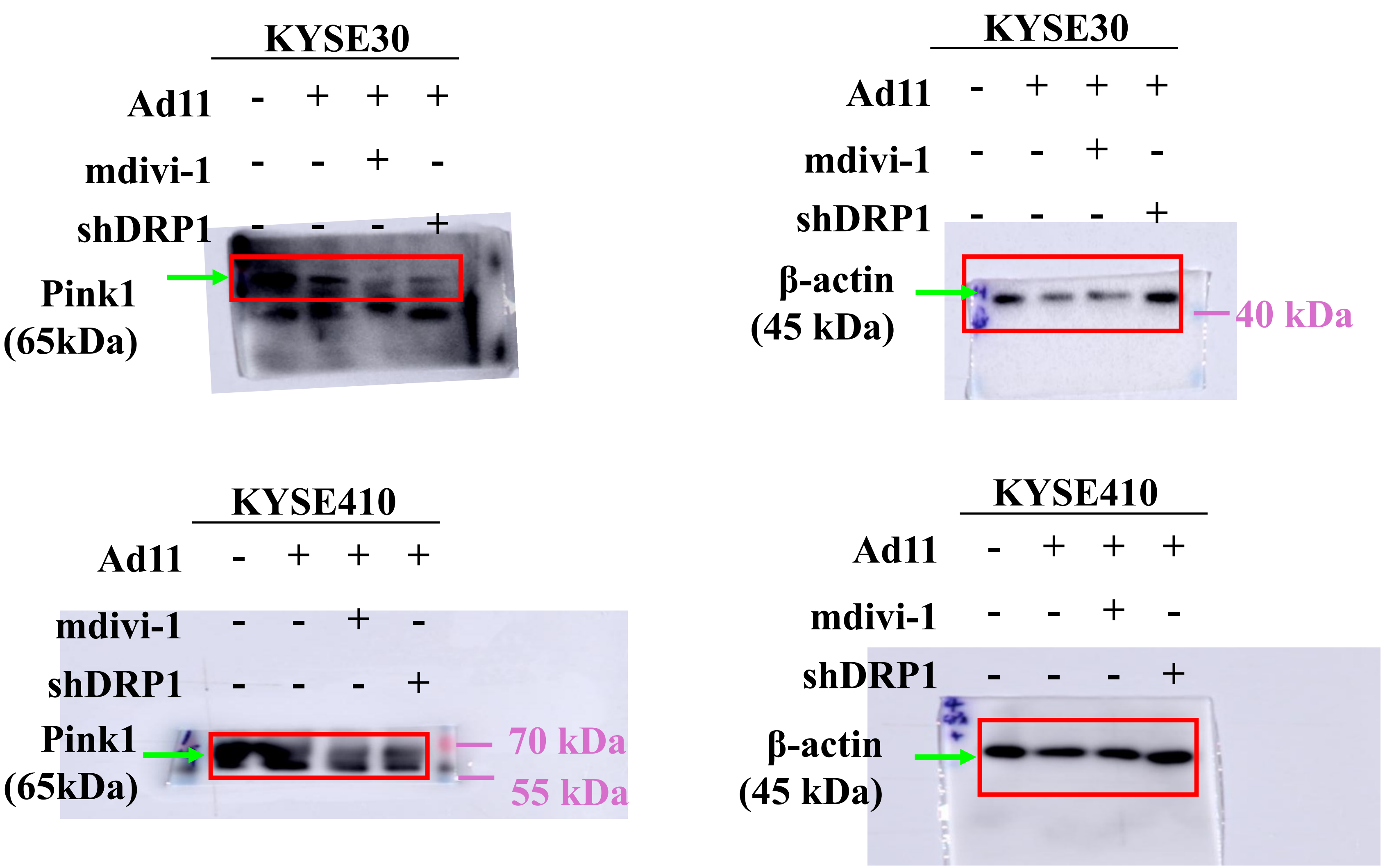

Supplementary Fig. 17: Complete uncropped western blot data of Fig. 3D **Replicate 3**

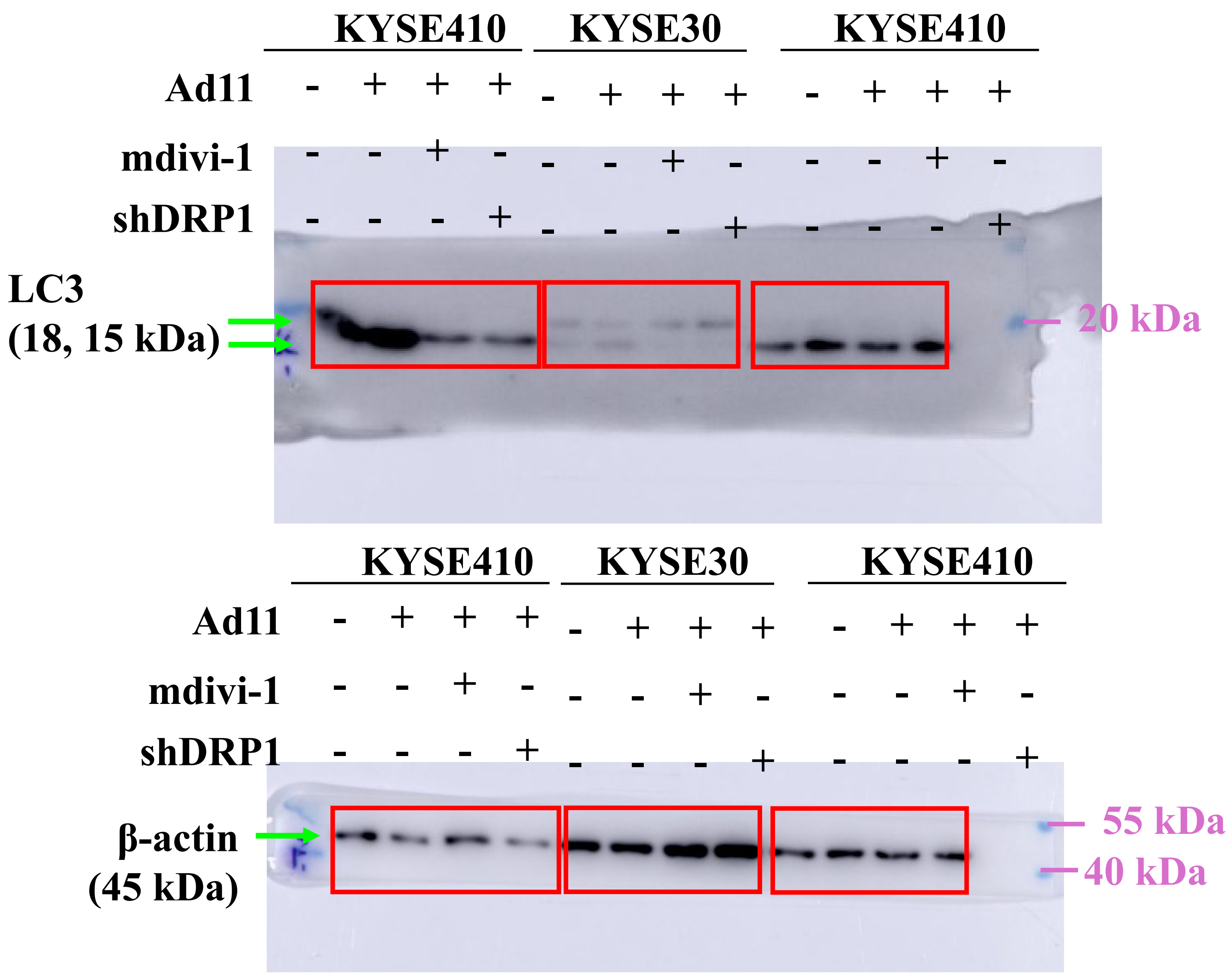

Supplementary Fig. 18: Complete uncropped western blot data of Fig. 3D

Replicate 3

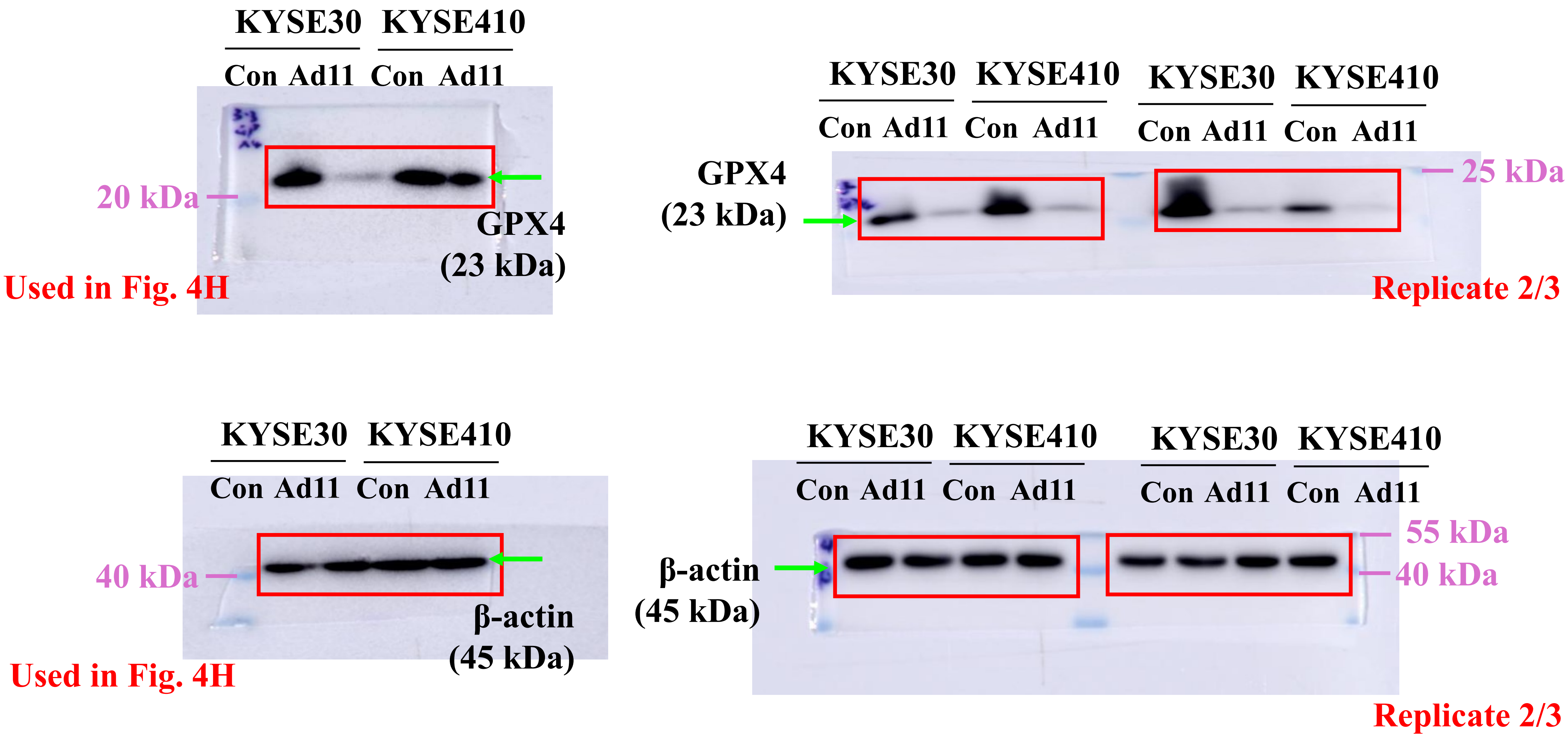

Supplementary Fig. 19: Complete uncropped western blot data of Fig. 4H

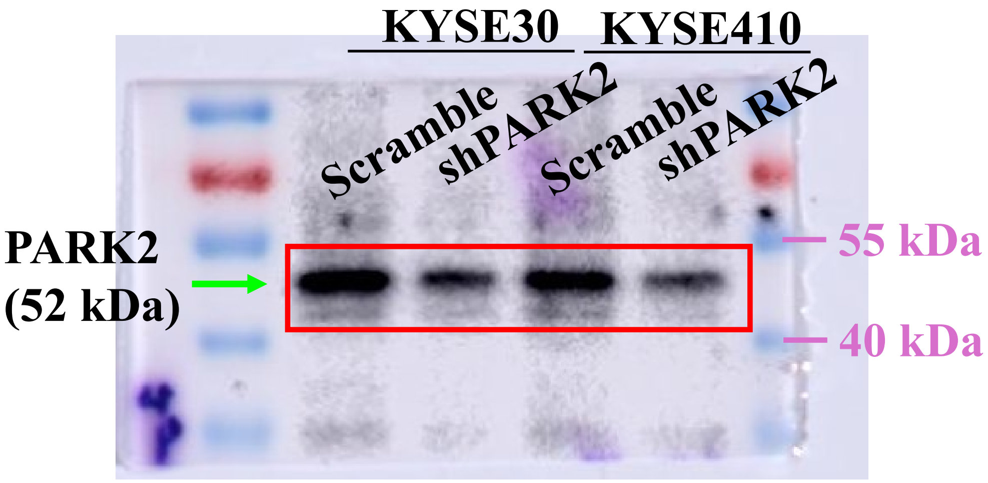

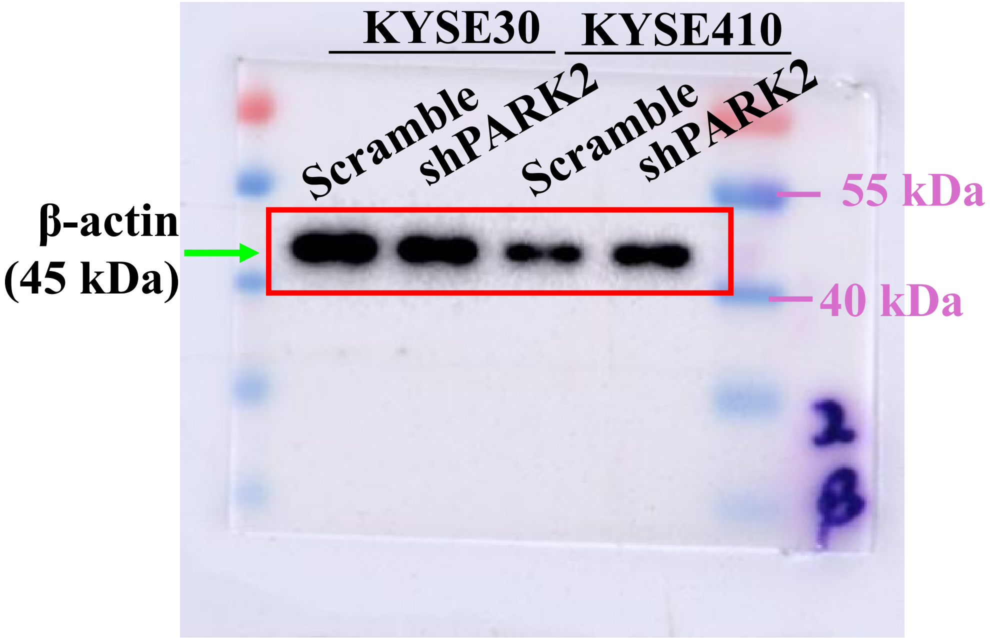

Supplementary Fig. 20: Complete uncropped western blot data of Fig. 5D

Used in Fig. 5D

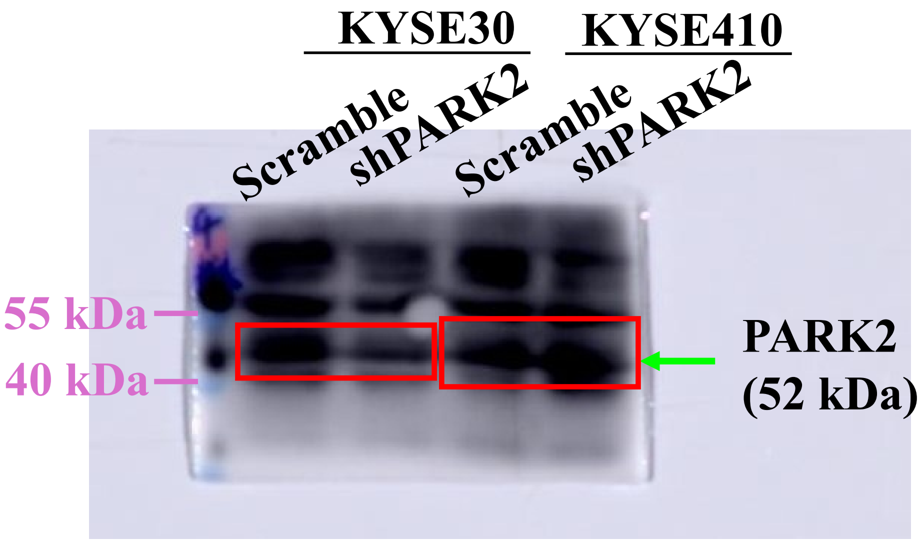

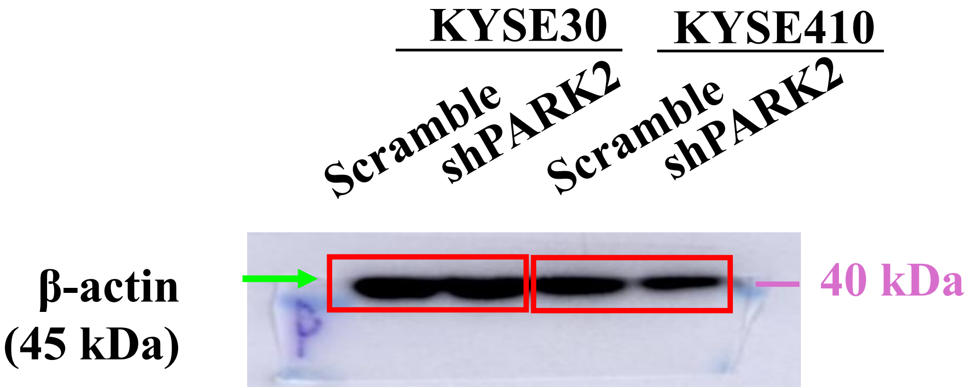

Supplementary Fig. 21: Complete uncropped western blot data of Fig. 5D

Replicate 2

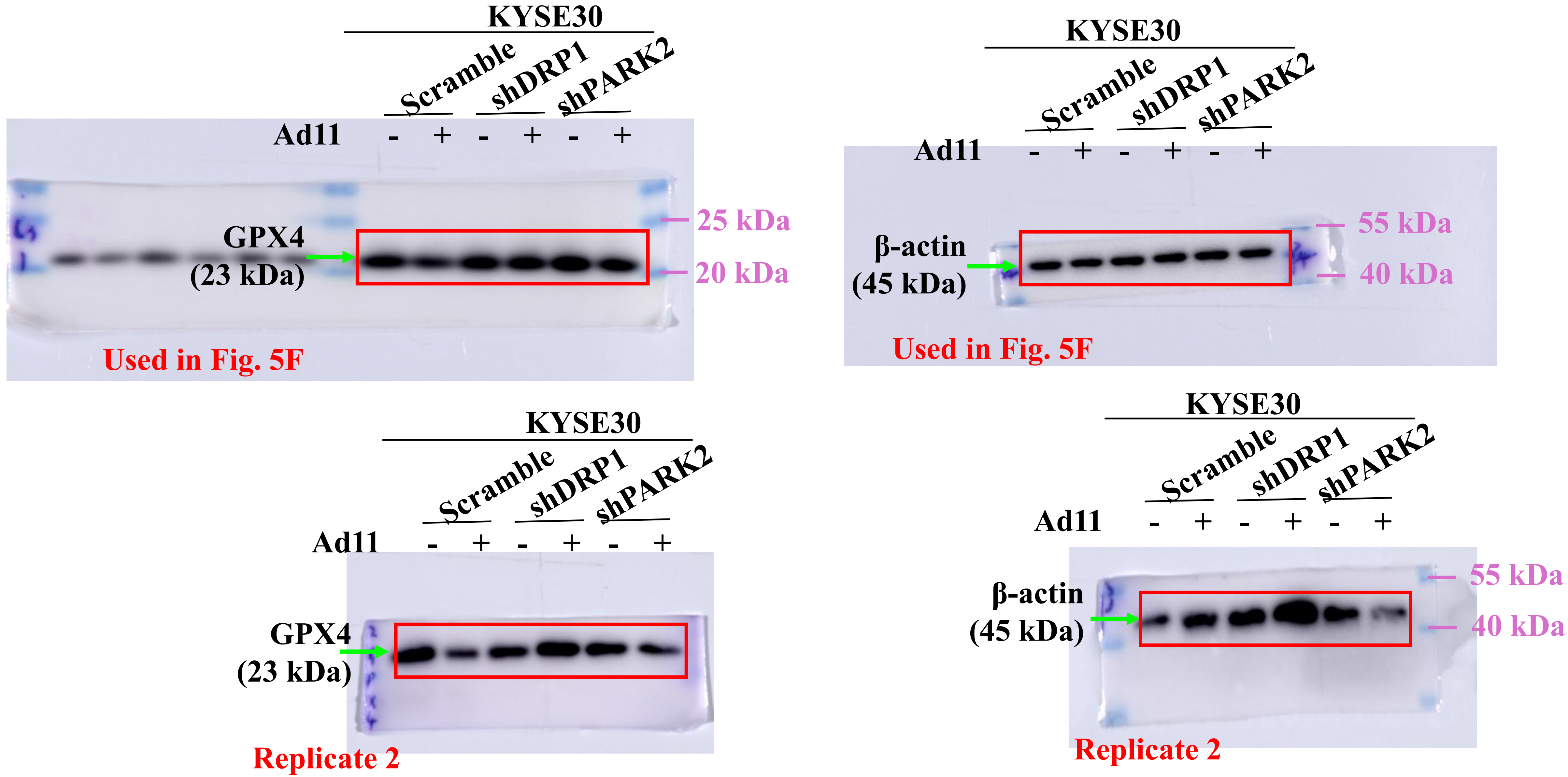

Supplementary Fig. 22: Complete uncropped western blot data of Fig. 5F

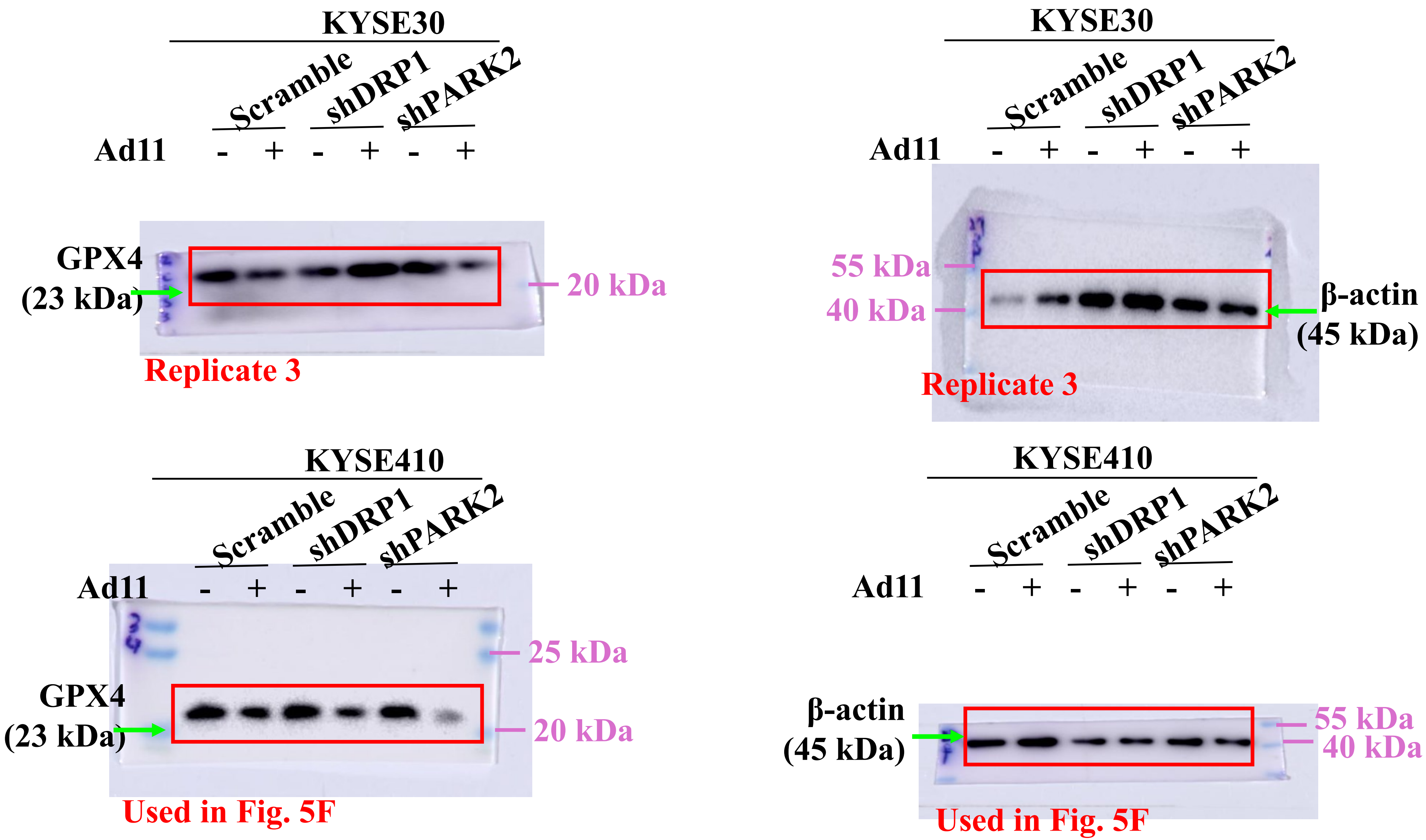

Supplementary Fig. 23: Complete uncropped western blot data of Fig. 5F

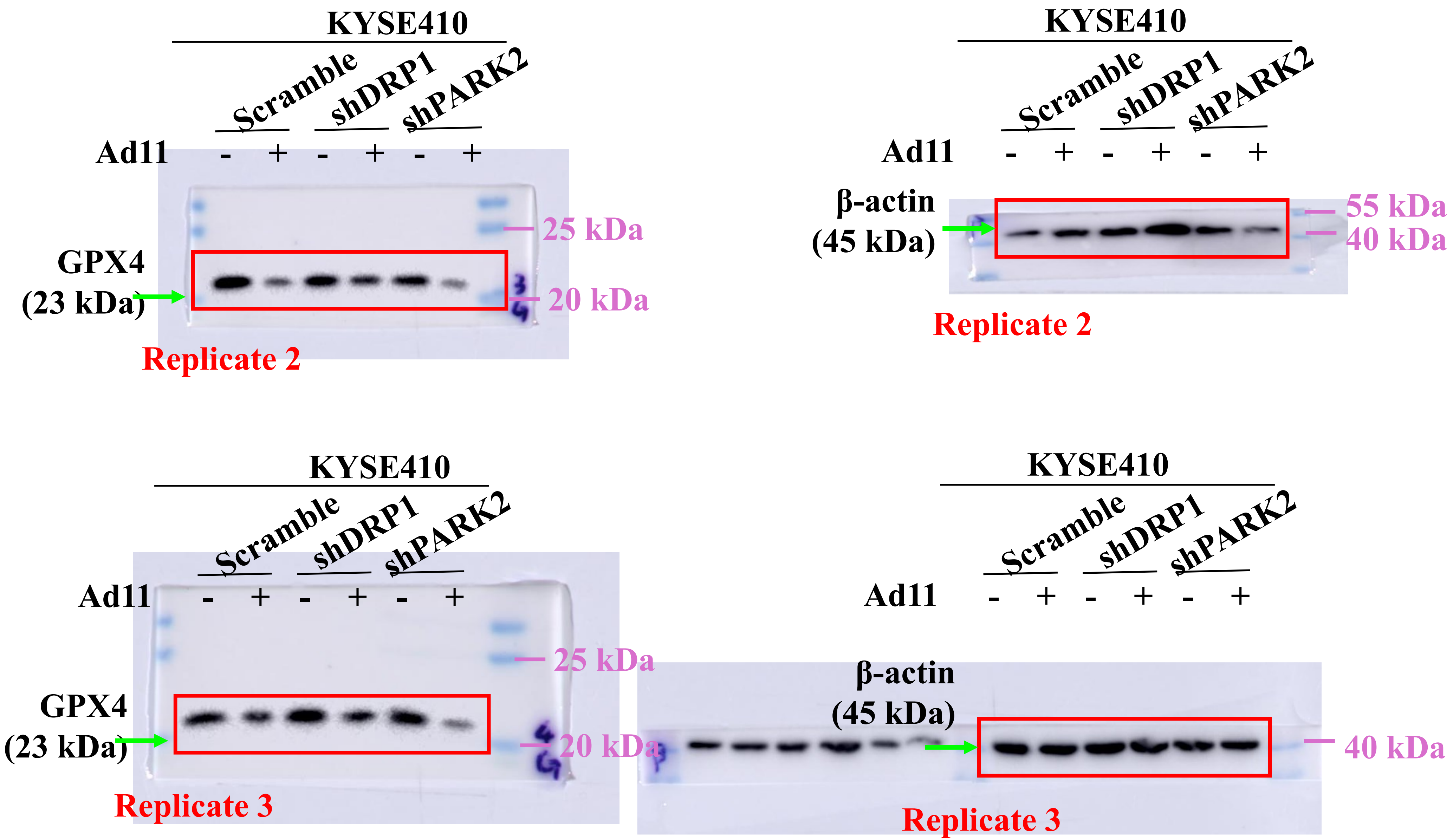

Supplementary Fig. 24: Complete uncropped western blot data of Fig. 5F

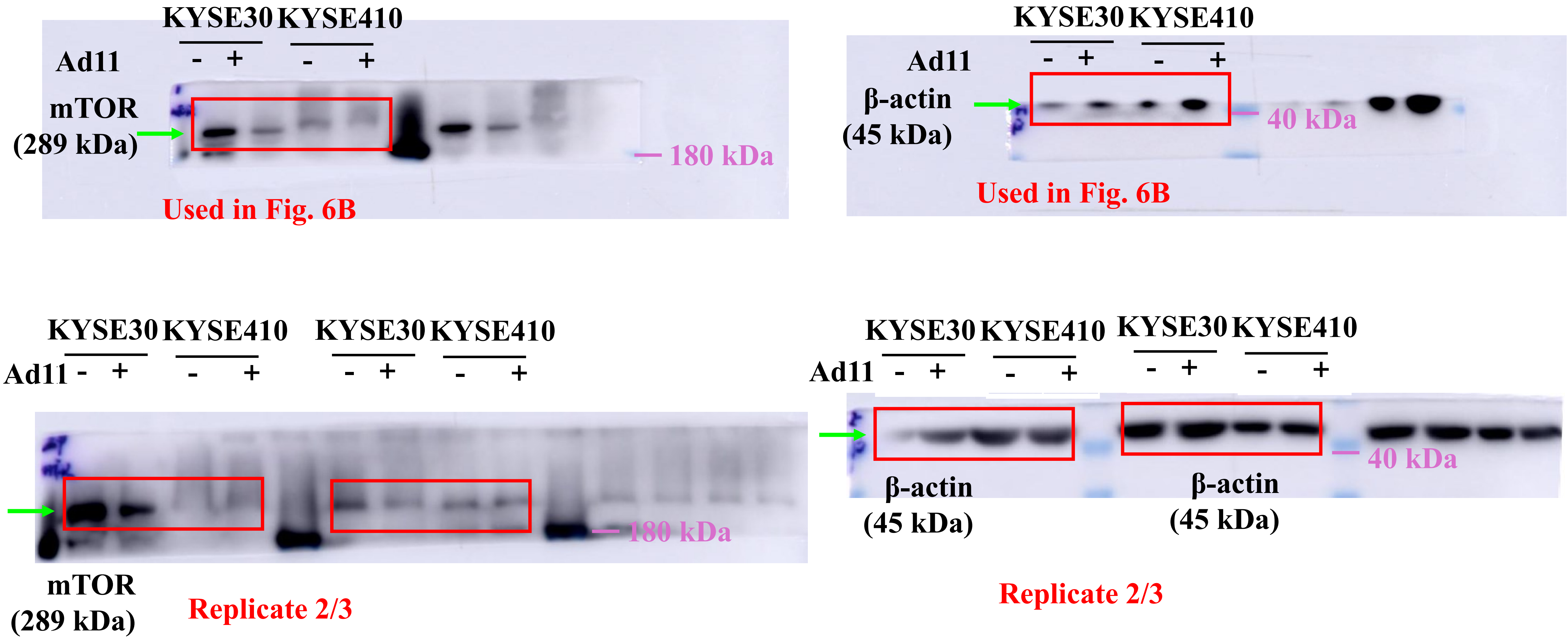

Supplementary Fig. 25: Complete uncropped western blot data of Fig. 6B

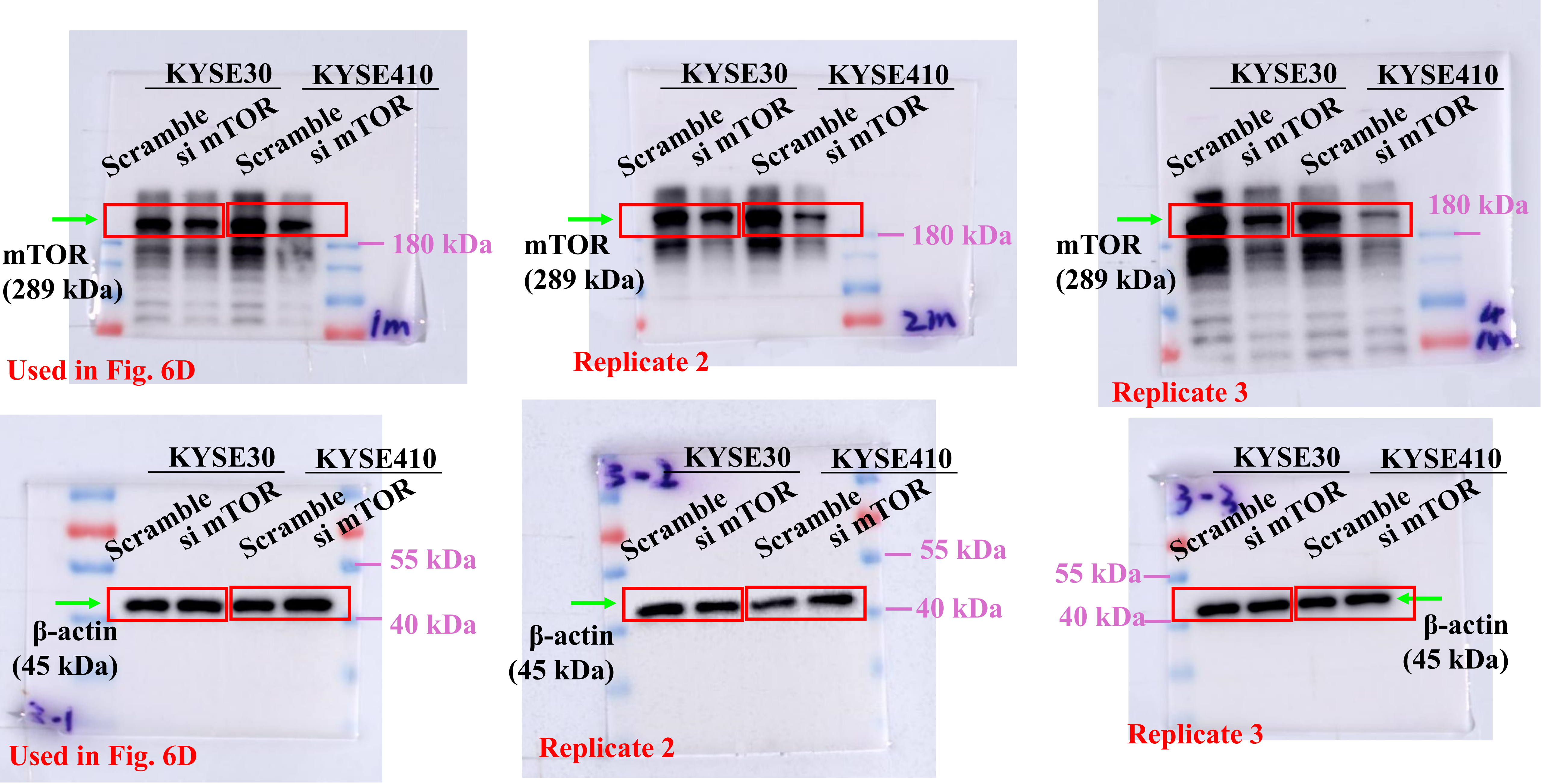

Supplementary Fig. 26: Complete uncropped western blot data of Fig.6D

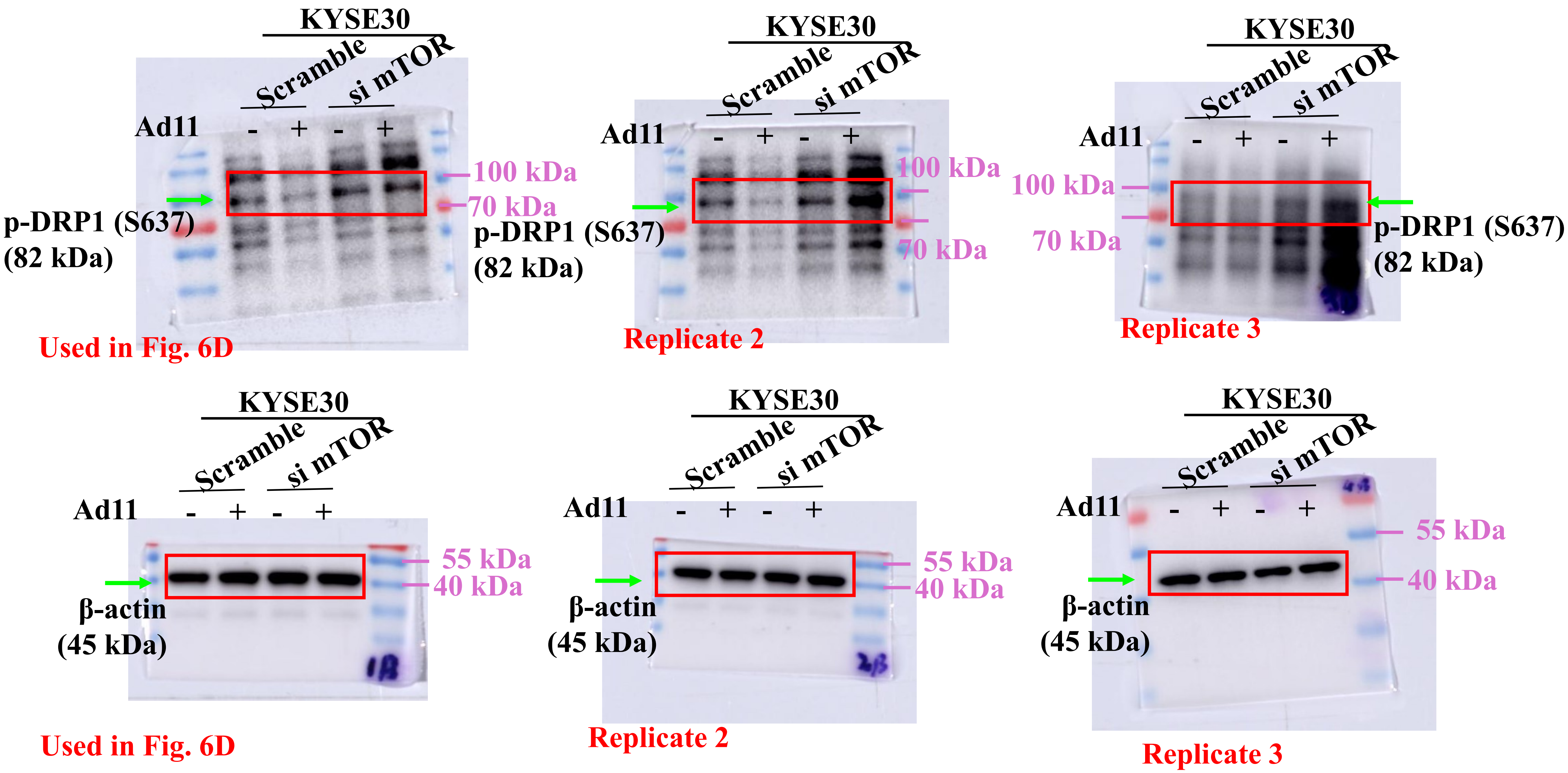

Supplementary Fig. 27: Complete uncropped western blot data of Fig.6D

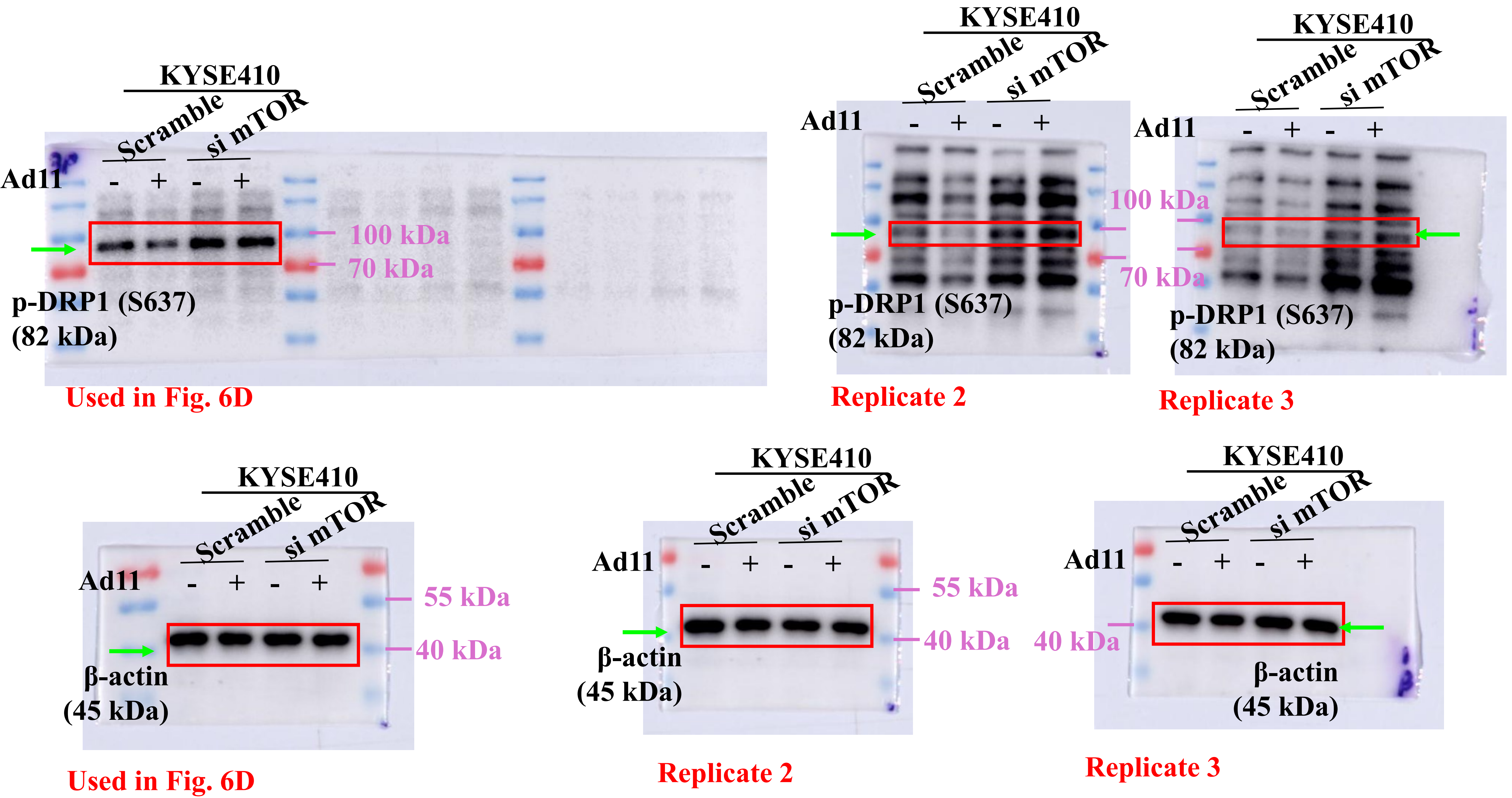

Supplementary Fig. 28: Complete uncropped western blot data of Fig.6D

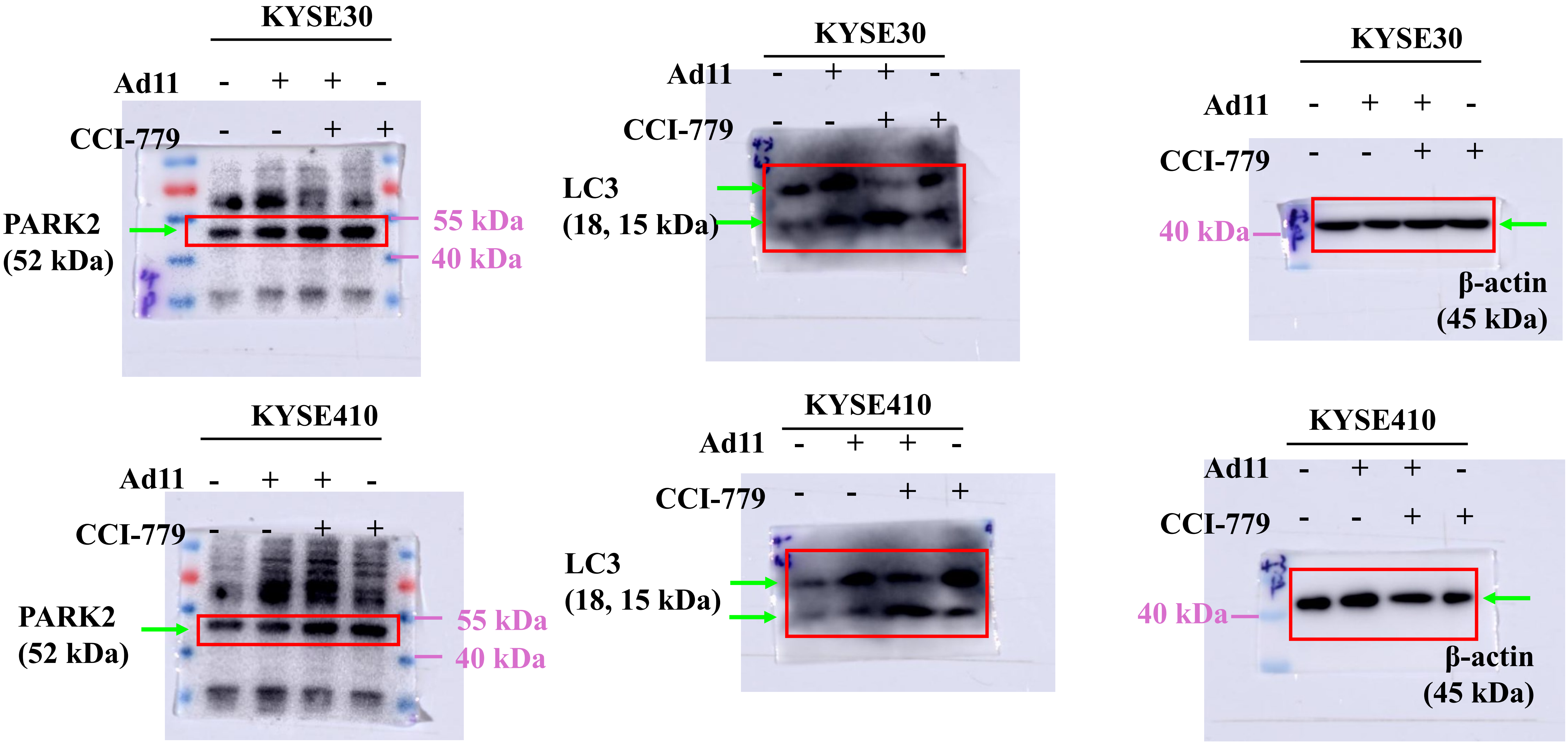

Supplementary Fig. 29: Complete uncropped western blot data of Fig. 6E

Used in Fig. 6E

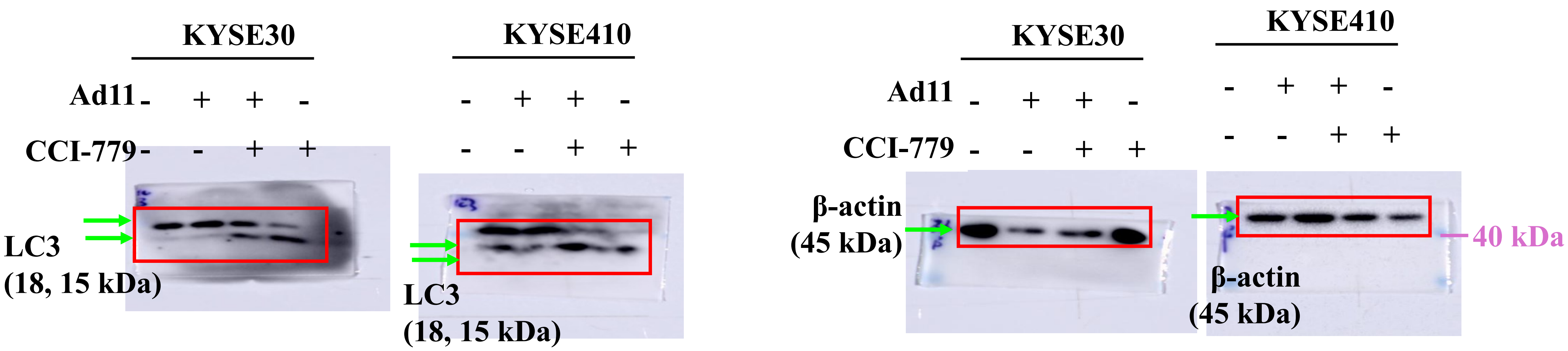

Supplementary Fig. 30: Complete uncropped western blot data of Fig. 6E

Replicate 2

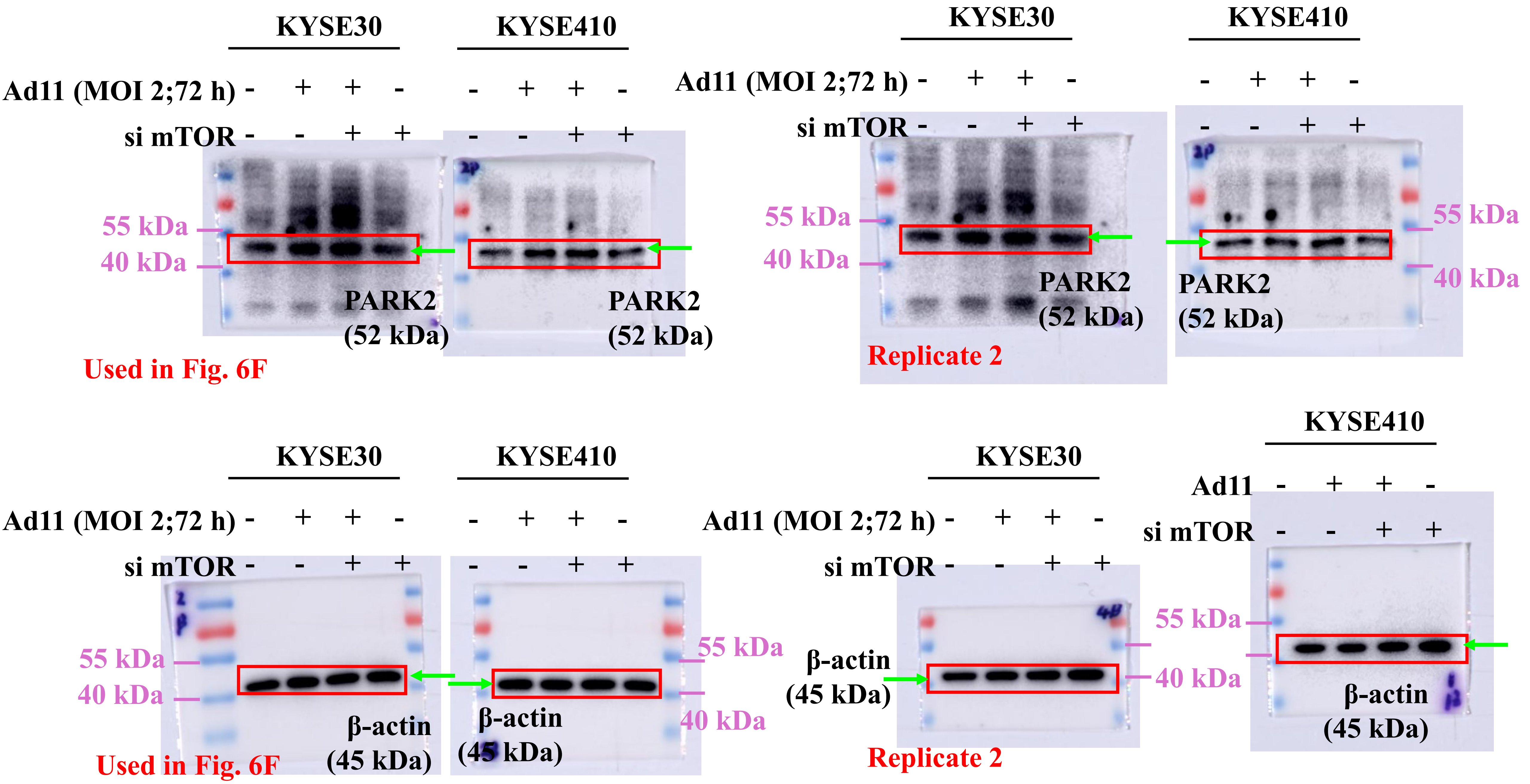

Supplementary Fig. 31: Complete uncropped western blot data of Fig.6F

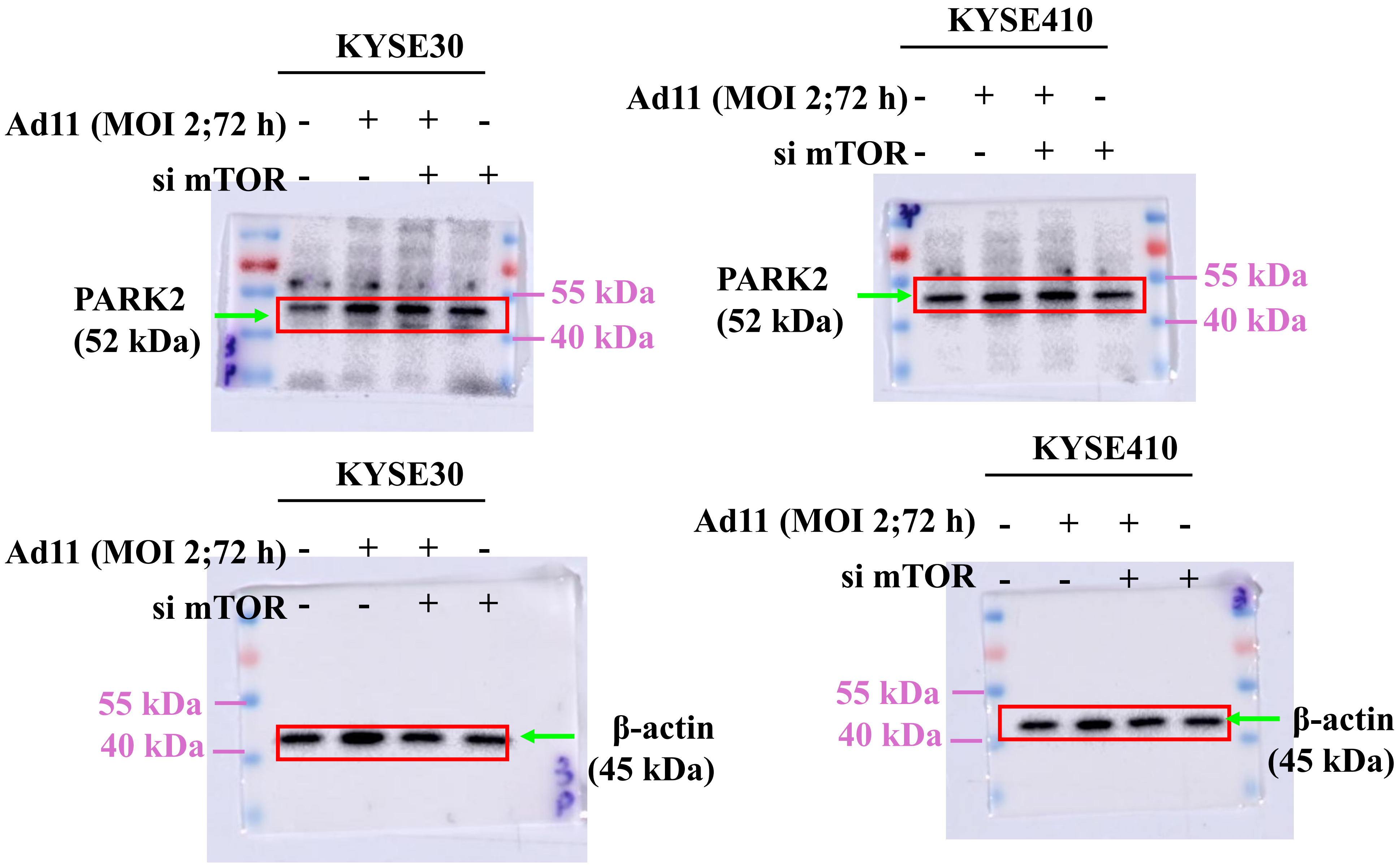

Supplementary Fig. 32: Complete uncropped western blot data of Fig.6F

Replicate 3

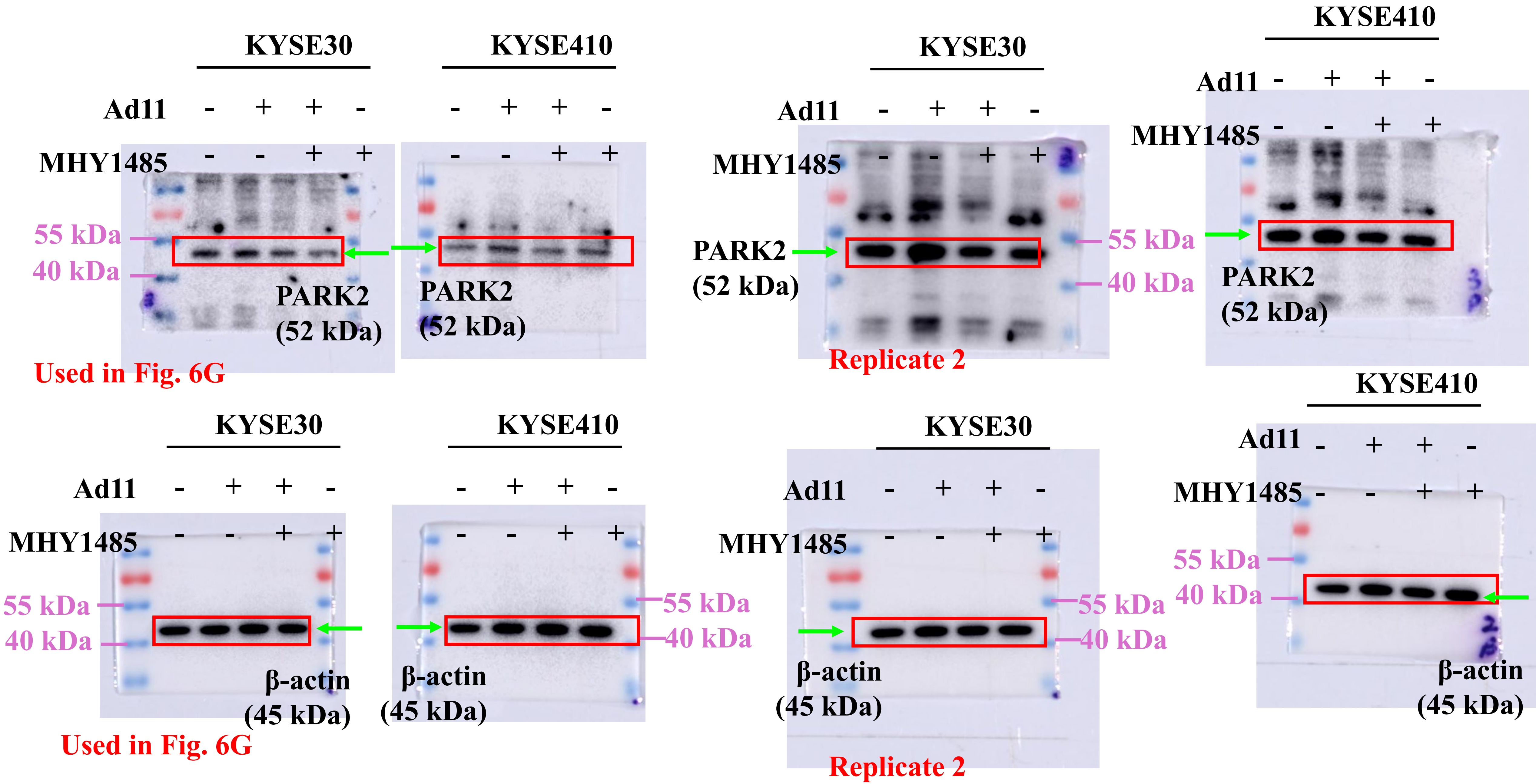

Supplementary Fig. 33: Complete uncropped western blot data of Fig.6G

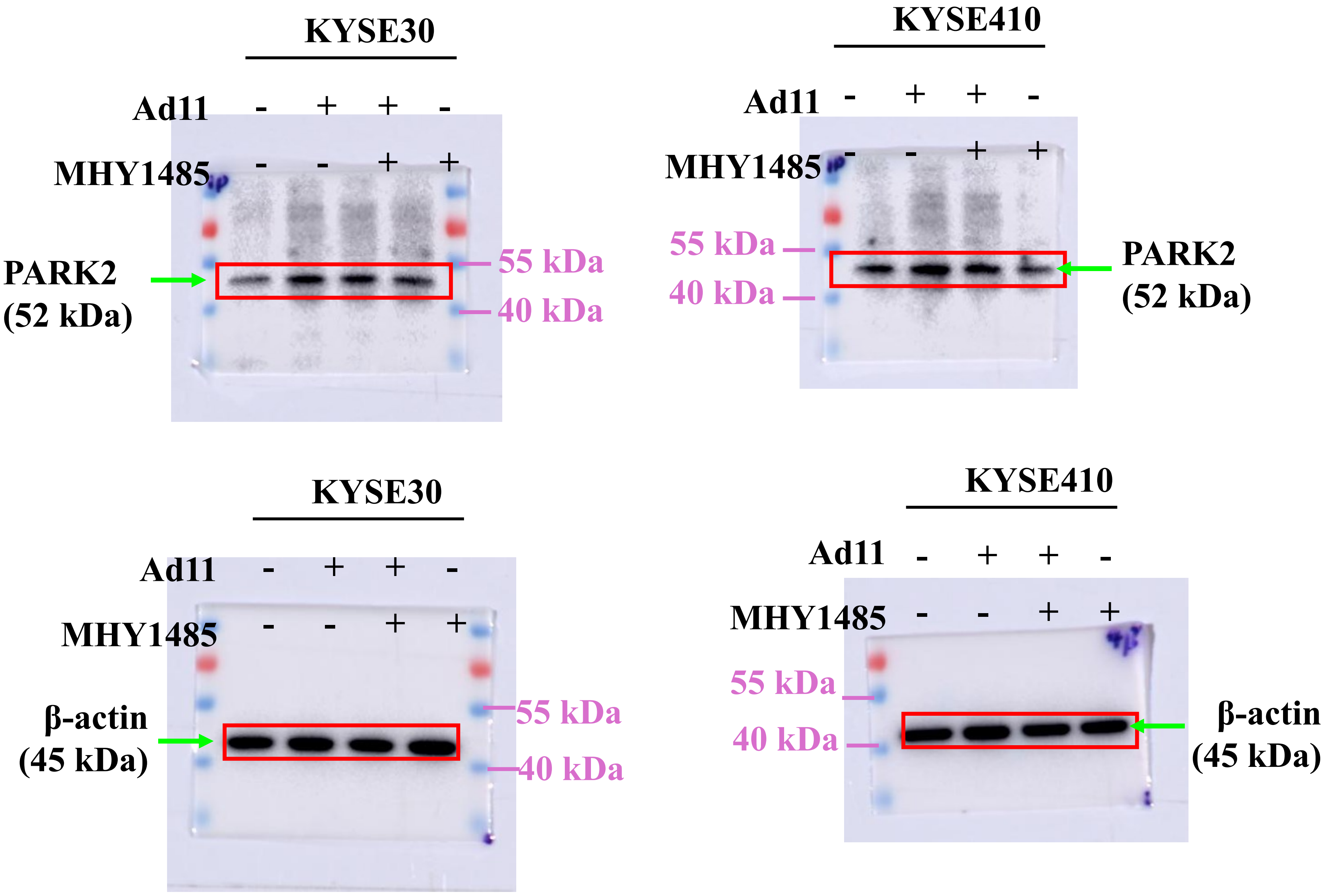

Supplementary Fig. 34: Complete uncropped western blot data of Fig.6G

Replicate 3

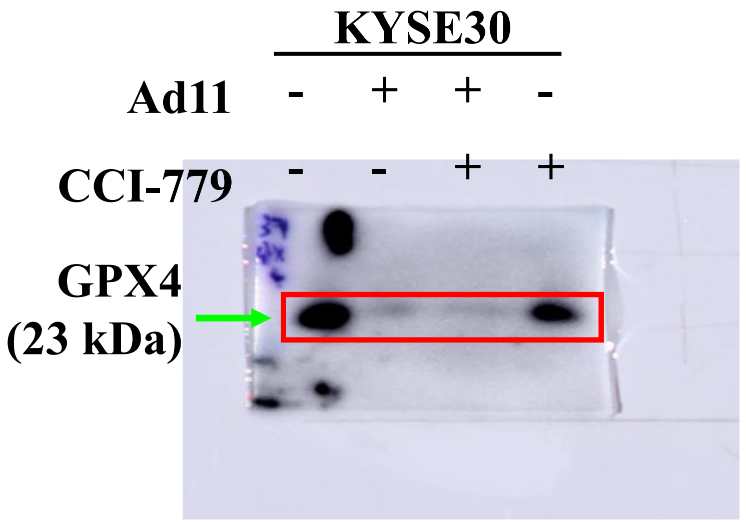

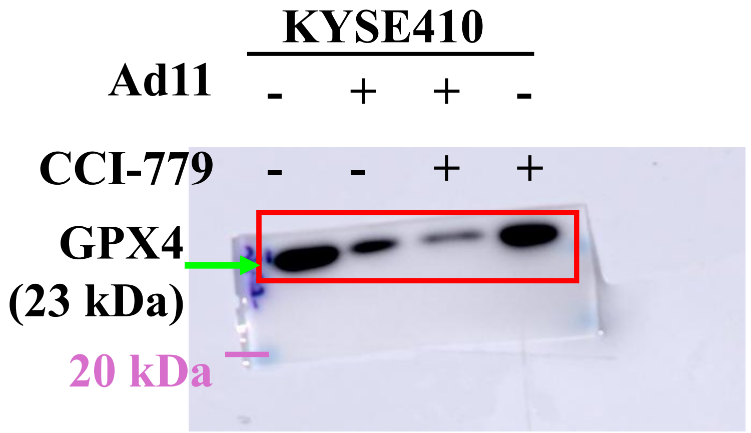

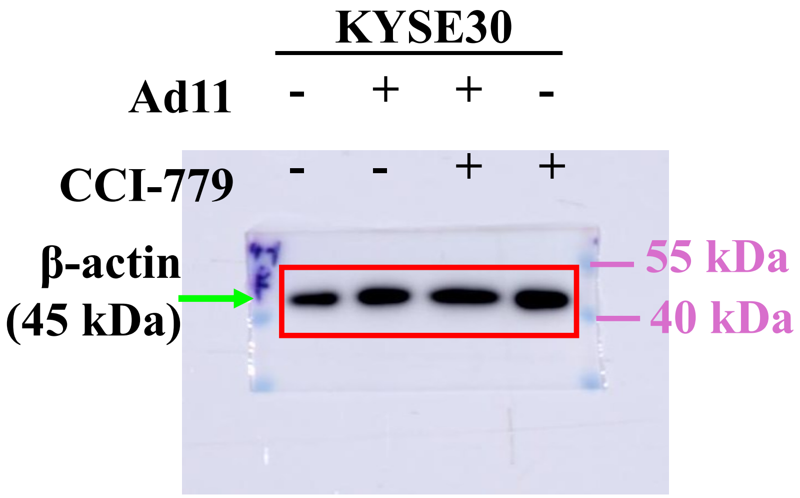

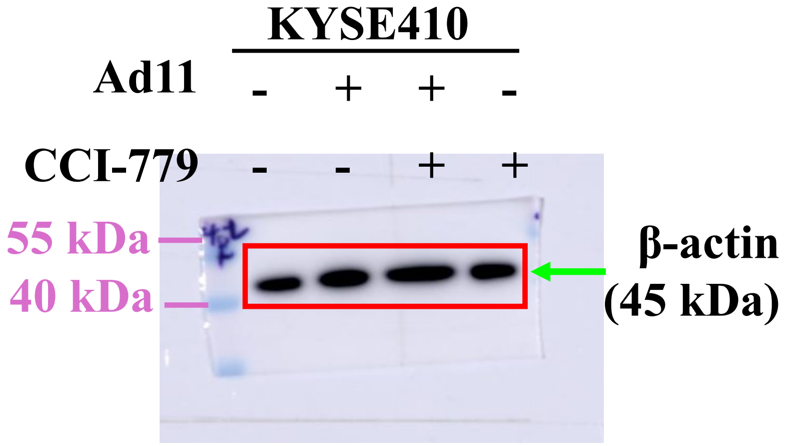

Supplementary Fig. 35: Complete uncropped western blot data of Fig. 6I

Used in Fig. 6I

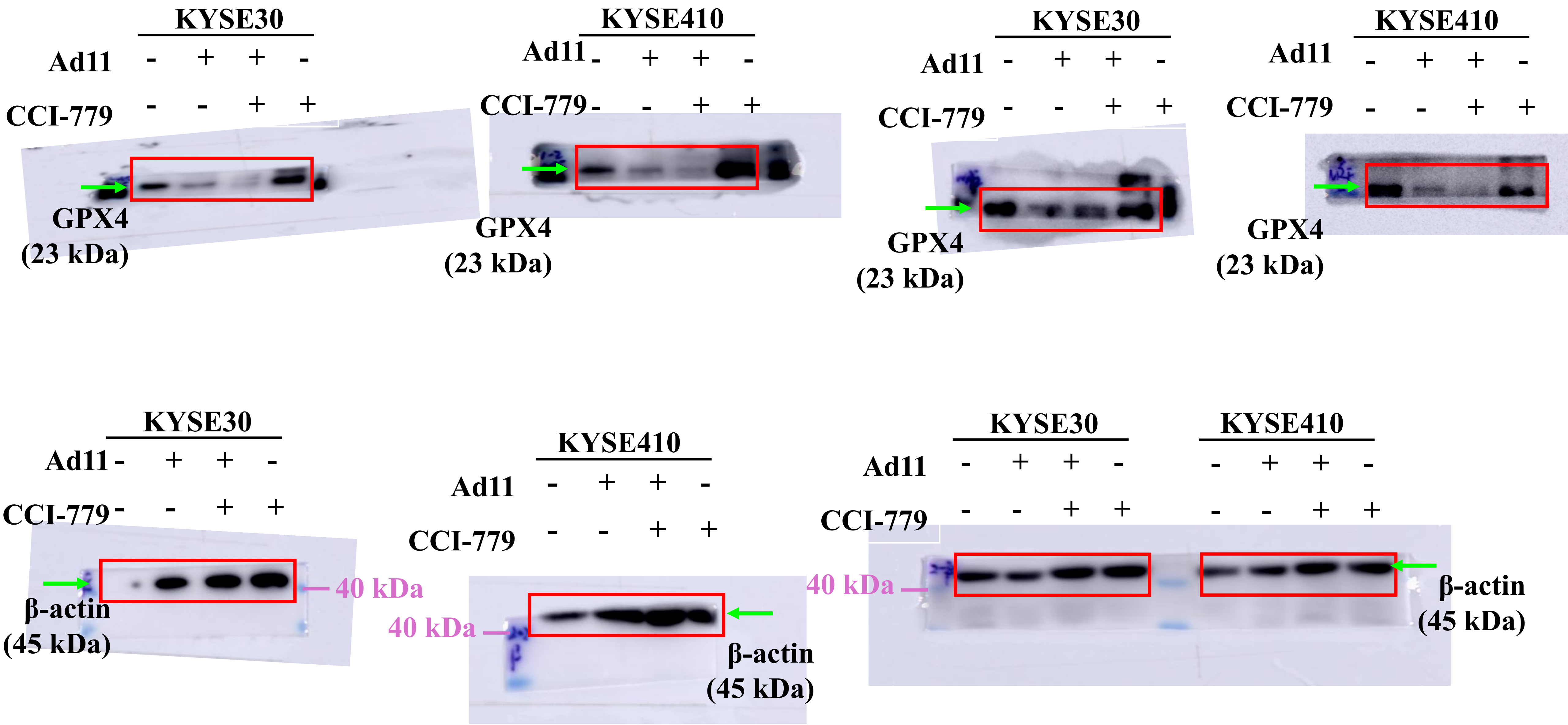

Supplementary Fig. 36: Complete uncropped western blot data of Fig. 6I

Replicate 2/3

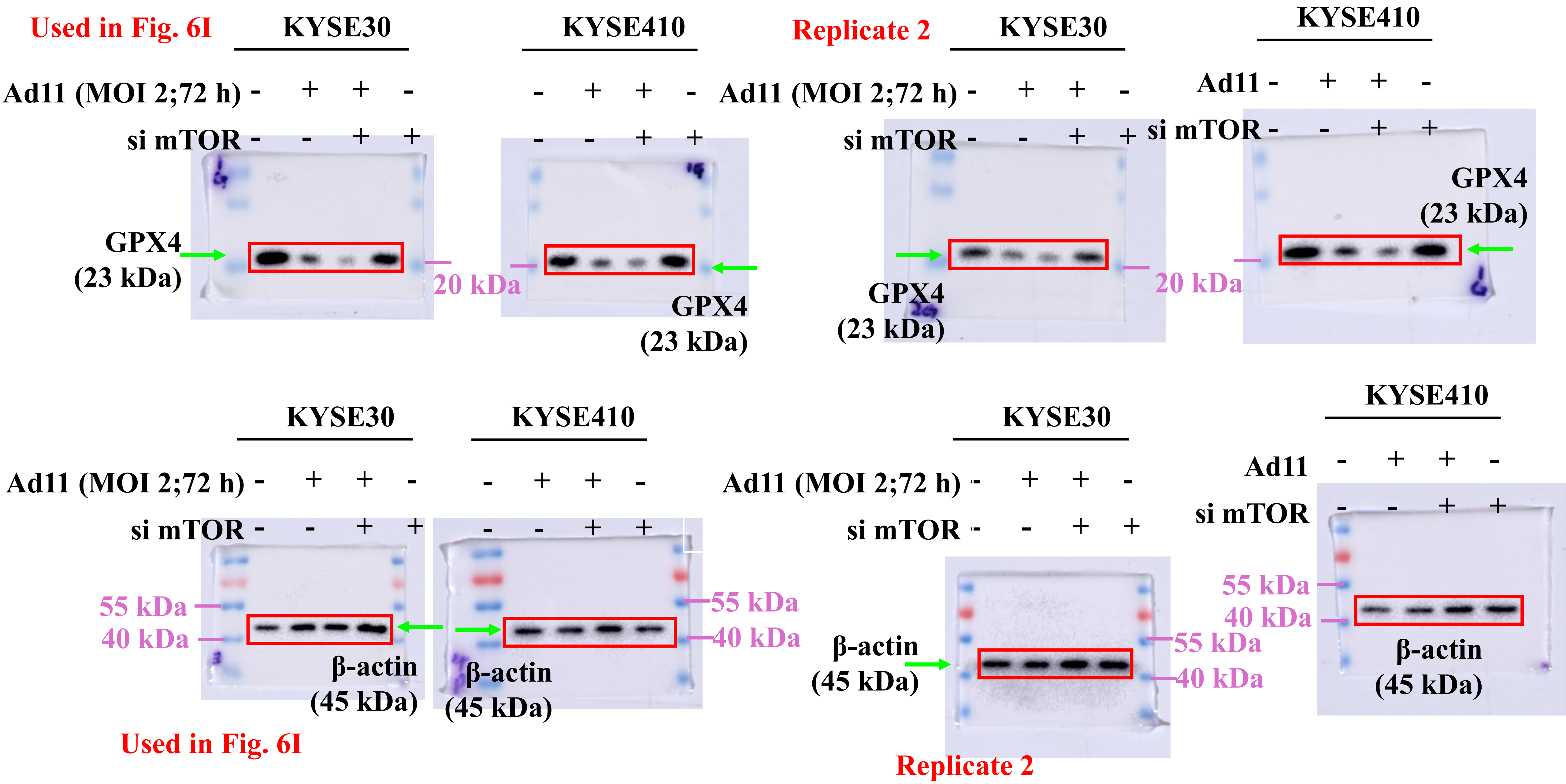

Supplementary Fig. 37: Complete uncropped western blot data of Fig. 6I

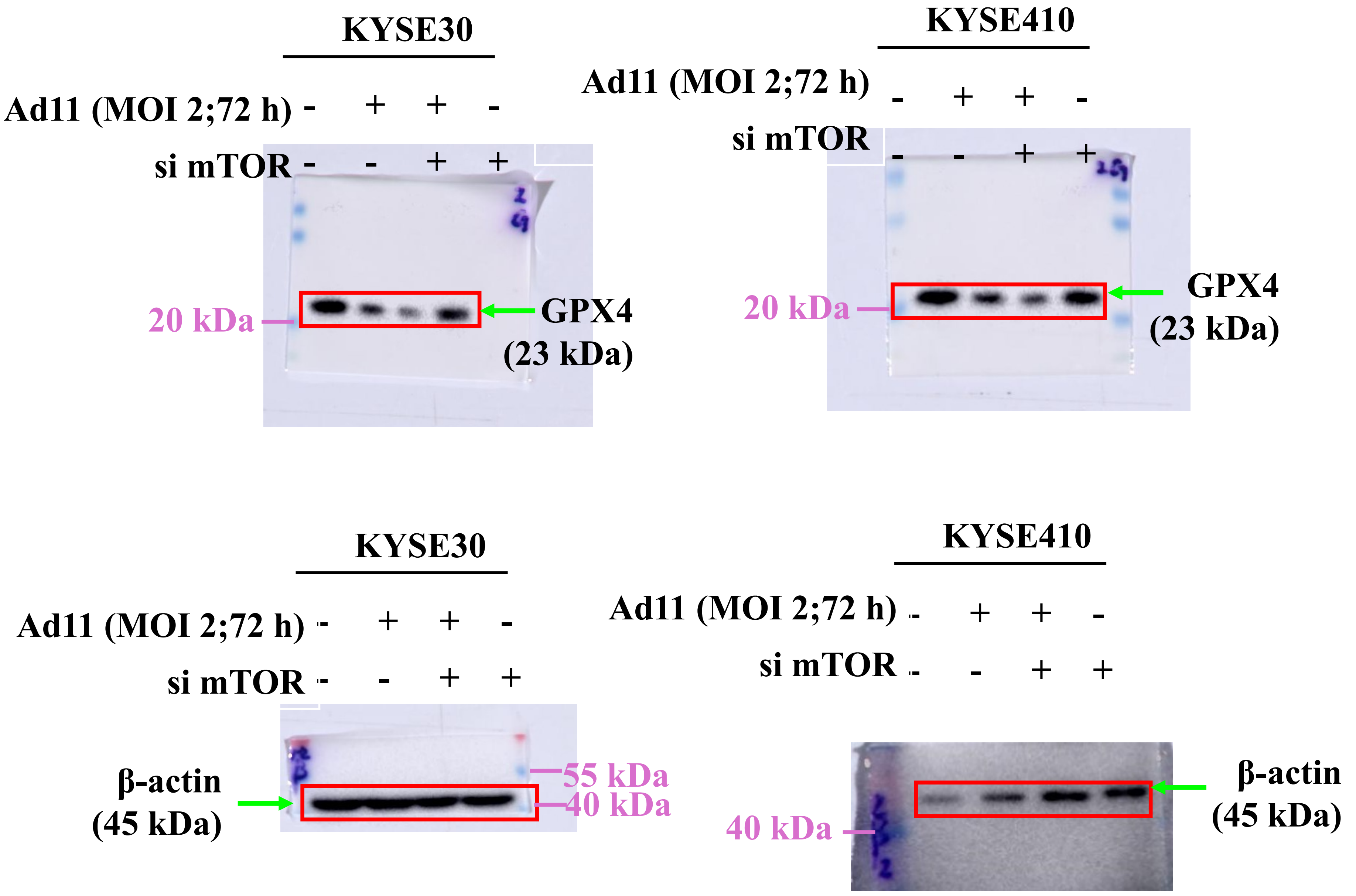

Supplementary Fig. 38: Complete uncropped western blot data of Fig. 6I

Replicate 3

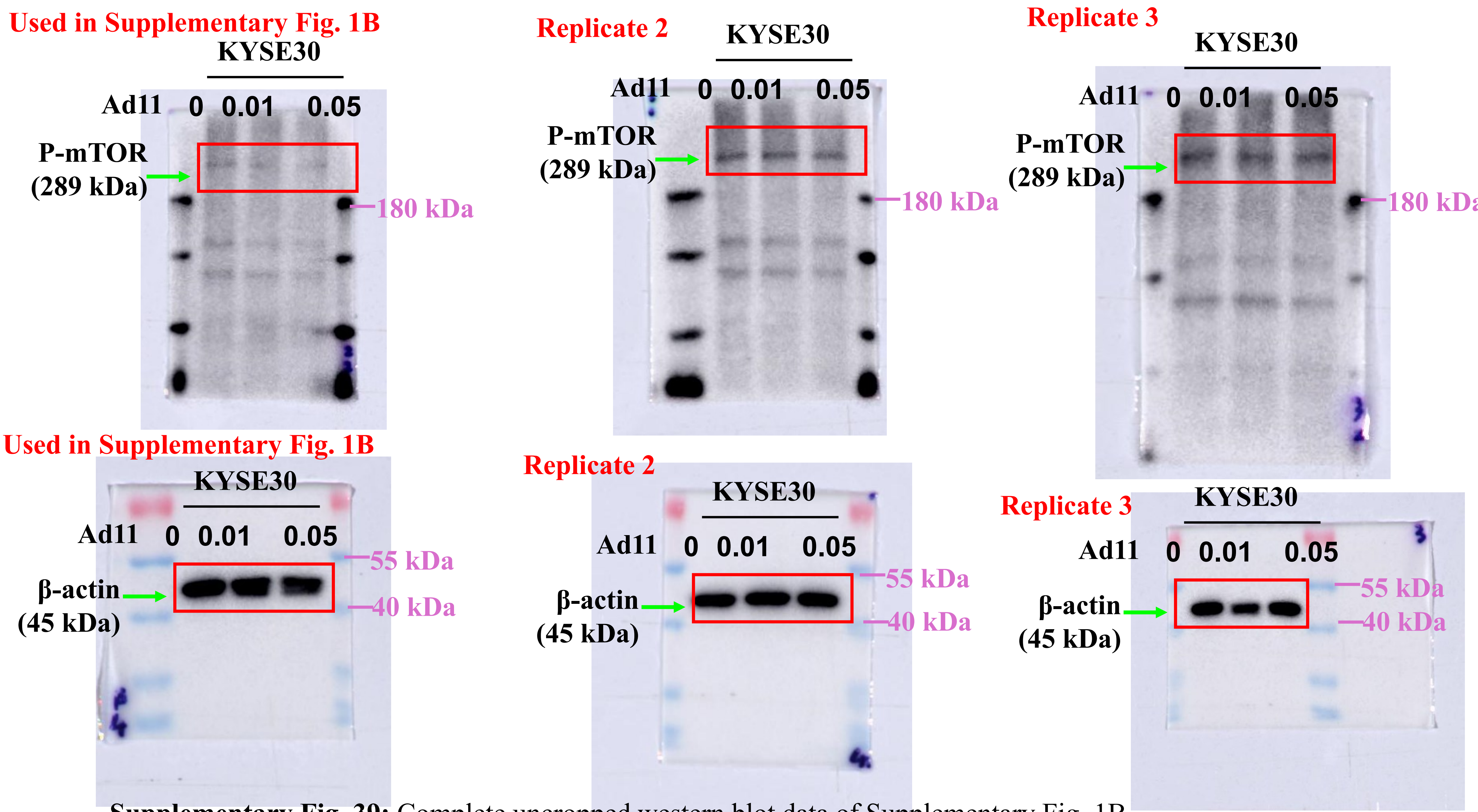

Supplementary Fig. 39: Complete uncropped western blot data of Supplementary Fig. 1B

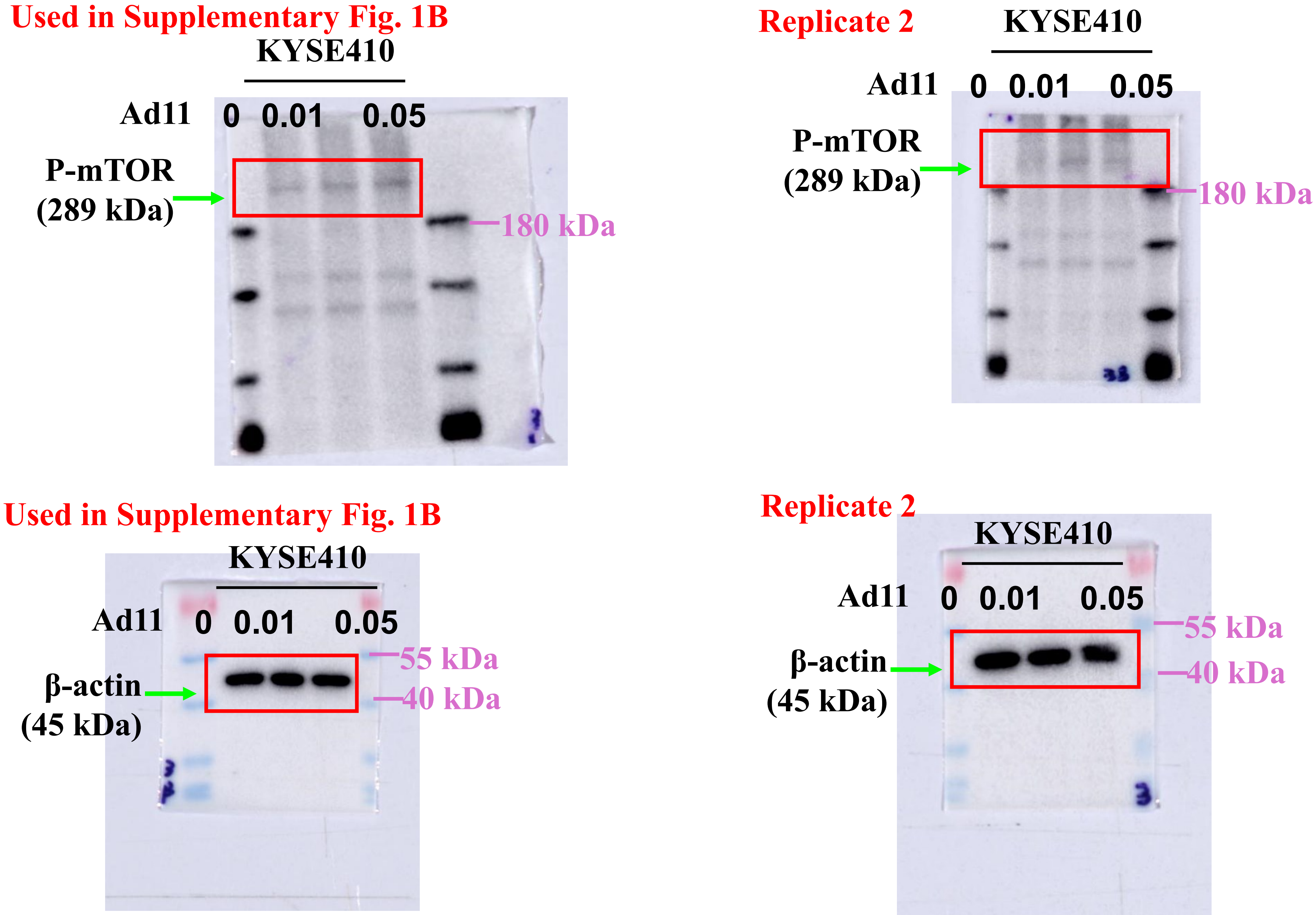

Supplementary Fig. 40: Complete uncropped western blot data of Supplementary Fig. 1B

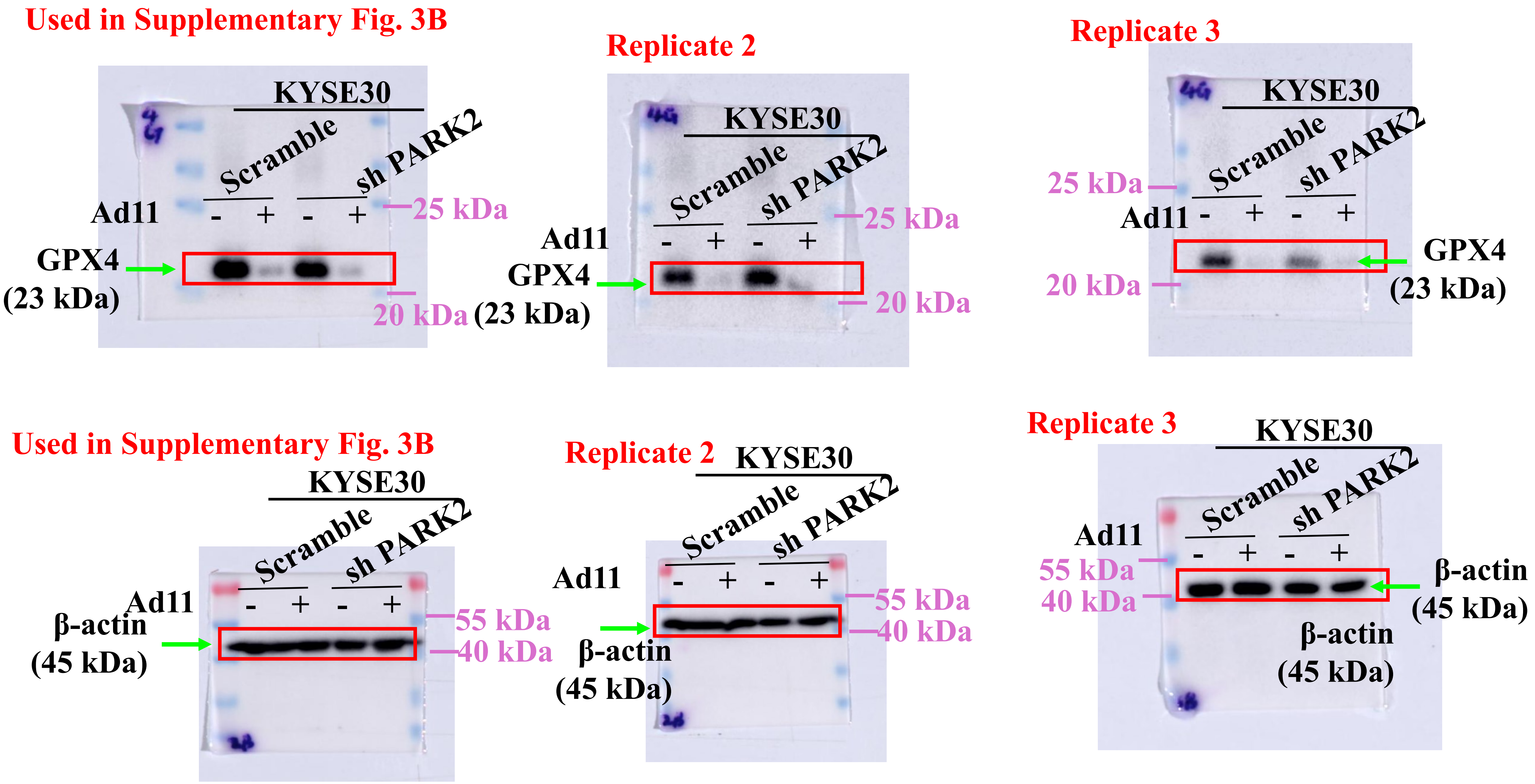

Supplementary Fig. 41: Complete uncropped western blot data of Supplementary Fig. 3B

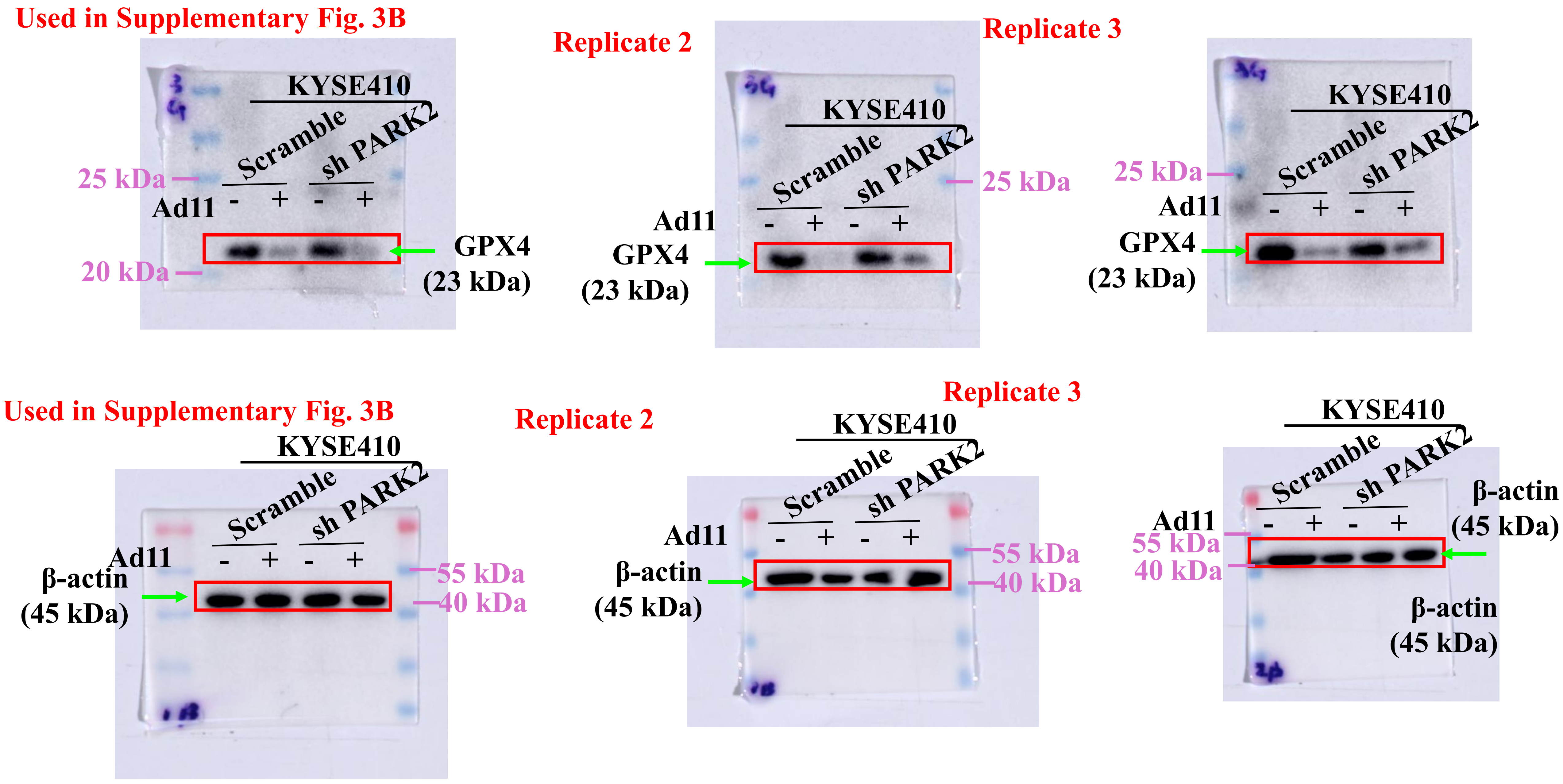

Supplementary Fig. 42: Complete uncropped western blot data of Supplementary Fig.3B

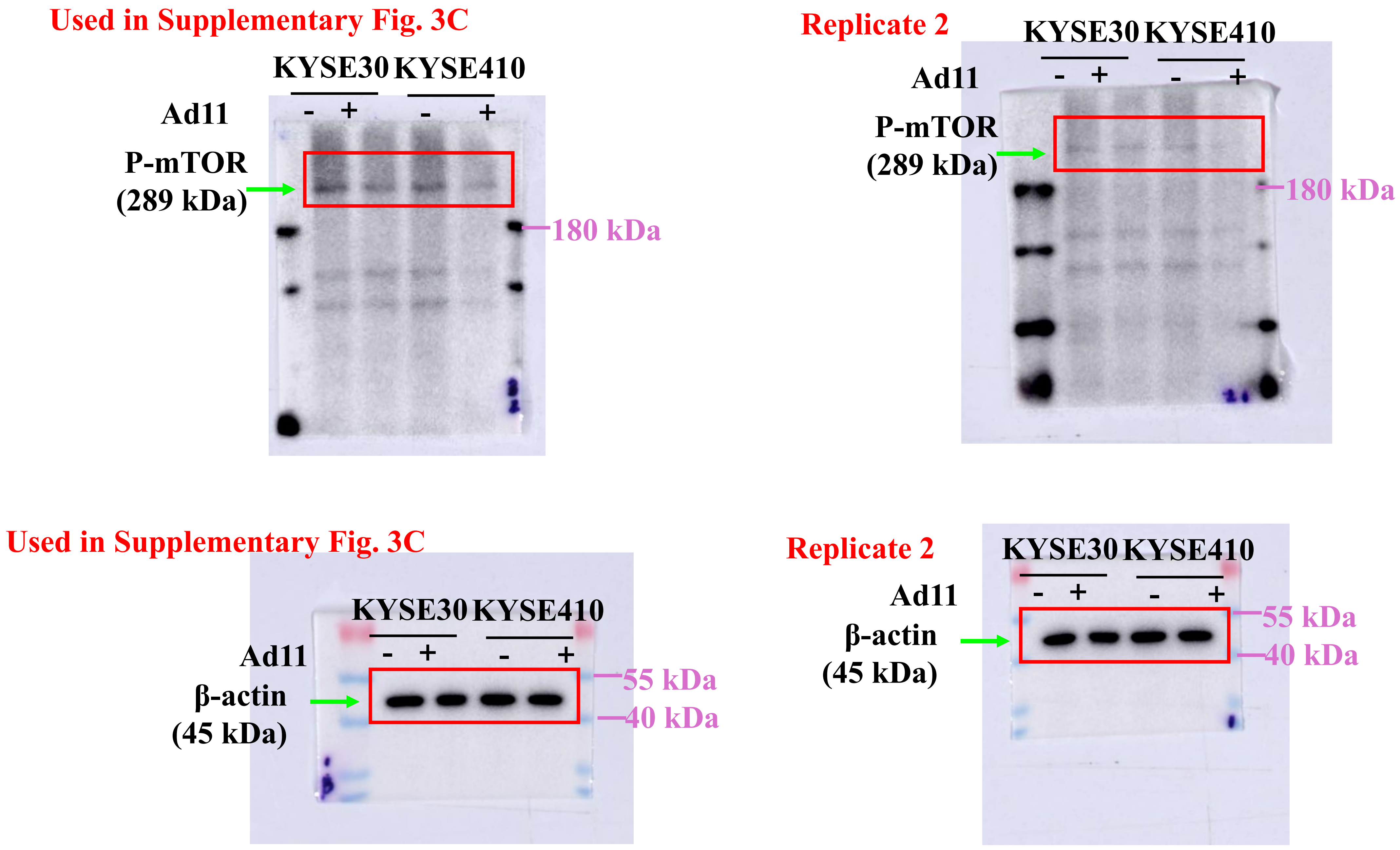

Supplementary Fig. 43: Complete uncropped western blot data of Supplementary Fig.3C
